# An evolutionarily conserved N-terminal domain of RRF-3 governs GTSF-1 binding in nematodes

**DOI:** 10.1038/s44319-026-00914-1

**Published:** 2026-08-29

**Authors:** Shamitha Govind, Sebastian Ruppert, Joseph Kirangwa, Virginia Busetto, Emily Nischwitz, Miguel Vasconcelos Almeida, Svenja Hellmann, Hahn Witte, Ralf J Sommer, Falk Butter, Sebastian Falk, Peter Sarkies, René F Ketting

**Affiliations:** 1 Biology of Non-coding RNA Group, Institute of Molecular Biology, Mainz, Germany; 2 International PhD Programme on Gene Regulation, Epigenetics & Genome Stability, Mainz, Germany; 3 Department of Biochemistry, University of Oxford, London, UK; 4 Max Perutz Labs, Vienna BioCenter, Vienna, Austria; 5 University of Vienna, Vienna, Austria; 6 Quantitative Proteomics Group, Institute of Molecular Biology, Mainz, Germany; 7 Department of Biochemistry, University of Cambridge, Cambridge, UK; 8 Department for Integrative Evolutionary Biology, Max Planck Institute for Biology, Tübingen, Germany; 9 Institute of Molecular Virology and Cell Biology, Friedrich Loeffler Institute, Greifswald, Germany; 10 Institute of Developmental Biology and Neurobiology, Johannes Gutenberg University, Mainz, Germany

**Keywords:** Evolution & Ecology, RNA Biology

## Abstract

GTSF1 is an essential activating cofactor for PIWI proteins in many metazoans. In the nematode *Caenorhabditis elegans*, however, GTSF-1 does not bind PIWI, but is associated with the RNA-dependent RNA polymerase RRF-3, supporting endo-siRNA (26G-RNA) biogenesis. Here, we demonstrate that this rewiring is deeply conserved across nematodes. For *C. briggsae* and *Pristionchus pacificus*, we show that GTSF-1 interacts with RRF-3 and is essential for 26G-RNA production and fertility. We map this interaction to an N-terminal domain of RRF-3, termed the GTSF-1 interacting domain (GID), and show that the GTSF-1 zinc finger region alone is sufficient for binding. Mutagenesis identifies critical residues mediating this interaction and reveals that GTSF-1 stability depends on RRF-3. Other RdRPs possess GID-like domains, which we propose to bind GTSF-1-related proteins. Phylogenomic and structural analyses support GTSF-1–RRF-3 interactions across all major nematode lineages and map the shift in GTSF-1 activity to the last common nematode ancestor. We propose that binding of GTSF-1 induces conformational changes in RRF-3 that facilitate RdRP complex assembly and activate its function, paralleling its role as a PIWI activator.

## Synopsis

GTSF1 activates PIWI in metazoans, yet in *C. elegans* it associates with the RdRP RRF-3. This study shows conservation of the GTSF-1-RRF-3 interaction across Nematoda, mediated by an RRF-3 N’ domain and reveals mechanistic plasticity within RNAi pathways.

- The tandem zinc-finger GTSF-1 protein binds the RNA-Dependent RNA Polymerase (RdRP) RRF-3 in clade V nematodes *C. elegans*, *C. briggsae* and *P. pacificus* to support 26G-siRNA biogenesis and fertility.
- GTSF-1 engages RRF-3 at a conserved N-terminal “GTSF-1 Interacting Domain (GID)”, with its zinc finger region alone sufficient to mediate this interaction.
- AlphaFold-Multimer modeling shows selective binding of GTSF-1 with RRF-3 over PIWI in species across clades I-V, tracing the evolutionary switch in GTSF-1 partnering from PIWI to RdRP to the last common nematode ancestor.

## Abstract (web summary)

GTSF1 activates PIWI in metazoans, yet in *C. elegans* it associates with the RdRP RRF-3. This study shows conservation of the GTSF-1-RRF-3 interaction across Nematoda, mediated by an RRF-3 N’ domain and reveals mechanistic plasticity within RNAi pathways.

## Graphical abstract

**Figure:**
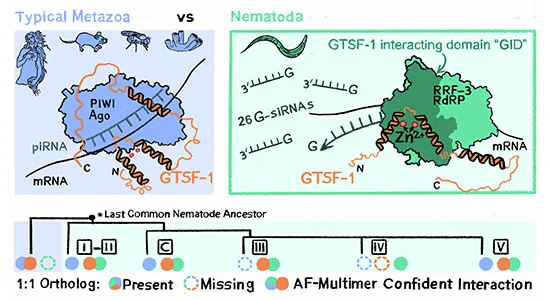

## Introduction

RNA interference (RNAi) represents an ancient regulatory mechanism that employs antisense RNAs to modulate gene expression. Conserved across prokaryotes and eukaryotes, RNAi pathways serve dual roles in genome defense against viruses and transposons, and in developmental regulation of gene expression. In metazoans, RNAi pathways are classified into three distinct branches: the microRNA (miRNA), small interfering RNAs (siRNAs), and PIWI-interacting RNA (piRNA) pathways. RNAi machinery is composed of three key enzymatic activities: RNA-dependent RNA polymerases (RdRPs) that synthesize double-stranded RNA (dsRNA), RNase III enzymes such as Dicer that process dsRNA into 20–30 nucleotide siRNA, and Argonaute/Piwi proteins, which are RNase H-like enzymes that utilize these small RNAs as sequence-specific guides to cleave or translationally repress target mRNAs.

Phylogenetic analyses suggest that the last common eukaryotic ancestor possessed all these three core RNAi components (Cerutti and Casas-Mollano, 2006; Shabalina and Koonin, 2008). However, gene duplications, losses, and functional innovations have diversified RNAi architecture across eukaryotic lineages. Plants and fungi, for instance, lack the PIWI-piRNA pathway but have evolved specialized RdRP-Dicer-Argonaute pathways that generate and amplify siRNAs (Krzyszton et al, 2025). Conversely, most metazoans have lost RdRPs, instead deriving their siRNAs from naturally occurring or exogenously introduced dsRNAs (Claycomb, 2014) or from single-stranded precursors (Ozata et al, 2019). In metazoan lineages, Piwi-piRNA pathways have become specialized for transposon silencing in the germline (Ozata et al, 2019). However, certain parasitic flatworms, for example, *S mansoni* and *E. ocularis*, the dust mite *S. scabei*, and most nematodes have independently lost the PIWI-piRNA pathway (Fontenla et al, 2021; Sarkies, 2024; Sarkies et al, 2015). In contrast, nematodes and chelicerates (spiders, horseshoe crabs, scorpions) retain functional RdRP genes (Sarkies, 2024; Sarkies et al, 2015; Lewis et al, 2018; Mondal et al, 2018b, 2018a). Despite these insights, our understanding of RNAi pathway evolution remains incomplete. While the core RNAi enzymes are well characterized, the auxiliary factors that modulate pathway specificity and efficiency are poorly understood, limiting our comprehension of how RNAi systems adapt and innovate across evolutionary time.

One such auxiliary factor is Gametocyte-specific factor 1 (GTSF1/Gtsf1), a conserved PIWI cofactor that was identified as essential for piRNA function and fertility in mice, flies, and silk moths (Ohtani et al, 2013; Dönertas et al, 2013; Izumi et al, 2022; Bronkhorst et al, 2023; Arif et al, 2022; Yoshimura et al, 2009, 2018). GTSF1 proteins, which typically are between 20 and 30 kDa in mass, contain two tandem CHHC-type zinc fingers, each forming an independent fold around a central zinc ion. This structural motif is also present in TRM13 tRNA modification enzymes and the minor spliceosomal protein U11-48K (Andreeva and Tidow, 2008). GTSF1 proteins are absent from fungi and plants but are deeply conserved across Metazoa, with many lineages encoding multiple paralogs (Takemoto et al, 2016; Izumi et al, 2022; Ipsaro and Joshua-Tor, 2022; Kina et al, 2025). Biochemical reconstitution experiments with Piwi proteins from mice, flies, and silk moths have revealed that GTSF1 binds PIWI proteins engaged with target mRNA and induces conformational changes that generate catalytically active PIWI* complexes. For cytoplasmic Piwi proteins such as *M. musculus* MIWI and MILI or *B. mori* Siwi and Ago3, GTSF1 stabilizes a cleavage-competent conformation that facilitates efficient target mRNA slicing (Arif et al, 2022; Izumi et al, 2022; De et al, 2025). For catalytically inactive Piwi proteins, such as nuclear Piwi in *D. melanogaster*, GTSF1 binding creates protein–protein interaction surfaces that recruit chromatin-modifying complexes for transcriptional silencing (Portell-Montserrat et al, 2025).

The nematode *Caenorhabditis elegans* encodes a large complement of Argonaute proteins, including the canonical Piwi protein PRG-1 (Bagijn et al, 2012; Das et al, 2008; Seroussi et al, 2023; Wang and Reinke, 2008). PRG-1 binds piRNAs (termed 21U-RNAs in *C. elegans*), but unlike other metazoan Piwi proteins, it targets the entire germline transcriptome, including protein-coding genes and transposons (Das et al, 2008; Bagijn et al, 2012; Shen et al, 2018). Loss of PRG-1 is not lethal but causes progressive fertility defects across generations (Batista et al, 2008). Surprisingly, *C. elegans* GTSF-1 does not interact with PRG-1 or participate in the piRNA pathway. Instead, GTSF-1 is associated with RRF-3, an RdRP that synthesizes 26G-RNAs, a distinct class of endogenous siRNA (Almeida et al, 2018). In *C. elegans*, GTSF-1 is required to assemble RRF-3 into a multiprotein RdRP complex named ERIC (ERI Complex), which includes Dicer, thereby coupling dsRNA synthesis with Dicer processing (Almeida et al, 2018; Duchaine et al, 2006). These 26G-RNAs subsequently load onto the Argonautes ALG-3/4 and ERGO-1 in the male and female germlines, respectively, to target protein-coding genes and pseudogenes (Vasale et al, 2010; Conine et al, 2010).

Interestingly, RRF-3 orthologs are widespread throughout the phylum Nematoda, whereas PRG-1 orthologs are often absent (Sarkies et al, 2015). In some species of nematodes, RRF-3-like RdRPs have been proposed to functionally replace the lost piRNA pathway by synthesizing siRNAs complementary to transposon sequences (Sarkies et al, 2015). Indeed, nematode RNAi systems are heavily reliant on small RNA amplification by RdRPs. Unlike canonical metazoan RNAi systems, Argonaute-bound 21U-RNAs and 26G-RNAs in *C. elegans* do not directly silence targets. Rather, they trigger the RdRPs RRF-1 and EGO-1 to synthesize abundant secondary siRNAs termed 22G-RNAs (Batista *e*t al, 2008; Maniar and Fire, 2011; Kumsta and Hansen, 2012; Gent et al, 2010; Sijen et al, 2001). These 22G-RNAs then load onto nematode-specific WAGO Argonautes to induce transcriptional silencing and chromatin modification (Ketting and Cochella, 2021; Seroussi et al, 2023; Gent et al, 2010).

Within this intricate architecture of the RNAi pathway, the molecular determinants of *C. elegans* GTSF-1 specificity for RRF-3 remain unclear. More fundamentally, the evolutionary origins that redirected *C. elegans* GTSF-1 from PIWI binding to RRF-3 binding are not understood. To address these questions, we performed a comparative analysis of GTSF-1 function in the nematodes *Caenorhabditis briggsae* and *Pristionchus pacificus*. Despite having diverged from *C. elegans* ~100 and 300 million years ago, respectively, these species express 21U-, 22G-, and 26G-RNAs as well as the protein components of their respective pathways (Dieterich et al, 2008; Pastore et al, 2022; Shi et al, 2013; Stein et al, 2003). This set of species provides an opportunity to probe the functional conservation of nematode GTSF-1 across deep evolutionary timescales.

In this work, we demonstrate that *C. briggsae* and *P. pacificus* GTSF-1 retain robust, specific association with RRF-3 and are essential for RRF-3-dependent 26G-RNA biogenesis. The zinc finger region of nematode GTSF-1 alone is sufficient for RRF-3 binding. We find GTSF-1 to interact with an N-terminal region of RRF-3, which we designate the GTSF-1 interacting domain (GID). We detect GTSF-1 and RRF-3 orthologs across the nematode phylum, and analysis of the predicted complex structures supports specific GTSF-1-RRF-3 interactions in multiple nematode species but no interaction with orthologs of PRG-1. Notably, structural predictions from a nematode sister lineage reveal a strong PIWI-GTSF-1 interaction, mapping the origin of RdRP binding to the nematode ancestor. We propose a conserved function for GTSF-1 to activate RRF-3 by inducing conformational rearrangements and promoting recruitment of the ERIC. Finally, this work highlights a striking case of process homology within RNAi pathways, illustrating how RNAi components can diverge mechanistically while maintaining the overall regulatory outcomes of gene silencing and genome defense.

## Results

### Nematode GTSF-1 is required for fertility and the 26G-RNA pathway

**Figure 1:**
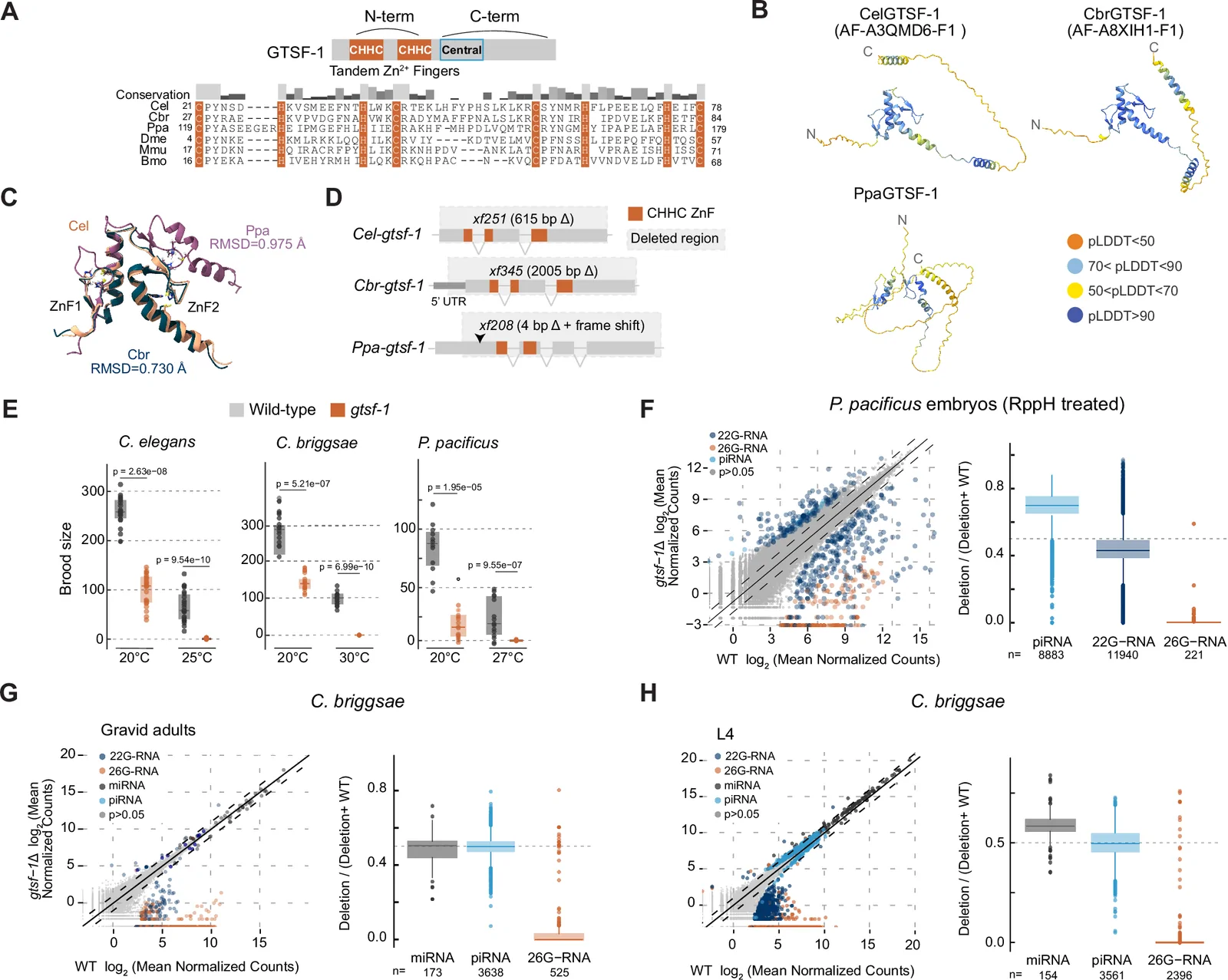
GTSF-1 is essential for fertility and 26G-RNA biogenesis. (**A**) Domain architecture of GTSF-1 showing the N-terminal (N-term) zinc finger region, the central region, and the C-terminal (C-term) region. Multiple sequence alignment (MSA) of residues spanning the CHHC zinc fingers in various GTSF-1 orthologs was performed using Clustal Omega and visualized with Jalview. The histogram above the alignment indicates conservation of physico-chemical properties in the MSA based on the AMAS method (Livingstone and Barton, 1993). Species: Cel, *C. elegans*; Cbr, *C. briggsae*; Ppa, *P. pacificus*; Dme, *D. melanogaster*, Mmu, *M. musculus*; Bmo, *B. mori*. (**B**) AlphaFold2 monomer structure prediction of GTSF-1 colored by predicted local distance difference test (pLDDT) score (0–100). *C. elegans* and *C. briggsae* GTSF-1 were downloaded from the AlphaFold database (AF-A3QMD6-F1 and AF-A8XIH1-F1, respectively) while *P. pacificus* GTSF-1 was modeled in-house. (**C**) Alignment of zinc finger (ZnF) regions of GTSF-1. Species: Cel, *C. elegans*; Cbr, *C. briggsae*; Ppa, *P. pacificus*. CelGTSF-1 residues 11–92 are superimposed with CbrGTSF-1 residues 17–98 (Cα RMSD = 0.730 Å) and PpaGTSF-1 residues 110–194 (Cα RMSD = 0.975 Å). (**D**) Schematic of *gtsf-1* deletion alleles. Gray-filled boxes indicate exons and 5’UTR. Gray dotted boxes mark genomic regions deleted by CRISPR/Cas9. The black arrow shows the position of a 4-bp deletion in *Ppa-gtsf-1* that results in frameshift and premature termination. (**E**) Brood size of wild-type (WT) and *gtsf-1* deletion animals at 20 °C or 25–30 °C (*n* = 15–25 per genotype) as box plots. Box plots show the median, 25th–75th percentiles (box), and whiskers extending to the most extreme value within 1.5× the interquartile range, with outliers shown as individual points. *P* values: Wilcoxon rank-sum test with Benjamini–Hochberg (BH) correction. (**F**–**H**) Small-RNA profiles in wild-type and *gtsf-1* deletion animals. (**F**) *P. pacificus embryos*; RppH-treated libraries. (**G**) *C. briggsae* gravid-adults; Untreated libraries. (**H**) *C. briggsae* L4 animals; Untreated libraries. Left: Normalized counts (DESeq2) averaged across three biological replicates. *P* values calculated using the Wald test. Colored points: *P* < 0.05. Solid line: equal expression; dotted lines: ±2-fold change. Right: Normalized count abundance per gene by small-RNA type as box plots. Data includes three biological replicates of each sample. Abundance calculated as counts in deletion samples divided by total counts. Box plots show the median, 25th–75th percentiles (box), and whiskers extending to the most extreme value within 1.5× the interquartile range, with outliers shown as individual points; *n* = number of genes per category. Source data are available online for this figure.

**Figure EV1:**
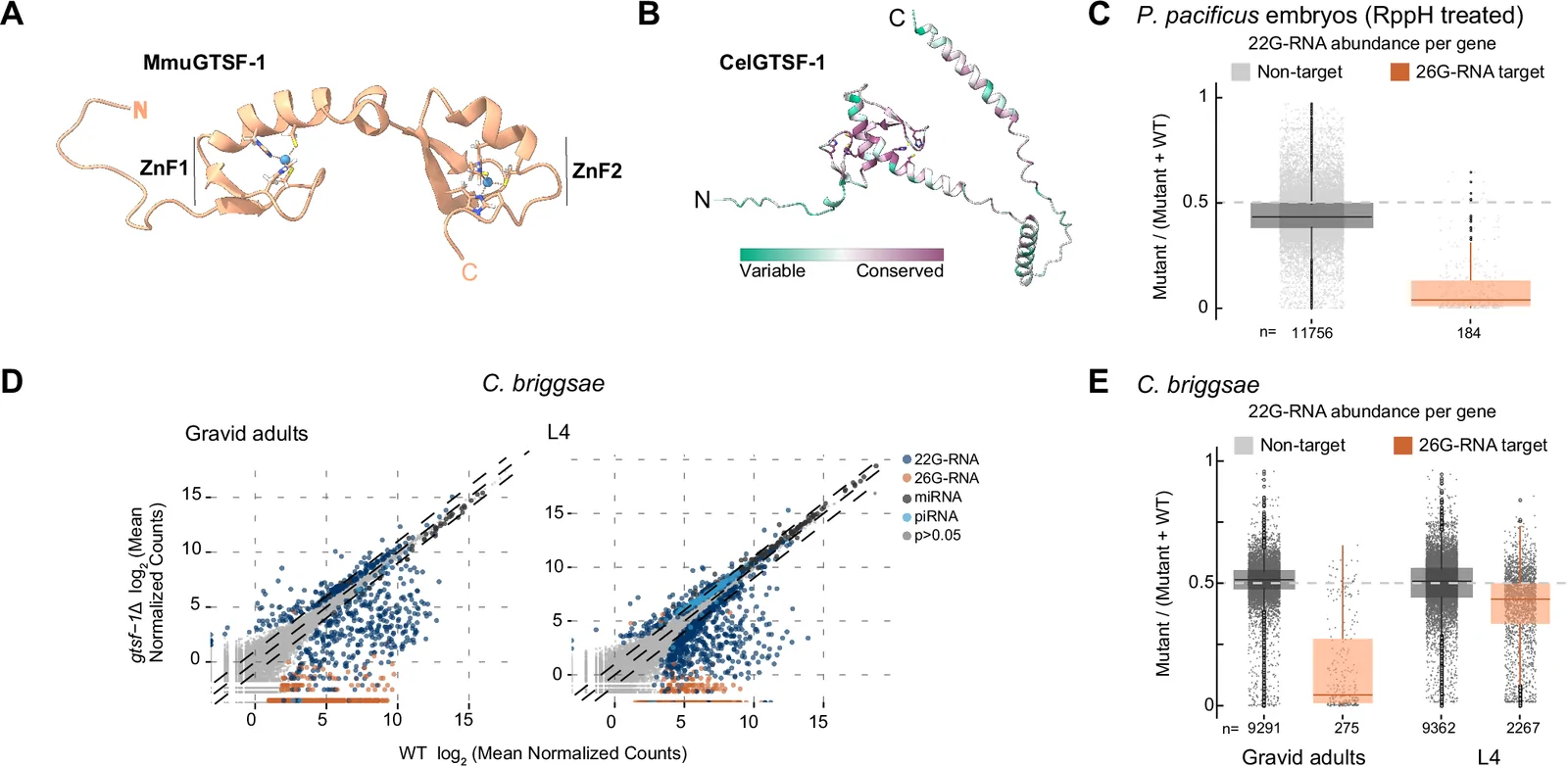
GTSF-1 structure prediction and RppH-treated small RNA sequencing in *gtsf-1* mutants. (**A**) *M. musculus* NMR structure (NMR_6X46L (Ipsaro et al, 2021)). CHHC residues comprising zinc fingers are displayed in stick format. (**B**) AlphaFold2 monomer prediction of GTSF-1 colored by ConSurf conservation score (1:Variable to 9:Conserved) calculated from an MSA of 26 GTSF-1 orthologs (*C. elegans, C. briggsae, C. inopinata, P. pacificus, C. brenneri, C. castelli, C. doughertyi, C. japonica, C. kamaaina, C. nouraguensis, C. plicata, C. sp21, C. sp26, C. sp28, C. sp29, C. sp31, C. sp38, C. sp39, C. sp40, C. tropicalis, C. virilis, C. nigoni, Toxocara canis, Brugia malayi, Wuchereria bancrofti, Brugia pahangi)*. ‘N’ and ‘C’ denote N and C-terminal residue. (**C**) *P. pacificus* embryos RppH-treated libraries: Abundance of DESeq2-normalized 22G-RNA counts per gene between wild-type and *gtsf-1* deletion samples as box plots. Abundance calculated as counts in deletion samples divided by total counts. *n* = number of genes per category. Box plots show the median, 25th–75th percentiles (box), and whiskers extending to the most extreme value within 1.5× the interquartile range, with data shown as jitter points and outliers as individual points. (**D**, **E**) RppH-treated libraries comparing wild-type and *gtsf-1* deletion animals in *C. briggsae* gravid adults and L4 larvae. (**D**) Normalized counts (DESeq2) averaged across three biological replicates depicted as a scatter plot. *P* value calculated using the Wald test. Colored points: *P* < 0.05. Solid line: equal expression; dotted lines: ±2-fold change. (**E**) DESeq2-Normalized 22G-RNA count abundance per gene for 26G-RNA target or non-target genes. Data includes three biological replicates of each sample. Box plots show the median, 25th–75th percentiles (box), and whiskers extending to the most extreme value within 1.5× the interquartile range, with data shown as jitter points and outliers as individual points. Source data are available online for this figure.

Multiple GTSF1 paralogs are found across animal species (Ipsaro and Joshua-Tor, 2022). For example, *D. melanogaster* encodes four (Asterix/Gtsf1, CG14036, CG32625, and CG34283) (Dönertas et al, 2013; Ohtani et al, 2013; Ipsaro et al, 2021; Portell-Montserrat et al, 2025; Kina et al, 2025), *M. musculus* has three (GTSF1, GTSF1-like, and GTSF2) (Yoshimura et al, 2018, 2009; Arif et al, 2022) and the silkmoth *B. mori* has two paralogs (Gtsf1 and Gtsf1-like) (Izumi et al, 2022; Bronkhorst et al, 2023; Chen et al, 2025, 2020). In contrast, *C. elegans* (Cel), *C. briggsae* (Cbr), and *P. pacificus* (Ppa) each possess a single ortholog of GTSF1, based on reciprocal BLAST search and current annotation. These nematode GTSF-1 orthologs possess the characteristic tandem CHHC zinc fingers in their N-terminal region. Their C-terminal tails are approximately 100 amino acids long and retain the ‘central’ region, which is essential for Piwi binding in orthologs from *M. musculus* and *D. melanogaster* (Dönertas et al, 2013; Yoshimura et al, 2018) (Fig. 1A). AlphaFold2 (AF2) predicted structures of these orthologs closely match the solution structure of mouse GTSF1 (Ipsaro et al, 2021) (Figs. 1B and  EV1A). The tandem zinc fingers are arranged within two α-helices, connected by a flexible linker and the C-terminal region consists of α-helices interspersed by disordered stretches (Fig. 1B,C). In addition, PpaGTSF-1 contains an intrinsically disordered region (IDR) of ~100 amino acids at its N-terminus (Fig. 1B). As observed for metazoan GTSF1/Gtsf1 proteins (Ipsaro and Joshua-Tor, 2022), the zinc finger regions of nematode GTSF-1 are more conserved than the disordered C-terminal region (Fig. EV1B).

In our previous work, we demonstrated that *C. elegans* GTSF-1 is essential for the 26G-RNA pathway (Almeida et al, 2018). Mutants defective in this pathway exhibit impaired spermatogenesis, resulting in reduced brood size and sterility at elevated temperatures. To study this further, we generated *gtsf-1* mutant alleles in *C. elegans*, *C. briggsae*, and *P. pacificus* using CRISPR/Cas9 (Fig. 1D). In *C. briggsae* and *C. elegans*, we created a complete gene deletion. In *P. pacificus*, our alleles preserve the first 100 amino acids in-frame before introducing a 4 base-pair deletion resulting in a premature stop codon. We first tested how these *gtsf-1* alleles affect animal fertility. The *gtsf-1* deletion allele in *C. elegans* showed reduced brood size at 20 °C and sterility at 25 °C (Fig. 1E). Similar to this, *C. briggsae* and *P. pacificus* animals with *gtsf-1* deletion alleles produced severely low brood sizes at 20 °C and were sterile at stressful temperatures of 30 °C and 27 °C, respectively (Fig. 1E).

In *C. elegans, gtsf-1* mutant animals are completely devoid of 26G-RNAs (Almeida et al, 2018). Thus, our next step was to profile small RNAs in *gtsf-1* mutant nematodes. For *P. pacificus*, we generated small-RNA libraries from embryos of wild-type and *gtsf-1* deletion animals, during which the extracted RNA was treated with RNA 5´Pyrophosphohydrolase (RppH). RppH converts 5′-triphosphorylated small RNAs to 5′-monophosphorylated RNAs, enabling their ligation to adapters during library preparation. This treatment procedure enriches the population of 5′-triphosphorylated small RNAs, such as the 22G-RNAs in *C. elegans*, and allows for a simultaneous comparison of all small-RNA classes. The analyzed *P. pacificus* libraries showed a complete depletion of 26G-RNAs in *gtsf-1* deletion samples compared to wild-type (Fig. 1F). Genome-wide levels of 22G-RNAs remained unchanged upon *gtsf-1* deletion, however, the subset of 22G-RNAs that mapped to 26G-RNA target genes were substantially lost (Fig. EV1C). Interestingly, we also observed an increase in piRNAs, but this was not substantial (Fig. 1F).

For *C. briggsae*, we additionally profiled developmental stage-specific small RNAs. The 26G-RNAs of *C. elegans* are expressed in the germline but form distinct classes in spermatogenic gonads compared to oogenic and embryonic gonads (Han et al, 2009; Seroussi et al, 2023; Vasale et al, 2010; Conine et al, 2010). We previously demonstrated that *gtsf-1* deletion in *C. elegans* eliminates both classes of 26G-RNAs (Almeida et al, 2018). To test this in *C. briggsae*, we sequenced small RNAs from gravid adults, representing the oogenic germ line and embryos, and L4 larvae, representing the spermatogenic germ line. We sequenced both untreated RNA libraries to test for 5’ monophosphorylated RNA levels (miRNAs, 21U-RNAs, 26G-RNAs) and RppH-treated libraries to additionally enrich for 5’ triphosphorylated 22G-RNAs. Our analysis of untreated libraries showed that loss of *gtsf-1* in *C. briggsae* eliminated 26G-RNAs from both developmental stages, while piRNAs and miRNAs were largely unchanged (Fig. 1G,H). In addition, RppH-treated libraries showed unchanged genome-wide 22G-RNA levels but a strong depletion of 22G-RNAs downstream of 26G-RNA target genes, particularly in gravid adults (Fig. EV1D,E). Taken together, these results demonstrate a strong and specific role of *C. briggsae* and *P. pacificus* GTSF-1 in the 26G-RNA pathway.

**Figure EV2:**
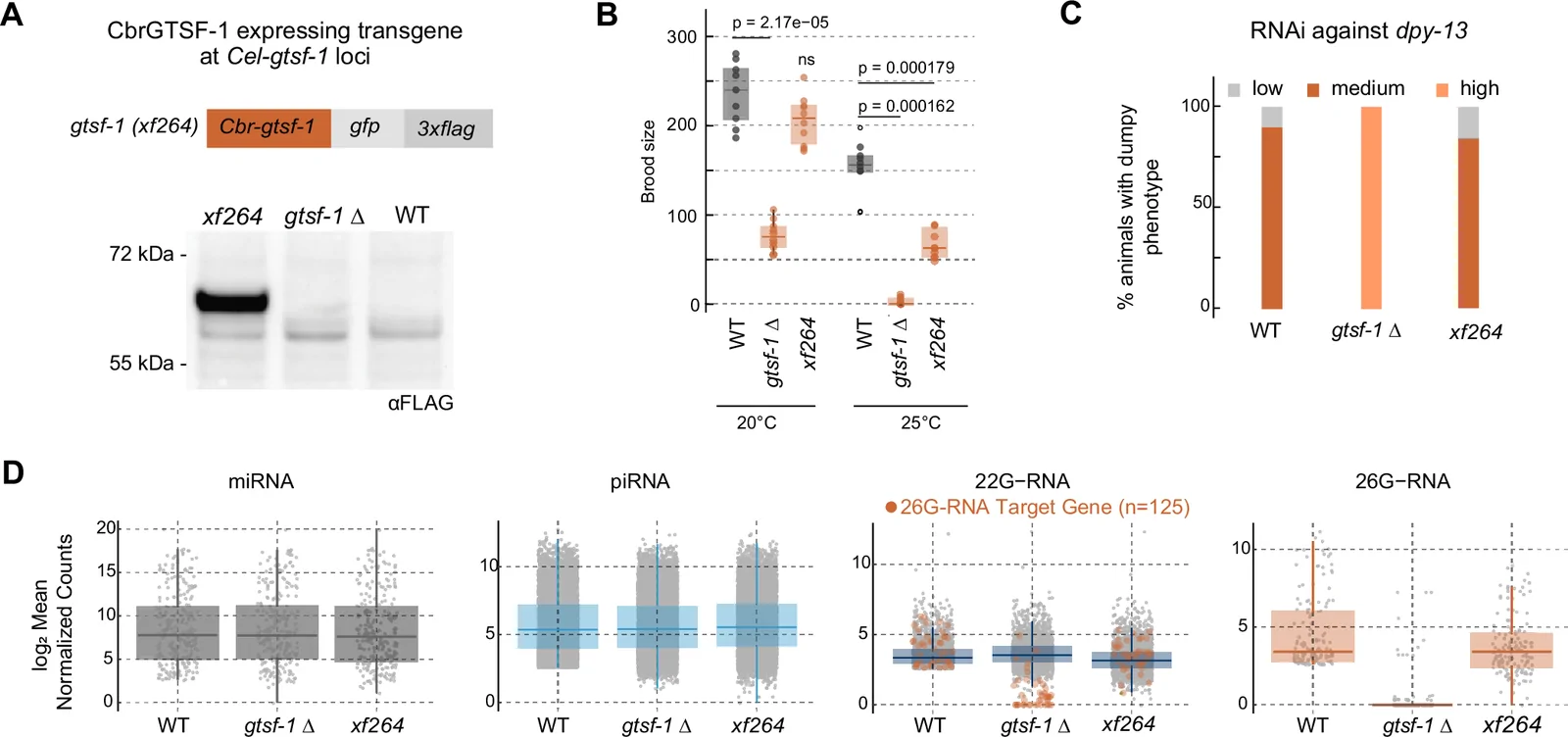
Transgenic *C. briggsae gtsf-1* partially rescues *C. elegans gtsf-1* deletion. (**A**) Top: Schematic of *Cel-gtsf-1(xf264)* allele with endogenous *gtsf-1* replaced by *C. briggsae gtsf-1* transgene upstream of *gfp* and *3×flag*. Bottom: Western blot from gravid adults validating transgene expression. (**B**) Brood size quantification (*n* = 15–25) as box plots. Box plots show the median, 25th–75th percentiles (box), and whiskers extending to the most extreme value within 1.5× the interquartile range, with data shown as jitter points and outliers as individual points. *P* values: Wilcoxon rank-sum test with BH correction. ns: Non-significant compared to wild-type. (**C**) RNAi responses to *dpy-13* scored by *dumpy* phenotype penetrance and severity (low, medium, high) based on the visual ratio of body length to thickness. High severity: L2-like length with extreme thickening causing immobility. (**D**) Small-RNA profiles for *C. elegans gtsf-1* mutant animals (Gravid adults; Untreated libraries). Plot displays normalized counts per gene for small-RNA types. DESeq2-normalized counts averaged across three replicates. Colored 22G-RNA counts: 26G-RNA target genes identified in this dataset. Data includes three biological replicates of each sample. Source data are available online for this figure.

In a cross-species complementation approach, we expressed *C. briggsae* GTSF-1 in a *C. elegans gtsf-1* deletion strain through a transgene and found it to partially rescue fertility defects as well as overall levels of 26G-RNAs and their downstream 22G-RNAs (Fig. EV2A–D). Interestingly, the rescuing effect on brood-size was stronger at 20 °C than 25 °C. An important phenotype observed in *C. elegans* mutants for the 26G-RNA pathway is the “Eri” (Enhanced RNAi) phenotype. These animals show a “hypersensitivity” to RNAi, characterized by an enhanced silencing of somatic genes (Duchaine et al, 2006; Simmer et al, 2002). In *C. elegans*, *gtsf-1* mutant animals exposed to *dpy-13* RNAi show an extreme *dumpy* phenotype, in which the animal is smaller and rounder than a usual *dumpy* (Almeida et al, 2018). Indeed, the expression of *C. briggsae* GTSF-1 partially rescued the Eri phenotype in *C. elegans*, again demonstrating functional conservation.

We conclude that the phenotypes described for *gtsf-1* loss in *C. elegans* are conserved in *C. briggsae* and *P. pacificus*.

### Nematode GTSF-1 associates with RRF-3

In *C. elegans*, RRF-3 and GTSF-1 are part of the ERI Complex (Almeida et al, 2018). ERIC synthesizes 26G-RNAs through a coordinated multi-step process. The current hypothesis is that a NYN domain nuclease, RDE-8 or NYN-3, initiates the pathway by cleaving target RNAs upstream of RRF-3-mediated dsRNA synthesis (Tsai et al, 2022, 2015). RRF-3, assisted by the Tudor domain protein ERI-5 and the DExD/H-box helicase DRH-3, generates long double-stranded RNAs (Aoki et al, 2007; Duchaine et al, 2006; Gent et al, 2009; Han et al, 2009; Lee et al, 2006; Matranga and Pyle, 2010; Pavelec et al, 2009; Thivierge et al, 2012). These dsRNAs are subsequently processed by Dicer, phosphatase PIR-1, and DEDDh-like exonuclease ERI-1 into 26-nucleotide RNAs with a 5’ monophosphate (Chaves et al, 2021; Gabel and Ruvkun, 2008; Welker et al, 2010). Additional factors, including RDE-4 and ERI-3, also participate in this process (Duchaine et al, 2006; Tabara et al, 2002). Immunoprecipitation coupled with label-free quantitative mass spectrometry (IP-MS) on GTSF-1 in *C. elegans* adult extracts readily detects RRF-3 (Almeida et al, 2018). IP-MS of epitope-tagged GTSF-1 expressed via a transgene additionally enriches for ERI-5 and DRH-3 (Almeida et al, 2018).

**Figure 2:**
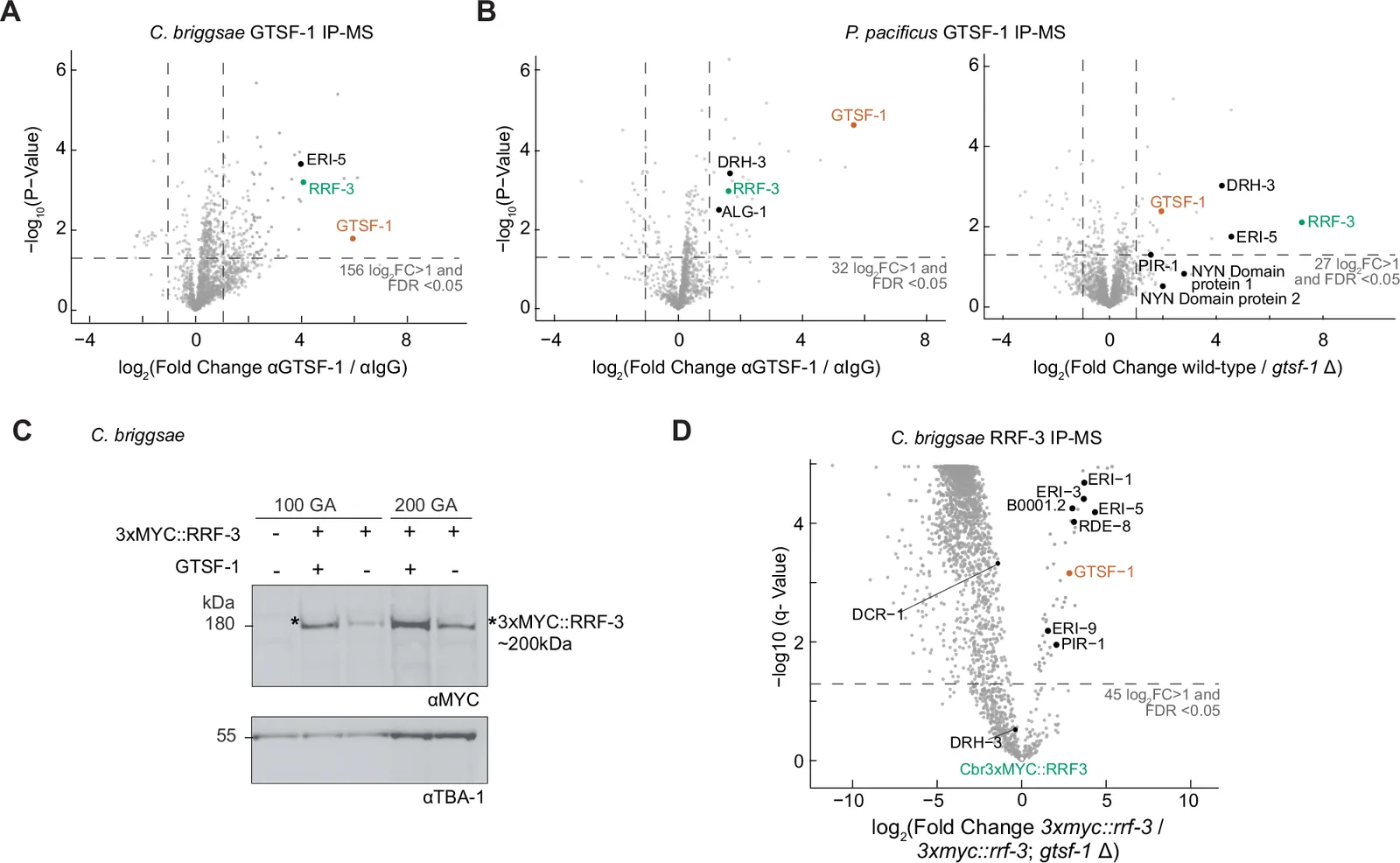
GTSF-1 interacts with RRF-3 in the ERI complex. (**A**, **B**) Co-immunoprecipitation coupled to quantitative mass spectrometry (Co-IP/MS) of GTSF-1 on gravid adult animal lysates. Dotted lines: log₂ Fold Change ±1 and *P* < 0.05 (Welch’s *t* test). *n* = 4 biological replicates per sample. ERI complex and ALG-1 homologs highlighted. (**A**) *C. briggsae*; IP with anti-CbrGTSF-1 against Rabbit-IgG in wild-type animals (**B**)* P. pacificus*; Left: IP with anti-PpaGTSF-1 against Rabbit-IgG in wild-type animals. Right: IP with anti-PpaGTSF-1 in wild-type and *gtsf-1* Δ animals. (**C**) Western blot with anti-cMYC in *C. briggsae* wild-type, *3×myc::rrf-3* or *3×myc::rrf-3; gtsf-1 Δ* animals (100/200 gravid adults (GA) hand-picked and boiled). TBA-1: tubulin loading control. (**D**) Co-IP/MS using anti-cMYC magnetic beads on *C. briggsae* embryo lysates. Data normalized to 3×MYC::RRF-3 levels. *x* axis: log₂ Fold Change; *y* axis: Adjusted *P* values (*q* value or t(SAM) statistic (Tusher et al, 2001)). Dotted lines: Fold Change ±2. ERI complex homologs highlighted. *n* = 4 biological replicates per sample. Source data are available online for this figure.

Based on this information, we wanted to determine whether the GTSF-1-RRF-3 interaction is conserved across nematodes. To achieve this, we performed GTSF-1 IP-MS on adult extracts from *C. briggsae* and *P. pacificus*, using affinity-purified antibodies. The IP-MS for GTSF-1 in *C. briggsae* significantly enriched RRF-3 and ERI-5 (Fig. 2A). For *P. pacificus*, we performed two IP-MS experiments with different controls. Using anti-IgG antibodies as a negative control, GTSF-1 IP enriched RRF-3 and DRH-3, but not ERI-5 (Fig. 2B, left panel). This experiment also showed enrichment of the Argonaute ALG-1 (log₂FC = 1.89, −log₁₀ *P* value = 2.83), which is loaded with miRNAs in *C. elegans* (Grishok et al, 2001). To further validate antibody specificity, we compared GTSF-1 IPs from wild-type gravid adults to those from *gtsf-1* deletion animals. In this experiment, ERI-5 was now enriched in GTSF-1 IPs, in addition to RRF-3 and DRH-3 (Fig. 2B, right panel). Homologs of the 26G-RNA pathway components PIR-1 and two NYN domain proteins were also detected, although not significantly enriched (Fig. 2B, right panel). However, ALG-1 was no longer detected.

In *C. elegans*, GTSF-1 is required for coupling RRF-3 to ERIC (Almeida et al, 2018). Immunoprecipitation of RRF-3 from embryo extracts enriches all known ERIC proteins in wild-type animals, but only ERI-5 in *gtsf-1* mutants (Almeida et al, 2018). To test the conservation of this dependency, we generated epitope-tagged RRF-3 lines in *C. briggsae* and performed IPs in both wild-type and *gtsf-1* deletion backgrounds. Surprisingly, loss of *gtsf-1* caused a reduction in RRF-3 protein abundance (Fig. 2C), which was not observed previously for *C. elegans* (Almeida et al, 2018). To account for this, we normalized all protein abundances to RRF-3 levels when comparing genotypes. In RRF-3 IPs from wild-type *C. briggsae*, we detected several homologs of ERIC components-ERI-1, ERI-3, ERI-5, ERI-9, RDE-8, and PIR-1-whereas these proteins were absent in *gtsf-1* deletion samples (Fig. 2D). However, DRH-3 and DCR-1 were present in both conditions and DCR-1 even showed mild enrichment in *gtsf-1* deletion samples, suggesting that *C. briggsae* GTSF-1 is dispensable for RRF-3 interactions with DRH-3 and DCR-1. Since this pattern was not observed in *C. elegans* (Almeida et al, 2018), these findings reveal species-specific aspects of ERIC assembly.

These results establish that GTSF-1 selectively associates with RRF-3 in both *C. briggsae* and *P. pacificus*, with no detectable interactions with other RdRPs (RRF-1, EGO-1) or PIWI proteins. Further, the function of GTSF-1 in ERIC assembly remains conserved in *C. briggsae*, even though species-specific dependencies were detected.

### The C-terminal region of CelGTSF-1 is dispensable for binding RRF-3 and the ERI complex

**Figure 3:**
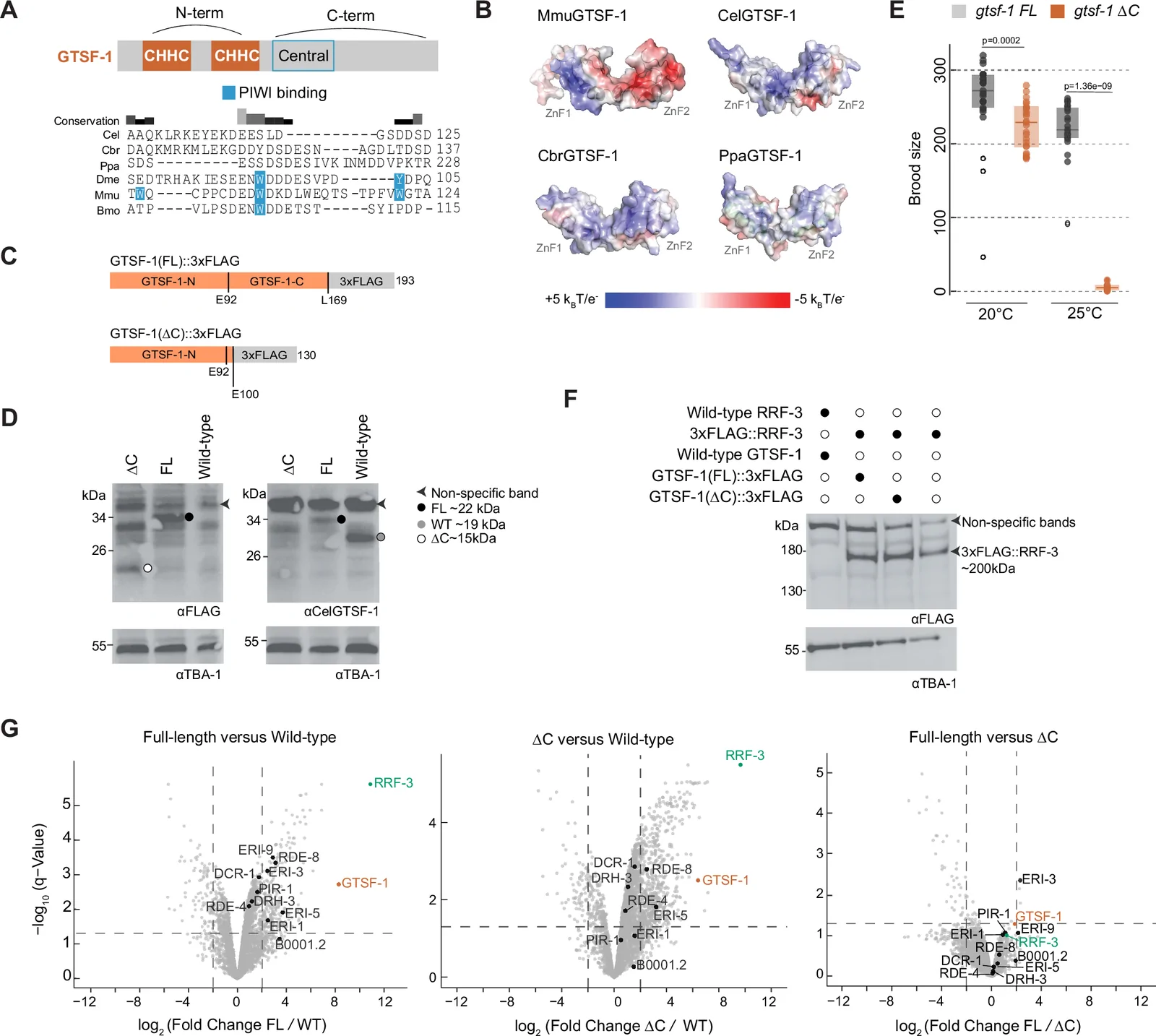
The C-terminal region of GTSF-1 is dispensable for RRF-3 interaction. (**A**) Domain architecture of GTSF-1. Clustal Omega MSA of the Central region from various GTSF1/Gtsf1 orthologs, highlighting aromatic residues implicated in PIWI binding. Visualization: Jalview; Species abbreviations and conservation histogram as in Fig. 1A. (**B**) Electrostatic charge distribution on AlphaFold2 monomer GTSF-1 structures (-5kBT in red to +5kBT in blue). *M. musculus* GTSF1 exhibits a positively charged ridge on ZnF1 as reported (Ipsaro et al, 2021). *C. elegans* and *C. briggsae* display positive regions between ZnF1 and ZnF2, with reduced effect in *P. pacificus*. (**C**) Domain map of *C. elegans* GTSF-1 mutants showing full-length (FL) or C-terminal truncation (ΔC) with 3×FLAG epitope tag positions. E92 and L169: end of N-terminal and C-terminal regions, respectively; E100: C-terminal truncation position. (**D**) Western blot detecting GTSF-1 proteins with αFLAG and αCelGTSF-1 antibodies (100 gravid adults handpicked and boiled). αCelGTSF-1 targets the C-terminal region of *C. elegans* GTSF-1, detecting the wild-type protein and, to some extent, FL GTSF-1. Arrows: non-specific bands. Circles: Positions of wild-type, FL or ΔC GTSF-1 with expected sizes. TBA-1: tubulin loading control. The experiment was tested through two biological replicates. (**E**) Brood sizes of GTSF-1 mutant animals at 20 °C and 25 °C (*n* = 15–25) as box plots. Box plots show the median, 25th–75th percentiles (box), and whiskers extending to the most extreme value within 1.5× the interquartile range, with outliers shown as individual points. *P* values: Wilcoxon rank-sum test with BH correction. (**F**) Western blot detecting 3×FLAG::RRF-3 in wild-type or GTSF-1 mutant animals (100 gravid adults handpicked and boiled). Filled or empty circles indicate the presence or absence of the indicated protein. Arrow: non-specific band or 3×FLAG::RRF-3 with expected size. TBA-1: tubulin loading control. (**G**) Co-IP/MS using anti-FLAG magnetic beads on *C. elegans* embryo lysates. *X* axis: log₂ Fold Change; *Y* axis: Adjusted *P* values (*q* value or t(SAM) statistic (Tusher et al, 2001)). Genotype abbreviations: FL= *gtsf-1(fl)::3×flag*; *3×flag::rrf-3; rrf-3(pk1426)*, ΔC= *gtsf-1(ΔC)::3×flag*; *3×flag::rrf-3*; *rrf-3(pk1426)*. Dotted lines: log₂ Fold Change ±2. ERI complex components highlighted. *n* = 4 biological replicates per sample. Source data are available online for this figure.

Coimmunoprecipitation studies from mouse and flies have demonstrated that the central portion of Gtsf1 contains aromatic residues that are essential for Piwi binding (Dönertas et al, 2013; Yoshimura et al, 2018). Although the Piwi binding aromatic residues are conserved in many animal GTSF1 proteins, our set of nematode orthologs lacks them entirely (Fig. 3A). In addition, a relatively conserved positively charged surface on the first zinc finger of mouse GTSF1 has been implicated in RNA binding and shown to be essential to activate PIWI (Arif et al, 2022; Ipsaro et al, 2021). This positively charged region is also absent from our set of nematodes (Fig. 3B), suggesting that RNA binding may be lost in nematode GTSF-1. This is consistent with a previous report in which in vitro iCLIP on purified *C. elegans* GTSF-1 did not precipitate RNA beyond background levels (Almeida et al, 2018).

We sought to determine how nematode GTSF-1 binds to RRF-3. First, we tested whether the C-terminal region of *C. elegans* GTSF-1 contributes to RRF-3 binding and ERI complex assembly. We generated a *gtsf-1* allele lacking this domain, leaving only the N-terminal region fused to a 3×FLAG tag (Fig. 3C). For brevity, we refer to this truncated GTSF-1 as ‘ΔC’. As a control, we also tagged full-length GTSF-1 with 3×FLAG. Western Blot analysis showed that wild-type GTSF-1 migrated slower than expected, as observed previously (Almeida et al, 2018) (Fig. 3D). We detected the truncated GTSF-1 protein on a western blot, though at lower levels than full-length protein (Fig. 3D). Interestingly, animals expressing GTSF-1 ΔC showed reduced fertility at 20 °C (though they were more fertile than gtsf-1 null mutants) and were near sterile at 25 °C (Fig. 3E).

We checked whether GTSF-1 truncation affects RRF-3 expression by crossing GTSF-1 ΔC animals to a strain carrying a single-copy 3×*flag*::*rrf-3* transgene in a *rrf-3* deletion *(rrf-3*(*pk1426*)) background. Western blotting showed that RRF-3 protein remained detectable after the GTSF-1 C-terminus was deleted (Fig. 3F). Based on these findings, we immunoprecipitated embryonic RRF-3 using αFLAG beads from animals carrying *3×flag::rrf-3; rrf-3(pk1426)* and expressing either 3×FLAG fused full-length or truncated GTSF-1. In the presence of full-length GTSF-1, we enriched all known ERI complex proteins (Fig. 3G), as expected. The enrichments of these proteins were significantly lower when GTSF-1 ΔC was expressed, but most of the factors were still detected (Fig. 3G). Notably, ERI-9 and ERI-3 peptides were not detected in GTSF-1 ΔC samples, suggesting these interactions within the ERIC may be particularly destabilized. Overall, these findings demonstrate that the C-terminal regions of CelGTSF-1 are not essential for RRF-3 binding, but they do appear to stabilize the assembled ERI complex, with noteworthy importance at critical temperatures. This is consistent with a previous report where recombinant GTSF-1 comprising only the N-terminal region was sufficient to precipitate RRF-3 from embryonic extracts (Almeida et al, 2018).

### Nematode GTSF-1 binds a conserved folded domain of RRF-3, the GID

**Figure 4:**
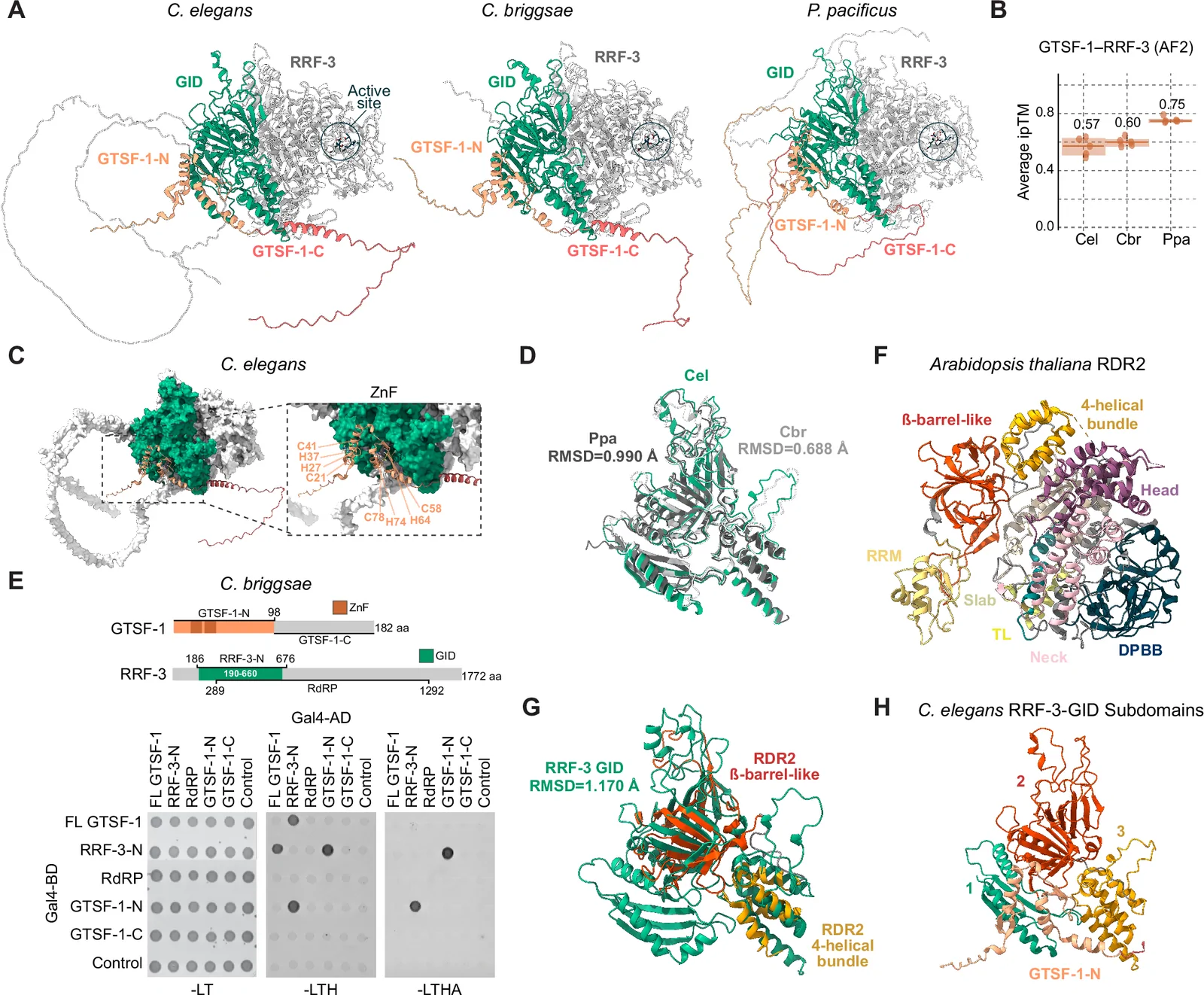
GTSF-1 N-terminus directly interacts with an N-terminal region in RRF-3. (**A**) AlphaFold2 multimer models of GTSF-1 bound to RRF-3. GTSF-1-N: GTSF-1 N-terminal region; GTSF-1-C: GTSF-1 C-terminal region; GID: GTSF-1 Interacting Domain. Active site: RdRP catalytic site (DLDGDE). (**B**) Average interface predicted template modeling scores (ipTM) from AF2 models of GTSF-1–RRF-3 complexes (*n* = 5). Species: Cel, *C. elegans*; Cbr, *C. briggsae*; Ppa, *P. pacificus*. Top-ranked prediction displayed in panel A. (**C**) Surface model of the *C. elegans* GTSF-1–RRF-3 complex. The inset image shows the positioning of CHHC zinc fingers (ZnF) residues over the GID of RRF-3. (**D**) Superimposition of RRF-3 GID structures from (**A**). *C. elegans* RRF-3 GID (residues 190–660) was used as the reference for structural alignment, and Cα RMSD values were calculated. RMSD values were 0.990 Å for *P. pacificus* (residues 53–497) and 0.688 Å for *C. briggsae* (residues 197–666). (**E**) Yeast Two-Hybrid assays between *C. briggsae* GTSF-1 and RRF-3 regions. Top: domain architecture highlighting the tested regions. Plates show selection (− LT: −Leu, −Trp), low stringency (− LTH: −Leu, −Trp, −His), and high stringency (− LTHA: −Leu, −Trp, −His, −Ade) conditions. Control: Gal4 activation domain (AD) or DNA-binding domain (BD). Experiment tested through two biological replicates. (**F**) EM structure of *Arabidopsis thaliana* RNA-dependent RNA polymerase 2 (RDR2; PDB: 7RQS; 3.57 Å resolution). Domains are highlighted as defined in (Fukudome et al, 2021). (**G**) *C. elegans* RRF-3 GID superimposed on residues 101–360 of *A. thaliana* RDR2 (AtRDR2). Cα RMSD = 1.170 Å. (**H**) *C. elegans* GTSF-1–RRF-3 AF2 complex showing only RRF-3 GID with GTSF-1-N. The GID subdomains 1 to 3 were defined with respect to (**G**). Source data are available online for this figure.

**Figure EV3:**
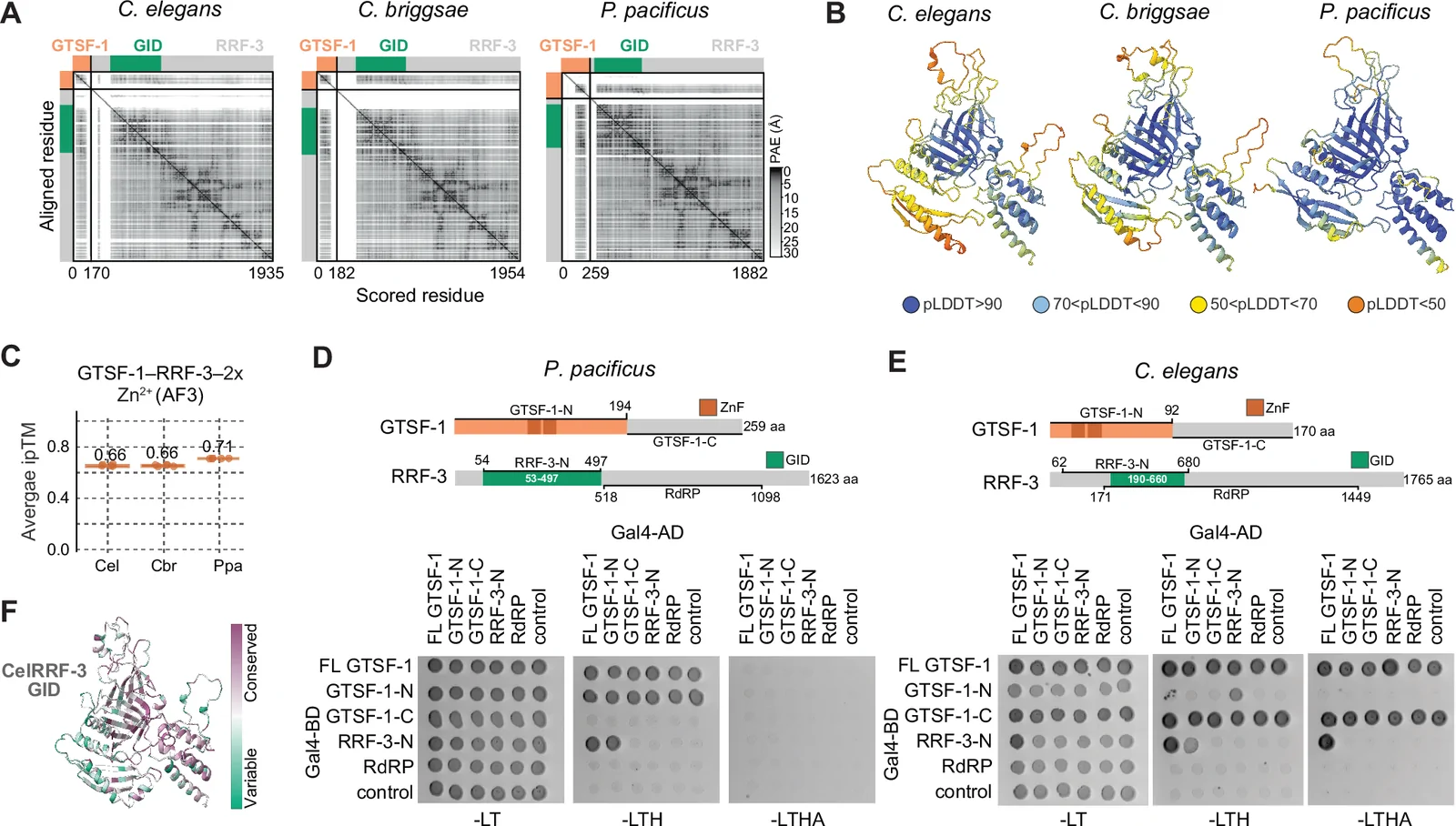
The GTSF-1–RRF-3-GID interaction is conserved in *C. elegans*, *C. briggsae*, and *P. pacificus.* (**A**) PAE plots for GTSF-1–RRF-3 AF2 complexes displayed in Fig. 4A. (**B**) RRF-3-GID regions colored by pLDDT (0–100). (**C**) Average ipTM from AlphaFold3 Multimer models of GTSF-1–RRF-3–2xZn2+ complexes (*n* = 5). (**D**, **E**) Yeast Two-Hybrid assays between GTSF-1 and RRF-3 regions. Plates show selection (-LT: -Leu, -Trp), low stringency (-LTH: -Leu, -Trp, -His), and high stringency (-LTHA: -Leu, -Trp, -His, -Ade) conditions. Control: Gal4 activation domain (AD) or DNA-binding domain (BD). Experiment tested through two biological replicates. (**F**) ConSurf conservation scores mapped onto *C. elegans* RRF-3-GID. Scores were calculated from an MSA of 11 RRF-3 orthologs (*C. elegans, C. briggsae, C. remanei, C. latens, C. nigoni, C. brenneri, C. japonica, C. sp.36, C. angaria, C. bovis*, and *P. pacificus*). Scores range from 1 to 9, colored from turquoise (score 1; highly variable) to maroon (score 9; highly conserved).

Next, we aimed to identify the region of RRF-3 that interacts with GTSF-1. To achieve this, we generated AlphaFold2 (AF2) Multimer models of GTSF-1 and RRF-3. For all three species, the models predicted that the CHHC-containing α-helices within the GTSF-1 N-terminal region contact a region in the N-terminus of RRF-3, distal to the RdRP catalytic site (Figs. 4A–C and  EV3A). This contrasts sharply with other metazoan GTSF1/Gtsf1 orthologs, which engage the catalytic PIWI domain of PIWI Argonautes (Portell-Montserrat et al, 2025; De et al, 2025). We refer to this region of RRF-3 as the GTSF-1-interacting domain or GID (Fig EV3B). The structure of GID remained nearly identical in *C. briggsae* and *P. pacificus* RRF-3, with 69%/30% identity and 79%/42% similarity to *C. elegans*, respectively, suggesting deep conservation of this domain (Fig. 4D). We also tested this interaction with AlphaFold3 (AF3) Multimer, which additionally allowed us to provide two Zinc ions along with GTSF-1 and RRF-3. AF3-Multimer similarly predicted a high-confidence interaction of the GTSF-1–RRF-3 complex (Fig. EV2C).

To validate the predicted structures, we tested the pairwise interaction of regions of GTSF-1 and RRF-3 using the Yeast Two-Hybrid (Y2H) assay. For GTSF-1, we tested the full-length protein, the N-terminal region which contains both zinc fingers, and the C-terminal region (Figs. 4E and  EV3D,E). The N-terminal region for PpaGTSF-1 also included the 100 amino acid IDR. For RRF-3, we designed fragments spanning the GID at the N-terminal region and the InterPro/PFAM annotated RdRP domain. The annotated RdRP domain for *C. elegans* and *C. briggsae* RRF-3 partially overlapped with the region that was selected for the N-terminal region. We detected a robust interaction between GTSF-1 and the N-terminal region of RRF-3 for *C. briggsae* (Fig. 4E). This interaction was particularly strong for the N-terminal construct of GTSF-1, which showed positive interaction with RRF-3 even under stringent selection. For *C. elegans* and *P. pacificus*, GTSF-1 constructs fused to Gal4 DNA-binding domain were self-activating (Fig. EV3D,E). However, when expressed with the Gal4 activation domain, both full-length and N-terminal GTSF-1 showed positive interactions with the N-terminal region of RRF-3. For all three species, we did not detect any interaction with the RdRP construct. Altogether, our Y2H and structural analyses show that the GID is a conserved domain in the RRF-3 N-terminus that directly binds the zinc finger region of GTSF-1 in nematodes.

### GID subdomains share homology with eukaryotic RdRP N-terminal domains

To place RRF-3 GID in a broader evolutionary context, we compared AF2-predicted RRF-3 structures to experimentally determined RdRP structures. Eukaryotic “cellular” RdRPs form a distinct family from viral RdRPs (Zong et al, 2009), and among these, the structures of plant RdRPs, such as *Arabidopsis* (AtRDR2) (Huang et al, 2021; Fukudome et al, 2021) and *Zea mais* (ZmRDR2) (Du et al, 2022) have been experimentally determined. Given the high homology between these plant RdRPs, we only focused on AtRDR2 for our comparative analysis with AF2-predicted RRF-3. The AtRDR2 structure comprises an N-terminal region containing an RRM-like fold, a β-barrel-like domain, and a four-helical bundle domain, followed by an active site cleft composed of multiple conserved domains including the slab, DPBB1 and DPBB2 (double-ψ β-barrel) domains, connector helices (neck 1, 2, and 3), bridge helix, trigger loop, and head domain (Fukudome et al, 2021; Huang et al, 2021) (Fig. 4F). Structural alignment revealed that the RRF-3 GID superimposes well with AtRDR2 and can be divided into three subdomains (Fig. 4G,H). Subdomains 2 and 3 are structurally homologous to the N-terminal regions of AtRDR2 that precede the DPBB core. Subdomain 2 corresponds to the β-barrel-like fold, also referred to as the “PH” domain in ZmRDR2 (Du et al, 2022) or the “Guide” domain in an older annotation of AtRDR2 (Huang et al, 2021). Subdomain 3 aligns with a four-helix bundle that is annotated as part of the “Slab” domain in both plant RdRPs (Fig. 4G,H). In contrast, subdomain 1 showed no clear structural homology to any AtRDR2 domain (Fig. 4H). We also calculated per-residue conservation scores using ConSurf from a multiple sequence alignment of 11 nematode RRF-3 orthologs (Fig. EV2F). Residues comprising subdomain 1 appeared to be less conserved overall than those of subdomains 2 and 3. These findings suggest that subdomains 2 and 3 of the RRF-3 GID correspond to deeply conserved ancestral RdRP structural elements, whereas subdomain 1 appears to be less conserved and absent from plant RdRPs.

### Critical residues mediating the GTSF-1–RRF-3 interaction

**Figure 5:**
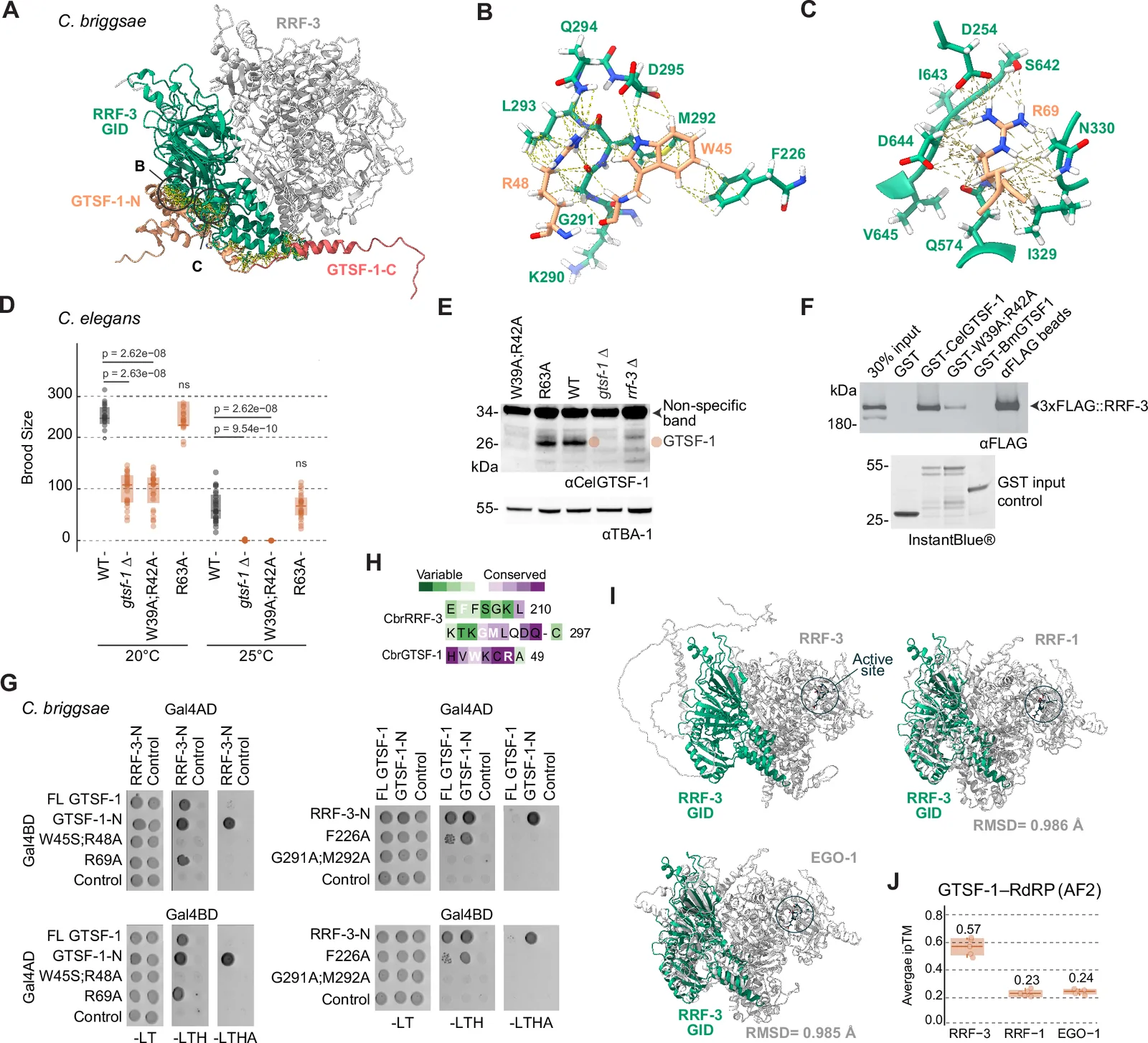
GTSF-1-RRF-3 interface residues are critical for interaction and fertility. (**A**) AF2 model of the *C. briggsae* GTSF-1–RRF-3 complex, highlighting the protein–protein interaction interface. Interchain contacts under 3 Å displayed as yellow dashes. Circles indicate the regions magnified in (**B**, **C**). (**B**, **C**) Close-up of the interface regions indicated in (**A**). Key predicted contact residues on GTSF-1-N and RRF-3 GID are displayed as sticks. Yellow dashes indicate predicted interchain contacts below 3.5 Å. (**D**) Brood size of *C. elegans gtsf-1* mutants as box plots (*n* = 15–25). Box plots show the median, 25th–75th percentiles (box), and whiskers extending to the most extreme value within 1.5× the interquartile range, with outliers shown as individual points. *P* values: Wilcoxon rank-sum test with BH correction. ns: non-significant compared to wild-type (WT). (**E**) Western blot on *C. elegans* GTSF-1 mutant animals compared to wild-type, *gtsf-1* deletion *(xf251)* and *rrf-3* deletion (*pk1426*) animals (100 gravid adults handpicked and boiled). TBA-1: tubulin loading control. Experiment tested through two biological replicates. (**F**) Western blot displaying levels of 3×FLAG::RRF-3 precipitated from *C. elegans* embryo lysates using GST or GST-GTSF-1 proteins. Input: 30% of embryo lysate used per pulldown. αFLAG beads: Positive control; Pulldown using αFLAG magnetic beads. GST input control: 10% of GST or GST-GTSF-1 proteins used for pulldown, visualized using InstantBlue®. Experiment tested through two biological replicates. (**G**) Yeast Two-Hybrid assays between *C. briggsae* GTSF-1 and RRF-3 with point mutations in the N-terminal constructs of both proteins. Constructs assayed under selection (-LT: -Leu, -Trp), low stringency (-LTH: -Leu, -Trp, -His), and high stringency (-LTHA: -Leu, -Trp, -His, -Ade) conditions. Control: Gal4AD or Gal4BD empty vectors serving as negative controls. Experiment tested through two biological replicates. (**H**) Selected residues of *C. briggsae* GTSF-1 and RRF-3 colored by ConSurf score. Score was calculated with a MSA of 26 GTSF-1 orthologs (*C. elegans, C. briggsae, C. inopinata, P. pacificus, C. brenneri, C. castelli, C. doughertyi, C. japonica, C. kamaaina, C. nouraguensis, C. plicata, C. sp21, C. sp26, C. sp28, C. sp29, C. sp31, C. sp38, C. sp39, C. sp40, C. tropicalis, C. virilis, C. nigoni, Toxocara canis, Brugia malayi, Wuchereria bancrofti, Brugia pahangi)* and 11 RRF-3 orthologs (*C. elegans, C. briggsae, C. remanei, C. latens, C. nigoni, C. brenneri, C. japonica, C. sp.36, C. angaria, C. bovis and P. pacificus*). White bold-face residues were tested experimentally. (**I**) AF2 Monomer structures of *C. elegans* RdRPs. RRF-3 GID is superimposed on RRF-1 and EGO-1. Active site: DLDGDE catalytic residues of RdRPs. RMSD between RRF-3 GID (residues 190–660) and RRF-1 = 0.986 Å and EGO-1 = 0.985 Å. (**J**) Average ipTM scores from five AF2 predictions of GTSF-1–RdRP complexes. Top-ranked prediction displayed in panel I. Source data are available online for this figure.

**Figure EV4:**
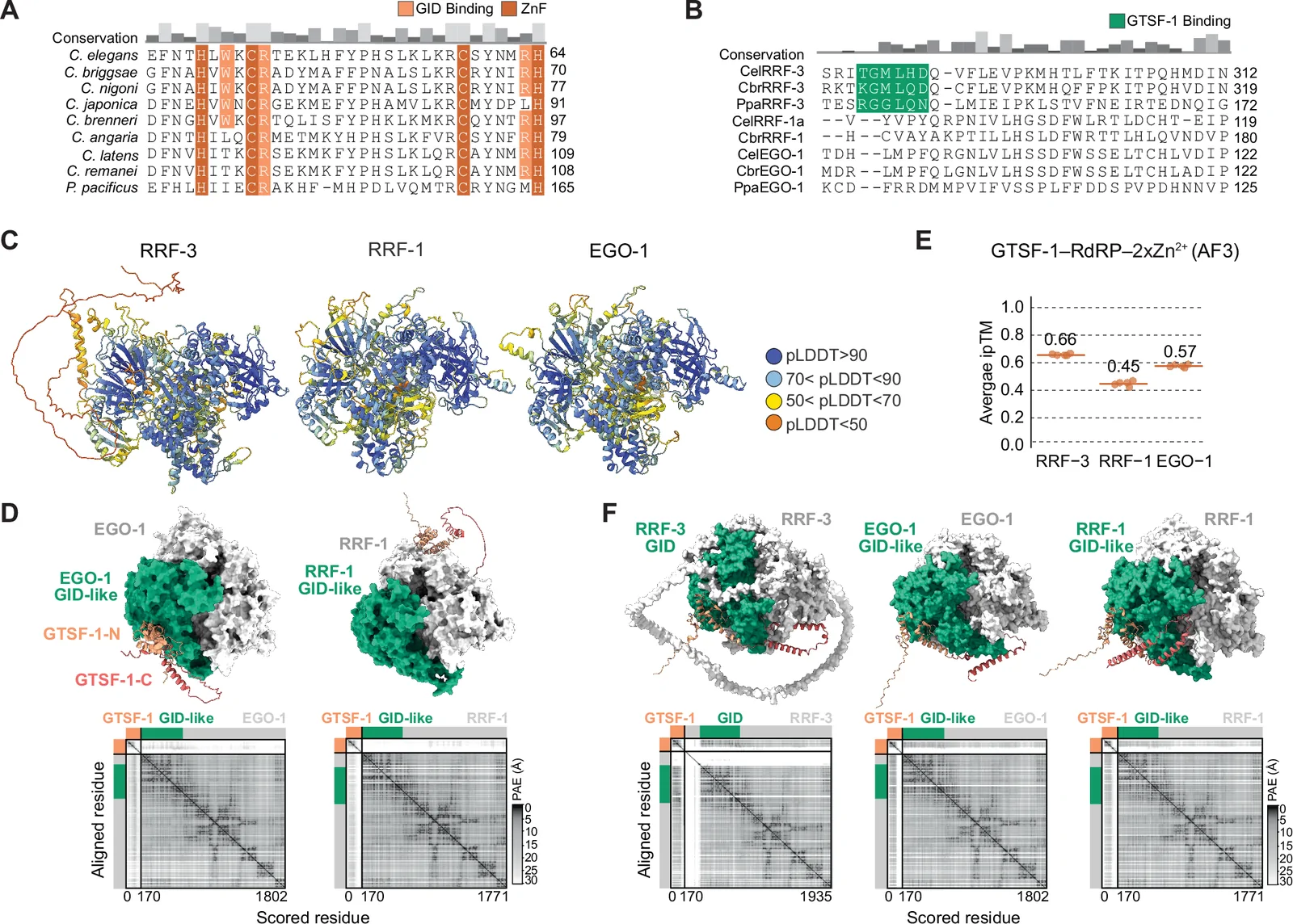
AF3 predicts a conserved interaction mode of GTSF-1 with RRF-1 and EGO-1. (**A**) Clustal Omega MSA of nematode GTSF-1 orthologs. Highlighted residues part of CHHC Zinc fingers (ZnF) and experimentally tested to bind RRF-3 GID. (**B**) Clustal Omega MSA of RdRP sequences. Species: Cel, *C. elegans*; Cbr, *C. briggsae*; Ppa, *P. pacificus*. Residues within the RRF-3 GID that form the GTSF-1 binding interface are highlighted. (**C**) AF2 monomer structures of *C. elegans* RdRPs colored by pLDDT confidence score. (**D**) Top-ranked AF2 multimer predictions of *C. elegans* GTSF-1 in complex with RRF-1 or EGO-1, displayed as surface models alongside their respective PAE plots. GID-like denotes the region in RRF-1 and EGO-1 homologous to the RRF-3 GID. GTSF-1-N and C are N-terminal and C-terminal regions of GTSF-1 respectively. (**E**, **F**) AF3 multimer predictions of *C. elegans* GTSF-1–RdRP–2×Zn2+complexes. (**E**) Average ipTM scores from *n* = 5 predictions. (**F**) Surface models of the top-ranked predictions alongside their respective PAE plots. Source data are available online for this figure.

Having defined the overall architecture and evolutionary context of the RRF-3 GID, we next sought to identify the specific residues that mediate its interaction with GTSF-1. We examined the AF2-predicted complexes of GTSF-1 and RRF-3 (Fig. 5A). For the complexes in *C. briggsae*, contacts between the two proteins were concentrated on the N-terminal region of GTSF-1. We focused on two major contact regions adjacent to the zinc finger motifs of GTSF-1, where the aromatic residue W45 and the positively charged residues R48 and R69 project into complementary surfaces on the RRF-3 GID (Fig. 5B,C). Furthermore, a multiple sequence alignment of *Caenorhabditis* GTSF-1 orthologs revealed strong conservation at W45 and R428 and moderate conservation at R69 (Fig. EV4A), which motivated functional testing.

Using CRISPR/Cas9, we introduced point mutations in the corresponding residues on *C. elegans* GTSF-1 (W39,R42,R63). We generated strains carrying either W39A;R42A or R63A and assessed their fertility. *C. elegans* GTSF-1 W39A;R42A mutants produced reduced brood size at 20 °C and became sterile at 25 °C (Fig. 5D). In contrast, the brood-size of R63A mutants was comparable to wild-type animals at both temperatures (Fig. 5D). Interestingly, we could not detect W39A;R42A GTSF-1 protein by Western blot, whereas R63A maintained wild-type protein levels (Fig. 5E). Mutating W39 and R42 thus appear to destabilize GTSF-1. Given the close physical association between GTSF-1 and RRF-3, we reasoned that the observed degradation of GTSF-1 might be due to an inability to bind RRF-3. Consistent with this hypothesis, we did not detect wild-type GTSF-1 in RRF-3 deletion animals (Fig. 5E).

To directly test whether W39 and R42 are required for RRF-3 binding independently of cellular stability effects, we performed GST pulldown assays using recombinantly expressed GST-fused GTSF-1 proteins. Both wild-type and W39A;R42A GTSF-1 were expressed at comparable levels in *E. coli*, confirming that the mutations do not intrinsically affect protein stability (Fig. 5F, bottom). However, upon incubation with *C. elegans* embryo lysates, wild-type GTSF-1 pulled down 3×FLAG::RRF-3 more efficiently than the W39A;R42A variant (Fig. 5F, top).

We sought to validate these findings in an independent system as well as extend the analysis to the RRF-3 side of the interface. For this, we turned to yeast two-hybrid (Y2H) assays using *C. briggsae* proteins. Our results were consistent with the *C. elegans* data such that CbrGTSF-1(W45S;R48A) failed to interact with CbrRRF-3, whereas CbrGTSF-1(R69A) retained binding (Fig. 5G). Based on the AF2 predicted structures, we tested F226, G291, and M292 from *C. briggsae* RRF-3 GID, as these contact GTSF-1 W45 and R48. ConSurf analysis of RRF-3 orthologs indicated that G291 and M292 are more highly conserved than F226 (Fig. 5H). When we tested these residues in Y2H, the G291A/M292A double mutation abolished the interaction between RRF-3 and GTSF-1 (Fig. 5G). F226A also showed a weak but noticeable binding defect with GTSF-1.

Together, these results confirm that the GTSF-1–RRF-3 interface predicted by AF2 is functionally required for their interaction and establish that GTSF-1 stability is coupled to its binding to RRF-3.

### RRF-1 and EGO-1 possess GID-like domains but are not predicted to interact with GTSF-1 by AF2-multimer

*C. elegans* also encodes the RdRPs RRF-1 and EGO-1, which act downstream of RRF-3 but function independently of DCR-1 and the ERI complex. We next asked whether RRF-1 and EGO-1 contain a structural feature similar to the GID. AF2 monomer predictions revealed that the N-terminal regions of RRF-1 and EGO-1 adopt a fold that superimposes well with the RRF-3 GID (Fig. 5I). However, AF2-Multimer failed to yield a high-confidence prediction for the GTSF-1–RRF-1 and GTSF-1–EGO-1 complex (Figs. 5J and  EV4D). Interestingly, AF3 predicted GTSF-1 binding to all three RdRPs with comparable confidence scores (Fig. EV4E), positioning GTSF-1 at the putative GID region in each case (Fig. EV4F). AF3 was therefore less able to recapitulate the in vivo interaction specificity of GTSF-1 towards RRF-3.

This discrepancy between the two prediction methods likely reflects differences in how they use coevolutionary information. AF2-Multimer relies on paired multiple sequence alignment (MSA) data that encode interchain coevolution and performs best when a clear evolutionary signal supports a specific protein–protein interface (Evans et al, 2021). When that signal is weak or absent, as may be the case for the GTSF-1–RRF-1 and GTSF-1–EGO-1 pairs, interface prediction quality deteriorates, which is reflected in lower confidence scores. By contrast, AF3 relies less on deep MSA processing and instead uses single and pair representations to generate coordinates via a diffusion model (Abramson et al, 2024). Because RRF-1, RRF-3, and EGO-1 share a highly similar structural fold and partially conserved surface features at the GID, AF3 may rely predominantly on these structural similarities, predicting GTSF-1 binding to all three RdRPs. However, these explanations remain untested and will require further validation.

Together, these results suggest that all three *C. elegans* RdRPs share a GID-like N-terminal architecture. However, the selective interaction of GTSF-1 with RRF-3 in vivo is best captured by AF2-Multimer. Therefore, we decided to perform further analysis using AF2-Multimer.

### Conservation patterns of GTSF-1, PIWI, and RdRP across the phylum Nematoda reveal clade-specific losses

We next sought to trace the evolutionary history of this interaction and determine whether nematode GTSF-1 originally may have partnered with PIWI or RdRP proteins. The phylum Nematoda comprises three classes: Dorylaimia (Clade I), Enoplia (Clade II), and Chromadoria (Clade C) (Blaxter et al, 1998). Within Chromadoria, the order Rhabditida includes three subclasses: Spirurina (Clade III), Tylenchina (Clade IV), and Rhabditina (Clade V) (Blaxter et al, 1998). Clade V contains the genera *Caenorhabditis and Pristionchus*. We searched for orthologs of *C. elegans* PRG-1, RRF-3, RRF-1/EGO-1, and GTSF-1 in species representing all major nematode clades. Our set included species from Enoplia and early-branching Chromadorea. We also included four species from the phylum Nematomorpha, as they represent the sister group to Nematoda.

**Figure 6:**
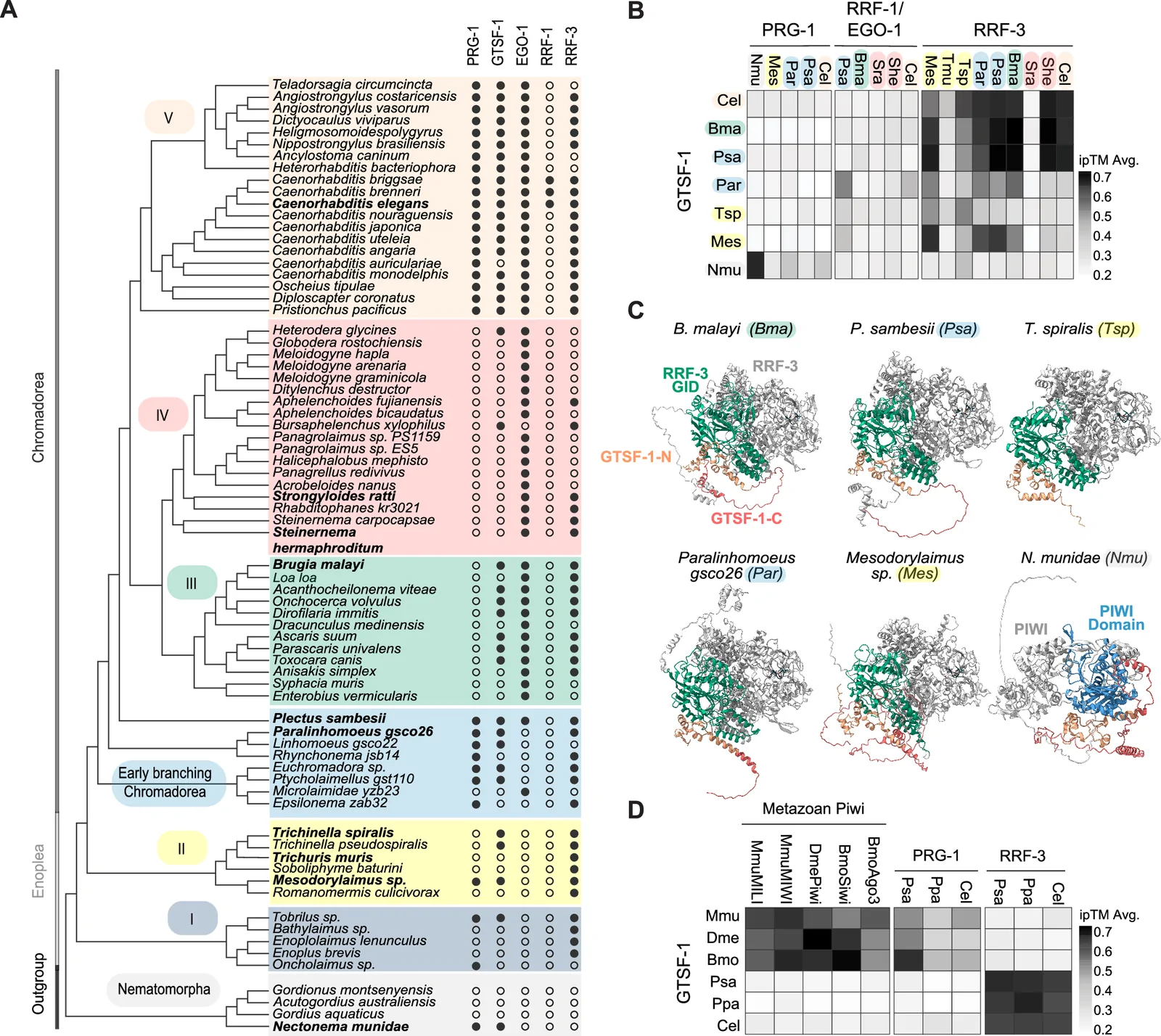
GTSF-1–RRF-3 interaction is conserved across nematodes. (**A**) Conservation of selected proteins across phylum Nematoda and Nematomorpha. Species are color-coded by Clades I-V and Early-branching Chromadorea. Filled and empty circles indicate the presence or absence of the indicated orthologous protein. Bold-faced species were selected for the AlphaFold2 Multimer pairwise prediction screen. (**B**) Heatmap of AlphaFold2 Multimer pairwise interactions between PIWI, RRF-1/EGO-1, and RRF-3 orthologs and GTSF-1 proteins. Species: She, *S. hermaphroditum*; Sra, *S. ratti*; Bma, *B. malayi*; Psa, *P. sambesi*; Par, *Paralinhomoeus gsco26*; Tsp, *T. spiralis*; Tmu, *T. muris*; Mes, *Mesodorylaimus sp.;* Nmu, *N. munidae*. (**C**) AF2 structures of cognate RRF-3-GTSF-1 or PIWI-GTSF-1 complexes from (**B**). (**D**) Heatmap of AF2 Multimer pairwise interactions between PIWI, PRG-1, and RRF-3 orthologs and GTSF-1/GTSF1/Gtsf1 proteins. Species additional to panel B: Mmu: *M. musculus*, Dme: *D. melanogaster*, Bmo: *B*. mori. Source data are available online for this figure.

Through a reciprocal BLAST approach, we identified orthologs of PIWI, RdRP, and GTSF-1 in genomes of 80 nematode species (Fig. 6A). Our results were consistent with previous reports: *C. elegans* PRG-1 orthologs were absent in Clades III and IV, as well as in several Clade I and II species, indicating repeated loss of the piRNA pathway across the nematode phylum (Sarkies et al, 2015). RRF-3-like RdRPs are found across all clades, while RRF-1/EGO-1 proteins are mostly found within Clades III-V. Notably, we found both RRF-1 and EGO-1 only in *C. briggsae*, *C. brenneri*, and *C. elegans*, which suggests these gene paralogs likely arose after a recent gene duplication in *Caenorhabditis*. These results indicate that RRF-1/EGO-1 most likely lost the ability to bind GTSF-1, rather than that RRF-3 uniquely gained the property to bind GTSF-1.

GTSF-1 showed patchy conservation across the phylogeny, though we detected it in at least one species from each clade (Fig. 6A). Conservation was strongest within Clade V, where we found GTSF-1 in 19 of 20 species tested. In contrast, Clade IV showed the most striking loss: GTSF-1 was absent from 16 of 18 species, 11 of which also lacked RRF-3. Within the entire set, we identified 11 species where RRF-3 was present without GTSF-1. We also found 5 species where GTSF-1 was detected, while RRF-3 was not. These phylogenetic patterns show that GTSF-1 and RRF-3 orthologs are present broadly across Nematoda, where they are found in most species, and that they tend to co-exist. However, gene loss seems to be frequent, indicating fast-changing small RNA pathway dynamics amongst nematodes. Finally, our set of Nematomorphs was largely devoid of PIWI, GTSF-1, and RdRPs. However, in one species, *Nectonema munidae*, we did find an ortholog of both PIWI and GTSF-1.

### Structural modeling reveals the specificity of GTSF-1 interactions

We extracted high-confidence sequences from key species and used AF2-Multimer to predict pairwise interactions between GTSF-1 proteins and either RdRPs or PIWI proteins. We selected 10 species representing key phylogenetic positions (Fig. 6A, bold face). *Trichinella spiralis* (Clade I) and *Brugia malayi* (Clade III) both contained GTSF-1 and RRF-3 but lacked PRG-1. Two Clade IV species (*Strongyloides ratti* and *Steinernema hermaphroditum*) and the Clade I species *Trichuris muris* encoded RRF-3 but no GTSF-1. In addition, three species were of particular interest: the Clade I species *Mesodorylaimus sp*. and the early-branching Chromadorea species *Paralinhomoeus gsco26 and Plectus sambesi*. All three encoded PRG-1, RRF-3, and GTSF-1, allowing us to test both potential interactions. Finally, *N. munidae* GTSF-1 and PRG-1 sequences enabled us to predict GTSF-1’s binding partner in the phylum most closely related to the Nematoda.

**Figure EV5:**
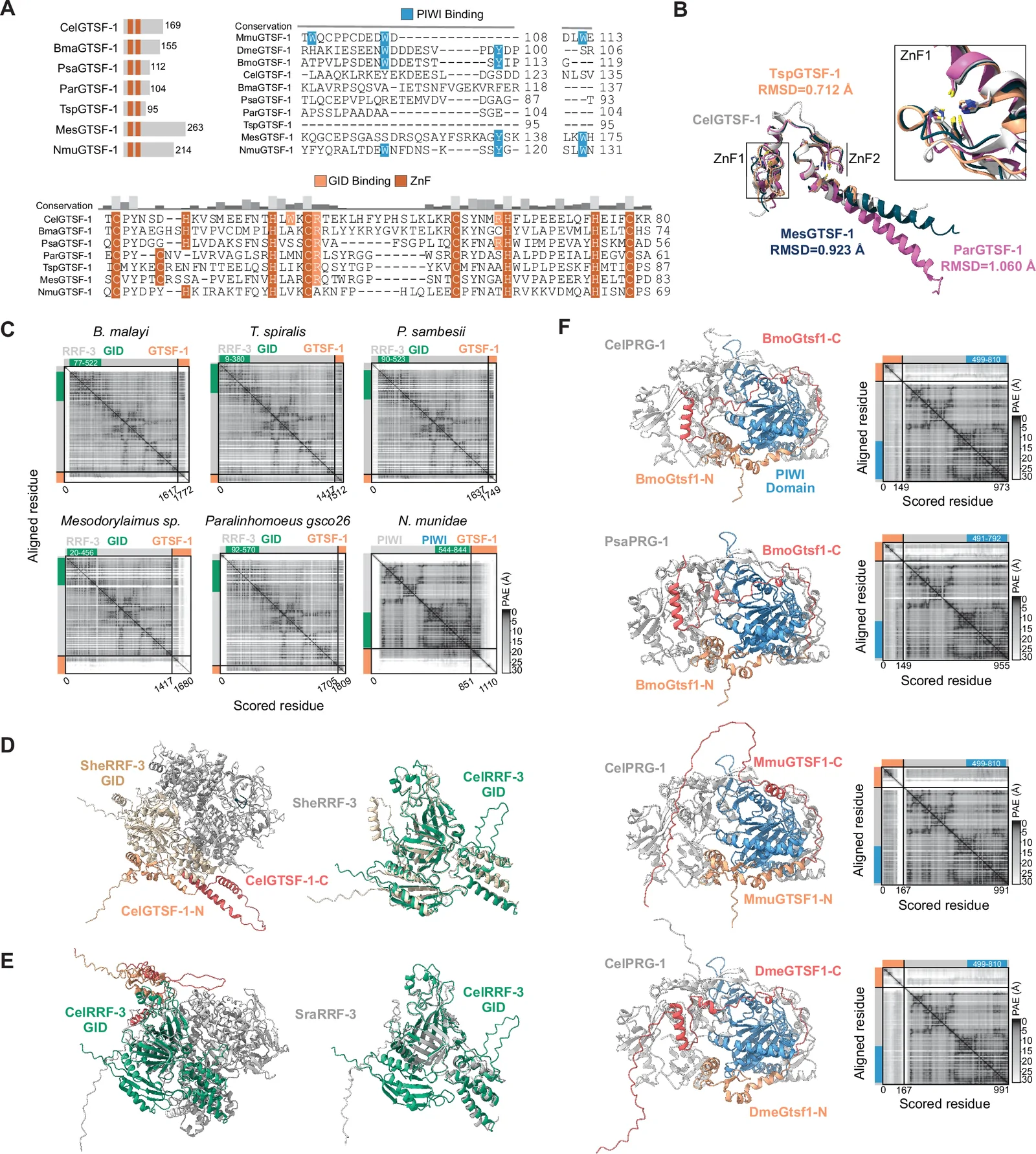
GTSF-1 structure and interaction mode with RRF-3 are conserved across the nematode phylum. (**A**) Left: Schematic of GTSF-1 lengths from selected nematodes. Right, Bottom: MSA of GTSF-1 regions. Orange boxes: CHHC zinc-finger residues and residues tested for GID binding. Blue boxes: Residues tested for PIWI binding in *D. melanogaster* (Dönertas et al, 2013) and *M. musculus* (Yoshimura et al, 2018), Species: Cel, *C. elegans*; Bma, *B. malayi*; Psa, *P. sambesi*; Par, *Paralinhomoeus gsco26*; Tsp, *T. spiralis*; Mes, *Mesodorylaimus sp*.; Nmu, *N. munidae*. (**B**) Superimposed structures of GTSF-1 from *C. elegans* (Cel), *T. spiralis* (Tsp), *Mesodorylaimus sp*. (Mes), and *Paralinhomoeus gsco26* (Par) panel zoom on CCHC type zinc finger 1 (ZnF1). *C. elegans* GTSF-1 (residues 17–92) was used as the reference, and Cα RMSD values were calculated. RMSD: 0.712 Å for *T. spiralis* (residues 1–95), 0.923 Å *for Mesodorylaimus sp*. (residues 23–97), and 1.060 Å *Paralinhomoeus gsco26* (residues 1–76). (**C**) PAE plots of AlphaFold2 structures from Fig. 6C. (**D**) AF2 multimer of *C. elegans* GTSF-1 with *S. hermaphroditum* (She) RRF-3, Superimposed with *C. elegans* RRF-3 GID. Right: Superimposed structures of *C. elegans* RRF-3 GID with *S. hermaphroditum* residues 1-461, Cα RMSD = 0.920. (**E**) AF2 multimer of *C. elegans* GTSF-1 with *S. ratti* (Sra) RRF-3, superimposed with *C. elegans* RRF-3 GID. Right: Superimposed structures of *C. elegans* RRF-3 GID with *S. ratti* residues 1–364, Cα RMSD = 0.845. (**F**) AF2 structures of top-ranked PRG-1-GTSF1/Gtsf1 complexes from Fig. 6D alongside respective PAE plots.

The GTSF-1 sequences varied considerably in length: *T. spiralis* GTSF-1 comprised only 95 amino acids restricted to the zinc finger region, whereas *Mesodorylaimus* GTSF-1 extended to 263 amino acids with a long C-terminal (Fig. EV5A). Despite being seemingly truncated at the C-termini, the shorter GTSF-1 sequences appear to be complete, with fully intact Zinc-binding domains. We note that, except for *Mesodorylaimus*, the closely related species *T. spiralis*, *Paralinhomoeus gsco26*, and *P. sambesi* all three have shorter C-terminal tails, possibly reflecting a shared, but unresolved requirement for shortening of this region. Interestingly, *T. spiralis*, *Mesodorylaimus sp*., and *Paralinhomoeus* GTSF-1 proteins contained CCHC motifs instead of the canonical CHHC motif, and AF2 predicted these to fold into functional zinc fingers (Fig. EV5B).

We detected high-confidence interactions between cognate GTSF-1 and RRF-3 proteins from *B. malayi, P. sambesii*, and *Mesodorylaimus sp*., with lower but still favorable scores for *T. spiralis* and *Paralinhomoeus* (Fig. 6B). To test the specificity of these predicted interactions, we compared closely related RRF-3 orthologs from *T. spiralis*, which does have a GTSF-1 homolog, and *T. muris*, which does not encode GTSF-1. *T. spiralis* RRF-3 was predicted to bind GTSF-1 homologs from various nematodes, also from other clades, such as *C. elegans*. In contrast, *T. muris* RRF-3 showed no favorable binding with any of the GTSF1 proteins from our dataset, nor with those from the closely related *T. spiralis*. This result again demonstrated that AlphaFold2 homology modeling predicted differences in interaction specificity among closely related orthologs. All favorable GTSF-1-RRF-3 complexes featured a GID in the N-terminus of RRF-3 that engaged the GTSF-1 NTD, mirroring the architecture we observed in *C. elegans* (Fig. 6C).

Within Clade IV, where most species lack GTSF-1, the two species we tested displayed opposite patterns. *S. hermaphroditum* RRF-3 achieved surprisingly high scores with several GTSF-1 proteins, even if the species itself does not have GTSF-1 anymore. In contrast, *S. ratti* RRF-3 scored very low across all tested combinations (Fig. 6C). Closer inspection revealed that the predicted structure of RRF-3 from *S. hermaphroditum* has a GID fold nearly identical to *C. elegans*, while *S. ratti* RRF-3 lacked a portion homologous to subdomain 1 of the GID (Fig. EV5D,E). These further underscore the critical role of GID in mediating GTSF-1 binding. The high GTSF-1 binding propensity of *S. hermaphroditum* RRF-3 also suggests that GTSF-1 was either recently lost or that a distant GTSF-1 homolog, which we failed to identify, still binds the RRF-3 GID.

RRF-1/EGO-1 like proteins generally did not show reliable interaction with GTSF-1 proteins. Finally, none of the PRG-1 proteins showed reliable interaction predictions with any of the nematode GTSF-1 proteins. In contrast, PRG-1 from *N. munidae*, from the Nematomorpha phylum, exhibited a high-confidence interaction with NmuGTSF-1 (Fig. 4B,C). In accordance with these findings, the aromatic residues of GTSF-1 predicted to bind PIWI proteins in mouse and fly orthologs are conserved in NmuGTSF-1, but mostly absent from the nematode orthologs (Fig. EV5A).

These findings suggest that nematode GTSF-1 maintains a conserved structural interface with RRF-3 GID, even over substantial evolutionary distances. Individual predicted interactions often carried intermediate confidence scores (0.6–0.8), so complex-by-complex comparisons should be interpreted with caution. The strength of this analysis, however, lies not in the confidence of any single prediction but in the consistency of the interaction pattern across species: we found reliable GTSF-1-RRF-3 predictions in at least one species from each major nematode lineage, including Enoplea, the sister group to all other nematodes. Notably, even in species encoding both PIWI and RRF-3, GTSF-1 preferentially interacted with RRF-3. In contrast, Nematomorpha, the sister group to Nematoda, did not encode RdRPs and showed strong GTSF-1-PIWI predictions. Thus, the functional divergence of GTSF-1 to bind RRF-3 and not Piwi is specific to Nematoda.

### Differences in nematode GTSF-1 responsible for the loss of PIWI binding

We show that GTSF-1-RRF-3 interaction is preferred over GTSF-1-PIWI throughout nematodes. Based on these conservation patterns, the most parsimonious explanation is that the GTSF-1–RRF-3 interaction was already present at the base of the nematode phylogeny. Interestingly, despite PIWI loss in numerous lineages, several nematode species have retained this Argonaute. This raises the question: did nematode PIWI proteins diverge, preventing GTSF-1 binding, or is nematode GTSF-1 incompatible with PIWI recognition?

To address this, we tested pairwise interactions between GTSF-1 and either RRF-3 or PIWI proteins from *B. mori*, *D. melanogaster*, *M. musculus*, and the nematodes *C. elegans*, *P. pacificus*, and *P. sambesi*. As expected, nematode GTSF-1 showed high-confidence interaction scores with RRF-3 orthologs but low scores with all PIWI proteins, including PRG-1 orthologs (Fig. 6D). In contrast, non-nematode Gtsf1 proteins showed favorable interaction scores with all PIWI proteins, including nematode PRG-1, but very low scores with nematode RRF-3 proteins (Fig. 6D). The lower ipTM scores with PRG-1 could be due to differences in C-terminal regions of GTSF-1/Gtsf-1 proteins, as this region has been shown to mediate specificity to cognate Piwi proteins, even among paralogs (Arif et al, 2022). Overall, we find these cross-phylum predictions reliable because the predicted Gtsf1-PRG-1 complexes closely resemble Gtsf1-PIWI structures from other animals (De et al, 2025; Portell-Montserrat et al, 2025), in that GTSF1/Gtsf1 binds PRG-1 at the Piwi domain with the central and C-terminal regions wrapped around it (Fig. EV5F).

These reciprocal interaction patterns reveal that nematode PRG-1 retains the structural capacity to bind non-nematode Gtsf1 proteins, while non-nematode Gtsf1 proteins cannot recognize nematode RRF-3. This strongly suggests that nematode PIWI proteins did not lose their GTSF1-binding potential. Instead, nematode GTSF-1 underwent evolutionary changes that eliminated its PIWI-binding capacity while establishing a specific interaction with RRF-3. This functional divergence of GTSF-1 identified within the phylum Nematoda shows a remarkable example of protein partnership switching during the evolution of small RNA pathways.

## Discussion

This study demonstrates that the function of GTSF-1 is conserved among clade V nematodes, where it associates with RRF-3 and supports its activity in the 26G-RNA pathway. In *C. elegans, C. briggsae*, and *P. pacificus*, GTSF-1 uses its zinc finger region to engage a deeply conserved N-terminal domain of RRF-3 that we term the GTSF-Interacting Domain (GID), providing a mechanistic link between GTSF-1 binding and RRF-3 function. Structural predictions further support a conserved GTSF-1-RRF-3 interface at the base of nematode phylogeny, highlighting a deep evolutionary origin for this interaction. Despite extensive work on small RNA pathways, the molecular principles governing ERI complex assembly and RdRP regulation in *C. elegans* remain poorly defined, and this study begins to address that gap.

### Conserved biochemical role of GTSF-1: from PIWI* to “RRF-3*”

Across animals, GTSF1/Gtsf1 acts as a small RNA effector cofactor that converts an inactive piRNA-guided Piwi complex into a catalytically competent state (PIWI*) (Arif et al, 2022; De et al, 2025; Portell-Montserrat et al, 2025). Biochemical reconstitution in mouse, fly, silkworm, and sponge demonstrates that GTSF1 is recruited to PIWI only after proper target engagement and extensive 3′ pairing, where it induces conformational changes that activate endonucleolytic cleavage and/or factor recruitment.

In *C. elegans* and *C. briggsae*, loss of GTSF-1 collapses the RRF-3-centered ERI complex, revealing an analogous role in licensing effector function. Size-based fractionation experiments of *C. elegans* extracts indicate that GTSF-1 exists in at least two states: a smaller pre-ERIC subcomplex with RRF-3/ERI-5 and a larger mature ERIC that includes Dicer (Almeida et al, 2018), suggesting that GTSF-1 promotes stepwise assembly of the ERI complex, conceptually paralleling PIWI* maturation in other animals. By analogy with PIWI, an attractive model is that RRF-3 surveys potential RNA substrates in a low-activity state and that productive recognition, potentially defined by sequence, structure, or cofactor-marked transcripts, triggers GTSF-1 recruitment to the GID, locking RRF-3 into an active “RRF-3*” conformation competent for dsRNA synthesis and ERIC engagement. In this view, GTSF-1-dependent conformational rearrangements license dsRNA synthesis and/or productive ERIC assembly. It follows that defining what distinguishes GTSF-1-recruiting RNAs from bystander transcripts becomes a key future question, as features that recruit GTSF-1 will provide insights into substrate selection criteria for RdRP.

Similar principles may extend beyond nematodes. In the ciliate *Paramecium tetraurelia*, PtGtsf1 functions within the Polycomb Repressive Complex 2 (PRC2) to mediate Piwi-dependent transposon repression (Charmant et al, 2024; Wang et al, 2024), even though a direct Piwi-Gtsf1 interaction has not yet been demonstrated. In that context, Gtsf1 binding likely promotes PRC2 assembly or stability in a Piwi-dependent manner, reinforcing a broader view of Gtsf1 proteins as structural cofactors that stabilize or activate functional protein assemblies across deeply divergent small RNA pathways.

### PRG-1 function without GTSF-1: evolutionary loss and functional adaptation

All biochemically characterized PIWI proteins exhibit weak intrinsic catalytic activity, remaining in an inactive or suboptimal conformation in the absence of GTSF1 (Arif et al, 2022). In most systems, GTSF1 binding to PIWI-piRNA complexes is required either to achieve efficient target cleavage or to recruit silencing factors, suggesting that loss of GTSF1 binding would render canonical PIWI function severely compromised. This framework suggests an evolutionary scenario whereby loss of GTSF-1 binding permits repeated PRG-1 loss in nematodes: an ancestral PRG-1 that could not be productively engaged by GTSF-1 would be functionally suboptimal and, over time, dispensable.

In lineages that retain PRG-1 (clade V and early-branching Chromadorea), PRG-1 may have adopted functions that diverge from canonical PIWI activity. Studies from *C. elegans* support this hypothesis. Mutations in the DDH catalytic triad of PRG-1 do not affect piRNA levels or silencing of a piRNA sensor (Bagijn et al, 2012; Pastore et al, 2024), indicating that endonucleolytic cleavage is dispensable for piRNA-mediated silencing, and PRG-1 activity is coupled to RdRP (RRF-1) activity, rather than to direct target silencing. Moreover, the phenotypic consequences of PRG-1 loss are delayed rather than acutely lethal: piRNA pathway disruption leads to progressive fertility decline over multiple generations rather than immediate sterility (Simon et al, 2014). At the molecular level*, prg-1* mutants display a striking phenotype where they accumulate aberrant 22G-RNAs on many protein-coding genes, histones, and rRNAs (Barucci et al, 2020; Montgomery et al, 2021). This idea is further strengthened by the fact that 22 G RNA biogenesis in the absence of parentally inherited 22 G RNAs can trigger acute sterility in the absence of PRG-1 (de Albuquerque et al, 2015; Phillips et al, 2015). Based on this, a currently proposed role for PRG-1 is to maintain germline homeostasis by restraining runaway small-RNA amplification. Together, these observations support a model in which clade V PRG-1 has gained GTSF-1-independent functions to act as a regulator of a small RNA network, rather than target cleavage or heterochromatin assembly.

### RdRP-GTSF1 interactions beyond nematodes

Eukaryotic RdRPs are frequently embedded in the RNAi pathway (Zong et al, 2009), yet the molecular determinants of substrate selection and their mechanisms of action remain largely unresolved. This study reveals a core function of GTSF-1 in facilitating nematode RdRP activity, specifically for RRF-3, and raises the broader hypothesis that GTSF-1-like proteins may regulate RdRPs across eukaryotes rather than exclusively acting with PIWI proteins. Beyond nematodes, multiple metazoan lineages, including cnidarians, lophotrochozoans, and chelicerates, retain RdRP genes (Feng et al, 2023; Lewis et al, 2017; Mondal et al, 2018b, 2018a; Pinzón et al, 2019; Zong et al, 2009). These provide rich comparative systems to test whether support for RdRP activity by GTSF-1-like proteins is conserved, or whether, in some lineages, a single GTSF-1-like protein may interface with both PIWI and RdRP complexes.

GTSF-1 orthologs have not yet been identified in plant species (Tidow et al, 2009; Ipsaro and Joshua-Tor, 2022), but it is intriguing to speculate that structurally similar GTSF-1–like proteins might be involved in RdRP binding in plants. The N-terminal region of ZmRDR and AtRDR2, which is structurally homologous to parts of GID, forms a positively charged, funnel-shaped RNA-guiding channel that captures single-stranded RNA templates and directs their 3′ ends into the catalytic cleft (Huang et al, 2021; Zicola, 2022; Fukudome et al, 2021; Du et al, 2022). Cryo-EM analysis of ZmRDR2 shows that RNA/NTP binding widens the cleft angles and displaces the catalytic barrel, indicating activation through N-terminal domain motion (Du et al, 2022). Mutations in these regions abolish activity without disrupting the protein’s overall fold, suggesting these domains function chiefly as conformational switches. Plants may harbor GTSF-1-like proteins that activate RdRPs by engaging their N-terminal domains through mechanisms analogous to metazoan Gtsf1-PIWI interactions.

Intriguingly, the requirement for GTSF-1 is not uniform across *C. elegans* RdRPs. RRF-1 and EGO-1 function independently of GTSF-1, generating dispersed 22G-RNAs across full-length transcripts (Aoki et al, 2007; Maniar and Fire, 2011; Sijen et al, 2007; Smardon et al, 2000). By contrast, RRF-3 is thought to produce extended dsRNA precursors that are subsequently processed by Dicer (Duchaine et al, 2006; Pavelec et al, 2009; Welker et al, 2010), a mechanism reminiscent of RdRP-Dicer interactions observed in plants and ciliates (Du, 2024; Lee and Collins, 2007; Schiebel et al, 1993; Tang et al, 2003). This suggests that long dsRNA production may impose additional requirements for processivity, substrate stabilization, or coordination with downstream processing machinery, thereby explaining why RRF-3, but not RRF-1 or EGO-1, depends on GTSF-1. Testing this model will require reconstituting RRF-3 activity with and without GTSF-1 and directly probing effects on processivity and dsRNA product length. An alternative, not mutually exclusive, possibility is that other RdRPs also rely on GTSF1-like zinc finger cofactors, but with distinct specificities. As both RRF-1 and EGO-1 harbor GID-like domains, an intriguing idea would be that these RdRPs are also activated by as-yet-unidentified zinc finger proteins that parallel GTSF-1 in domain architecture but diverge in sequence.

More broadly, proteins with tandem zinc finger arrays similar to GTSF-1 may partner with diverse eukaryotic RdRPs to promote complex assembly, substrate engagement, or conformational activation through conserved structural principles. Systematic surveys of physical interactions between RdRP and GTSF1-like factors, coupled with functional assays in these taxa, will be critical to distinguish between a nematode-specific innovation and a more ancient eukaryotic mechanism.

Taken together, this work shows how RNAi pathway components can diverge mechanistically while preserving essential regulatory functions. The diversification of GTSF-1 binding partners between PIWI and RdRP may be adaptive, enabling specialization of small RNA pathways in individual lineages, or neutral, arising from genetic drift without immediate advantage. This reflects evolving views that the complexity of RNAi pathways results from both selection for defense functions and constructive neutral processes (Torri *et al*, 2022). In summary, this work provides important evolutionary insights into the adaptability and deep evolutionary plasticity of small RNA-mediated gene silencing systems.

### Limitations of our study

While our study provides strong support for the GTSF-1 interaction site in RRF-3 through structural predictions and genetic analyses, direct high-resolution structural validation of the GTSF-1-RRF-3 complex is still lacking. In addition, our functional assays were performed primarily on clade V nematodes and a few related species, which limits the generalizability of our evolutionary conclusions. The limited availability of sequenced genomes prevented us from testing additional *Nematomorpha* species containing both PIWI and GTSF-1. Lastly, although we propose mechanistic models for how GTSF-1 activates RRF-3 and licenses ERI complex assembly, these remain speculative in the absence of direct biochemical reconstitution experiments dissecting GTSF-1’s effects on RdRP enzymatic activity and ERI complex stability. Addressing these limitations will be important for solidifying our mechanistic understanding and evolutionary inferences.

## Methods

**Table:** Reagents and tools table

Reagent/resource	Reference or source	Identifier or catalog number
**Experimental models**		
*Caenorhabditis elegans* (Bristol)	Caenorhabditis Genetics Center	N2
*Caenorhabditis briggsae*	Caenorhabditis Genetics Center	AF16
*Pristionchus pacificus*	Sommer Lab	PS312
*E. coli* DH5α competent cells	Invitrogen	18265017
*E. coli* BL21-CodonPlus (DE3)-RIL	Agilent	230245
*E. coli* BL21 DE3 Arctic express	Agilent	230192
*C. elegans gtsf-1(xf251[d10(replacement of A2 - L169 with d10)])IV*.	This study	RFK1317
*C. elegans gtsf-1(xf264[replacement with Cbr-gtsf-1 (spliced)::gfp::3×flag])IV*.	This study	RFK1318
*C. elegans gtsf-1(xf407[GGSGGG linker and 3×FLAG after E100])IV*	This study	RFK1732
*C. elegans gtsf-1(xf384 [3×flag insertion before stop codon])IV*	This study	RFK1789
*C. elegans rrf-3(pk1426)II; xfIs63[gld-1(prm)::flag::rrf-3::unc-54(3’UTR)]IV; unc-119(ed3)III*	Almeida et al, 2018	RFK405
*C. elegans rrf-3(pk1426)*	Almeida et al, 2018	RFK18
*C. elegans rrf-3(pk1426)II; xfIs63[gld-1p::flag::rrf-3::unc-54 3’UTR]IV;Cel-gtsf-1(xf407)IV*	This study	RFK1965
*C. elegans rrf-3(pk1426)II; xfIs63[gld-1p::flag::rrf-3::unc-54 3’UTR]IV;Cel-gtsf-1(xf384)IV*	This study	RFK1966
*C. elegans gtsf-1(xf373[W39A,R42A and HindIII restriction site])IV*	This study	RFK1730
*C. elegans gtsf-1(xf374[R63A and BstAPI restriction site])IV*	This study	RFK1731
*C. elegans rrf-3 (pk1426) II; xfIs63[gld-1p::flag::rrf-3::unc-54 3’UTR] IV; gtsf-1(xf43) IV*	Almeida et al, 2018	RFK421
*C. briggsae* AF16	CGC	RFK837
*C. briggsae gtsf-1(xf345)IV*	This study	RFK1609
*C. briggsae rrf-3(xf386[3×myc insertion after ATG on exon1])II*	This study	RFK1794
*C. briggsae rrf-3(xf386)II;Cbr-gtsf-1(xf345)IV*	This study	RFK1861
*P. pacificus* PS312	Sommer Lab	RFK838
*P. pacificus gtsf-1(xf208)*	Sommer Lab; This study	RFK949
**Recombinant DNA**		
Plasmids	This study	Dataset EV1D
**Antibodies**		
Rabbit anti-CbrGTSF-1	In-house affinity-purified polyclonal antibody	N/A
Rabbit anti-PpaGTSF-1	In-house affinity-purified polyclonal antibody	N/A
Normal Rabbit IgG	Sigma-Aldrich	12-370
Rabbit anti-CelGTSF-1	Almeida et al, 2018	Q5963
Anti-flag produced in rabbit	Sigma-Aldrich	F7425-2MG
Monoclonal ANTI-FLAG® M2 antibody produced in mouse	Sigma-Aldrich	F3165
Myc-Tag (9B11) Mouse mAb	CST	2276S
Anti-alpha Tubulin antibody [DM1A] - Loading Control	Abcam	ab7291
Anti-rabbit IgG, HRP-linked Antibody	CST	7074S
Anti-mouse IgG, HRP-linked Antibody	CST	7076S
**Oligonucleotides and other sequence-based reagents**		
5’rApp/NNNNTGGAATTCTCGGGTGCCAAGG/3ddC/	This study	NEXTFlex 3’ SR Adaptor
5’GUUCAGAGUUCUACAGUCCGACGAUCNNNN	This study	NEXTFlex 5’ SR Adaptor
GCTACCATAGGCACCACGAG	This study	*Cel-dpy-10 co-crispr Protospacer sequence*
TTAGATGGCGTCGGGAGAAT	This study	*Cel-gtsf-1 (xf251) Protospacer sequence*
ATCAATCGGCTTGCTTACT	This study	*Cel-gtsf-1 (xf251) Protospacer sequence*
GCTACCATAGGCACCACGAG	This study	*Cel-gtsf-1 (xf264) Protospacer sequence*
AGGAGTTCAACACGCACTTG	This study	*Cel-gtsf-1 (xf373) Protospacer sequence*
AAGGAAGTGACGCATGTTGT	This study	*Cel-gtsf-1 (xf374) Protospacer sequence*
GATCAATCGGCTTGCTTACT	This study	*Cel-gtsf-1 (xf384) Protospacer sequence*
GAACGTGGCGGAGCACCTGG	This study	*Cel-gtsf-1 (xf407) Protospacer sequence*
TGCTGATCATTGTGACTGA	This study	*Cbr-dpy-1 co-crispr Protospacer sequence*
CTATCGCCACTGATGTTGG	This study	*Cbr-dpy-1 co-crispr Protospacer sequence*
5’rApp/NNNNTGGAATTCTCGGGTGCCAAGG/3ddC/	This study	NEXTFlex 3’ SR Adaptor
5’GUUCAGAGUUCUACAGUCCGACGAUCNNNN	This study	NEXTFlex 5’ SR Adaptor
GCTACCATAGGCACCACGAG	This study	*Cel-dpy-10 co-crispr Protospacer sequence*
TTAGATGGCGTCGGGAGAAT	This study	*Cel-gtsf-1 (xf251) Protospacer sequence*
CACTTGAACTTCAATACGGCAAGATGAGAATGACTGGAAACCGTACCGCATGCGGTGCCTATGGTAGCGGAGCTTCACATGGCTTCAGACCAACAGCCTAT	This study	*Cel-dpy-10 (cn64)* ssODN sequence (5’ to 3’)
ATTTTGTGGTATAAAATAGCCGAGTTAGGAAACAAATTTTTCTTTCAGGTTTCTCAGTAGTGACCATGTGCGTGGATCTTGCGTCCACACATCTCAAGGCGTACTT	This study	*Cel-unc-58 (e665) ssODN sequence (5’ to 3’)*
CAT TTT ACT TGT TCC ACT TTT TTA AAA ATA AAA TCA CCG CTC GTG GTG CCT ATG GTA GCC ATC TAA AAT TTC GAA AAA TCA ATA AAT TCG ATG	This study	*Cel-gtsf-1 (xf251) ssODN sequence (5’ to 3’)*
catcgaatttattgatttttcgaaattttagATGGCGTCGGGAGAATCGGAGGCCCCTGGGACTTCAAAAAATGATCGATTTTCGATAACATGCCCTTACAATTCGGATCACAAGgttggaattgatatatttttaatttaaaattaaaaattttatatttcagGTGTCAATGGAGGAGTTCAACACGCACTTGTGGAAGTGTCGCACGGAAAAATTGCATTTTTACCCGCATTCGTTGAAATTGAAGAGgttttttttttgctcaattaattttccaatttttctataataattatcattattttttttagATGCTCCTACAACATGCGTCACTTCCTTCCCGAAGAAGAGCTCCAATTCCACGAGATCTTCTGCAAACGTCAATCCGCCGATCTTCGACGCCAATTGTCACTGGAGCCGCTGGAGCTGAACGTGGCGGAGCACCTGGCGGCACAGAAGCTTCGGAAAGAGTACGAGAAGGACGAGGAGTCGCTAGATGGATCAGATGACTCGGATGAAGACGAGGAGGAGAAGAATCTGTCGGTGACGTCGGAAATCGAGAAATCGGATGTGGAAGAGGTGGAGATGATGCTCGAGACGATCAATCGGCTTGCTTACTTGGAAATGAAGAATGATAATTTAATTTTGGGAGCATCGGGAGCCTCAGGAGCATCGATGAGTAAAGGAGAAGAATTGTTCACTGGAGTTGTCCCAATCCTCGTCGAGCTCGACGGAGACGTCAACGGACACAAGTTCTCCGTCTCCGGAGAGGGAGAGGGAGACGCCACCTACGGAAAGCTCACCCTCAAGTTCATCTGCACCACCGGAAAGCTCCCAGTCCCATGGCCAACCCTCGTCACCACCTTCTGCTACGGAGTCCAATGCTTCTCCCGTTACCCAGACCACATGAAGCGTCACGACTTCTTCAAGTCCGCCATGCCAGAGGGATACGTCCAAGAGCGTACCATCTTCTTCAAGgtaagtttaaacatatatatactaactactgattatttaaattttcagGACGACGGAAACTACAAGACCCGTGCCGAGGTCAAGTTCGAGGGAGACACCCTCGTCAACCGTATCGAGCTCAAGgtaagtttaaacagttcggtactaactaaccatacatatttaaattttcagGGAATCGACTTCAAGGAGGACGGAAACATCCTCGGACACAAGCTCGAGTACAACTACAACTCCCACAACGTCTACATCATGGCCGACAAGCAAAAGAACGGAATCAAGGTCAACTTCAAGgtaagtttaaacatgattttactaactaactaatcgatttaaattttcagATCCGTCACAACATCGAGGACGGATCCGTCCAACTCGCCGACCACTACCAACAAAACACCCCAATCGGAGACGGACCAGTCCTCCTCCCAGACAACCACTACCTCTCCACCCAATCCGCCCTCTCCAAGGACCCAAACGAGAAGCGTGACCACATGGTCCTCCTCGAGTTCGTCACCGCCGCCGGAATCACCCACGGAATGGACGAGCTCTACAAGGAGAATCTGTACTTTCAATCCGGAAAGgtaagtttaaaataacttcgtatagcatacattatacgaagttattttcagGGAGCCGGATCTGATTATAAAGACGATGACGATAAGCGTGACTACAAGGACGACGACGACAAGCGTGATTACAAGGATGACGATGACAAGAGATGAttttatttttaaaaaagtggaacaagtaaaatg	This study	*Cel-gtsf-1 (xf254)* PCR product sequence (5’ to 3’)
aacCTCTTCAATTTCAACGAATGCGGGTAAAAATGAAGCTTCTCAGTCGCACATTTCGCAAGGTGCGTGTTGAACTCCTCCATTGACACctgaaata	This study	*Cel-gtsf-1 (xf373) ssODN sequence (5’ to 3’)*
tctataataattatcattattttttttagATGCTCGTACAATATGGCGCACTTCCTGCCCGAAGAAGAGCTCCAATTCCACGAGATCTTCTG	This study	*Cel-gtsf-1 (xf374) ssODN sequence (5’ to 3’)*
attttacttgttccacttttttaaaaataaaaTCACTTGTCATCGTCATCCTTGTAATCCTTGTCATCGTCATCCTTGTAATCCTTGTCATCGTCATCCTTGTAATCGAGGATCAAGTTGTCGTTTTTCATCTCTAGATAAGCAAGCCGATTGATCGTCTCGAGCATCATCTCC	This study	*Cel-gtsf-1 (xf384) ssODN sequence (5’ to 3’)*
ACTGGAGCCGCTGGAGCTGAACGTGGCGGAGGGAGGTAGTGGAGGTGGAGATTACAAGGATGACGATGACAAGGATTACAAGGATGACGATGACAAGGATTACAAGGATGACGATGACAAGTGATGGCGGCACAGAAGCTTCGGAAAGAGTACGAGAAG	This study	*Cel-gtsf-1 (xf407) ssODN sequence (5’ to 3’)*
AGTGGAATTCATCTGAAAATTCTGCAGAAAACATGGAACAAAAACTTATTTCTGAAGAGGATCTTGAGCAAAAGCTCATCTCCGAGGAGGACCTCGAGCAGAAGTTGATCAGCGAGGAAGACTTGAATCAACAAAATGGCGAGTCCAGTGAAGAAGCTATCACGGTAAT	This study	*Cbr-rrf-3 (xf386) ssODN sequence (5’ to 3’)*
**Chemicals, enzymes, and other reagents**		
Recombinant Cas9-NLS	This study	N/A
Alt-R™ S.p. Cas9 Nuclease V3	IDT	1081058
Anti-cMYC magnetic beads	Sigma-Aldrich	88842
Anti-FLAG M2 magnetic beads	Sigma-Aldrich	M8823
Dynabeads™ Protein G	Thermo Fisher Scientific	10003D
Glutathione High-Capacity Magnetic Agarose Beads	Sigma-Aldrich	G0924
cOmplete™, Mini, EDTA-free Protease Inhibitor Cocktail	Roche	11836170001
TRIzol™ LS Reagent	Invitrogen	10296028
RNA 5′ Pyrophosphohydrolase	NEB	M0356S
NuPAGE™ 4–12% Bis-Tris gels	Thermo Fisher Scientific	NP0323BOX
NuPAGE 3–8% Tris-Acetate gels	Thermo Fisher Scientific	EA03752BOX
NuPAGE™ Tris-Acetate running buffer	Thermo Fisher Scientific	LA0041
NuPAGE™ MOPS running buffer	Thermo Fisher Scientific	NP0001
NuPAGE LDS sample buffer	Thermo Fisher/Invitrogen	NuPAGE LDS sample buffer
Amersham™ Protran® Western blotting nitrocellulose membranes	Sigma-Aldrich	Cat# GE10600002
SuperSignal™ West Atto Femto Maximum Sensitivity substrate	Thermo Fisher Scientific	34094
SuperSignal™ West Atto Ultimate Sensitivity substrate	Thermo Fisher Scientific	A38554
Yeast Synthetic Drop-out Medium Supplements	Sigma-Aldrich	Y2021
Yeast Nitrogen Base Without Amino Acids	Sigma-Aldrich	Y0626
Adenine	Sigma-Aldrich	A2786
L-histidine monohydrochloride monohydrate	Sigma-Aldrich	H5659
Denatured UltraPure™ Salmon Sperm DNA	Invitrogen	15632011
**Software**		
WormBase	https://wormbase.org/	N/A
TinyRNA pipeline	https://github.com/MontgomeryLab/tinyRNA	N/A
R 4.5.2	https://cran.r-project.org/bin/windows/base/	
CRISPOR	http://crispor.tefor.net	N/A
Custom Alt-R™ CRISPR-Cas9 guide RNA designer	https://eu.idtdna.com/site/order/designtool/index/CRISPR_CUSTOM	N/A
AlphaFold2	Evans et al, 2021; Jumper et al, 2021	N/A
AlphaFold3	https://alphafoldserver.com	N/A
HT-Colabfold	Brennecke Lab https://gitlab.com/BrenneckeLab/ht-colabfold	N/A
ConSurf server	https://consurf.tau.ac.il/	N/A
UCSF ChimeraX	https://www.cgl.ucsf.edu/chimerax/	N/A
PyMOL	https://www.pymol.org/	N/A
Perplexity AI	https://www.perplexity.ai/	N/A
Claude AI	https://claude.ai/	N/A
**Other**		
Pierce BCA Protein Assay Kit	Thermo Fisher Scientific	A65453
Direct-zol RNA Miniprep Kit	Zymo Research	R2050

### Nematode growth and culture

Three wild-type nematode strains were used: *C. elegans* N2 (Bristol), *C. briggsae* AF16 (Nigon and Dougherty, 1949) (Ahmedabad, India), and *P. pacificus* PS312 (Sommer et al, 1996) (Pasadena, California). AF16 was obtained from the Caenorhabditis Genetics Center, and PS312 was provided by the Sommer Lab. All strains were maintained at 20 °C on NGM plates seeded with *E. coli* OP50 under standard *C. elegans* culture conditions (Brenner, 1974). Strains are listed in Dataset EV1A and in structured methods.

### Genome editing

Genome editing in *C. elegans* and *C. briggsae* was performed using either plasmid-based Cas9 expression or direct Cas9 protein injection. For plasmid-based editing, protospacer adjacent motifs (PAMs) were identified using CRISPOR (Haeussler et al, 2016). Single guide RNAs (sgRNAs) targeting sequences 20 bp upstream of PAM sites were designed and cloned into pRK2411 (a derivative of pDD162, Addgene #47549) using site-directed, ligase-independent mutagenesis (SLIM) (Chiu et al, 2004). Constructs were transformed into DH5α competent cells (Invitrogen), selected on LB agar supplemented with 100 μg/ml ampicillin, and purified. Injection mixes contained 50 ng/μl each of Cas9/co-conversion sgRNA plasmid and gene-specific sgRNA plasmid, along with 750 nM of both co-conversion and gene-specific single-stranded DNA donor oligonucleotides (Integrated DNA Technologies, IDT). For the generation of the *Cel-gtsf-1(xf264)* strain, a 400 ng/μl PCR-derived double-stranded DNA fragment was used as the repair template instead of a single-stranded donor. For Cas9 protein-based editing, injections were performed as described previously (Paix et al, 2017). Guide sequences were selected using the Alt-R CRISPR-Cas9 design tool (IDT). Injection mixes included recombinant Cas9 protein (prepared in-house), Alt-R tracrRNA and crRNA (IDT), and single-stranded DNA donor oligonucleotides for both co-conversion and target loci. Co-conversion markers were used in both approaches: *dpy-10(cn64)* in *C. elegans* (El Mouridi et al, 2017) and Alt-R crRNA targeting *dpy-1* in *C. briggsae*. The *P. pacificus gtsf-1* mutant was generated using Cas9 protein injections (IDT Cas9 protein Cat.#1081058) as previously described (Witte et al, 2015; Han et al, 2020). Guide RNAs targeting *gtsf-1* (IDT Alt-R™ crRNA) were validated against the El Paco genome assembly (Athanasouli et al, 2023) to minimize off-target effects. All injection mixes were delivered into both gonad arms of 10–20 young adult hermaphrodites at 20 °C. F1 progeny were screened by PCR, and successful edits were confirmed by Sanger sequencing. Edited strains were outcrossed at least twice prior to downstream analysis. Protospacer sequences and donor oligonucleotides are listed in Dataset EV1B,C and in structured “Methods”.

### Brood size calculation

Animals were first synchronized by hypochlorite treatment and maintained on OP50-seeded NGM plates at 20 °C until the early L3 larval stage. Twenty to thirty individual L3 worms per strain were then singled and incubated at either 20 °C or 25–30 °C. Adult hermaphrodites were transferred daily to fresh plates for five consecutive days until egg-laying ceased. Progeny were counted 48 h post-transfer for assays at 20 °C (to facilitate accurate counting at the L3-L4 stage) or 24 h post-transfer for assays at 25–30 °C. Total brood size was calculated as the sum of all viable progeny per hermaphrodite. Data is presented as mean brood size per strain. Statistical comparisons between mutant and wild-type strains were performed using the Wilcoxon rank-sum test with Benjamini–Hochberg correction in RStudio.

### RNA interference

RNAi experiments were conducted as previously described (Fire et al, 1998; Timmons et al, 2001). For *C. elegans dpy-13* RNAi, synchronized L1 larvae were seeded on six-well plates and visually inspected for *dumpy* phenotype on the second day of adulthood.

### CbrGTSF-1 and PpaGTSF-1 antibody production

#### Recombinant GTSF-1 orthologs protein production

pET vectors containing coding sequences for tagged, full-length GTSF-1 orthologs from *C. briggsae* and *P. pacificus* (His_6_-MBP-TEVsite-GTSF-1 and His_6_-SMT3-3Csite-GTSF-1) were transformed into *E. coli* (BL21-CodonPlus (DE3)-RIL, Agilent). Cells were grown in 2 liters of LB Broth Miller (Formedium), containing 50 µg/ml Kanamycin at 37 °C/ 140 rpm until OD_600_ of 0.6. Expression was induced by the addition of 0.5 mM IPTG (neoFroxx) and further incubation at 18 °C overnight. Cells were harvested by centrifugation (4000×*g*, 15 min, 4 °C), resuspended in ice-cold lysis buffer (50 mM Tris-Cl, 500 mM NaCl, 15 mM imidazole, 1 mM MgCl_2_, 0.5 mM DTT, 1× Roche cOmplete EDTA-free protease inhibitor, 50 U/ml Sigma Benzonase, 0.1% Triton X-100, pH 8.0) and lysed by sonication at 4 °C (Branson Sonifier 450, 9 mm tip, output 7, 20% duty cycle, 2 × 3 min). Cell lysates were cleared by centrifugation (40,000×*g*, 30 min, 4 °C) and applied to a HisTrap FF 5 ml column (Cytiva) at 4 °C, using an automated chromatography system (Biorad NGC Quest Plus). The column was washed with 20 CV (column volumes) of wash buffer (50 mM Tris-Cl, 500 mM NaCl, 15 mM imidazole, 5% glycerol). Proteins were eluted from the column by applying a linear gradient of 15–500 mM imidazole (in 50 mM Tris-Cl, 500 mM NaCl, 5% glycerol) over 10 CV, and peak elution fractions were pooled for further purification. For the purification of untagged GTSF-1 orthologs, 25–50 mg of each His_6_-MBP-TEV-GTSF-1 ortholog was digested using 1 mg recombinant His_6_-TEV protease at 4 °C overnight during a 1:100 (v/v) dialysis (50 mM Tris-Cl, 200 mM NaCl, 0.5 mM DTT, 5% glycerol), using SnakeSkin dialysis tubing with 3.5 kDa cut-off (Thermo Fisher Scientific). The dialyzed solution, containing untagged GTSF-1, His_6_-MBP-TEV, and His_6_-TEV protease, was supplemented with 15 mM imidazole (pH 8.0) and applied to a HisTrap FF 5 ml column. The flow through containing untagged GTSF-1 was collected for further purification. Untagged and His_6_-SMT3-3C-tagged GTSF-1 orthologs were concentrated using Amicon Ultra-15 spin concentrators with 3 kDa cut-off (Merck Millipore) and subjected to size exclusion chromatography at 4 °C (Superdex 75 16/60 pg, Cytiva, in sterile PBS). Peak elution fractions of the proteins were analyzed by SDS-PAGE, pooled, spin-concentrated to a range of 1.5 to 4 g/l, aliquoted, flash-frozen in liquid nitrogen, and stored at −80 °C. Untagged and SMT-3-tagged *P. pacificus* GTSF-1 was oligomeric and eluted in the void volume of the Superdex 75 column.

#### Polyclonal anti-GTSF-1 orthologs antibody production

Polyclonal antibodies against recombinant, untagged *C. briggsae* and *P. pacificus* GTSF-1 were raised in rabbits, using Eurogentec’s Speedy 28-day program. In total, 200 µg of GTSF-1 in PBS were used for each immunization round. For antibody purification from serum, 2 to 3 mg of each His_6_-SMT3-3C site-tagged GTSF-1 ortholog was diluted with 4 ml coupling buffer (50 mM Tris, 5 mM EDTA, pH 8.5) and incubated with 2 ml SulfoLink bead slurry (Thermo Fisher Scientific), pre-equilibrated in coupling buffer. The proteins were covalently coupled to beads by incubation at room temperature for 2 h in a Poly-Prep column (Bio-Rad) while rotating. After washing with coupling buffer, the remaining iodoacetyl groups were blocked with 5 ml quenching buffer (50 mM L-cysteine in coupling buffer) for 30 min. The resin was subsequently washed with 10 ml coupling buffer containing 1 M NaCl, followed by 20 ml PBS. For antibody purification, 5 ml rabbit serum was diluted 1:2 with PBS, filtered through a 0.45-μm filter, and incubated with the respective GTSF-1-coupled SulfoLink resin in a Poly-Prep column overnight at 4 °C while rotating. Resins were washed with 10 ml PBS containing 0.1% Triton X-100 followed by 20 ml PBS. Antibodies were eluted with 6 ×1 ml low pH buffer (200 mM glycine-HCl, 150 mM NaCl, pH 2.3) and immediately neutralized with 0.2 ml of 1 M Tris-Cl pH 8.5. Eluted fractions were analyzed by SDS-PAGE and rebuffered into storage buffer (PBS, 10% glycerol, 0.05% NaN_3_) using PD-10 columns (Cytiva). Antibodies were spin-concentrated to 1 g/l, aliquoted, flash-frozen in liquid nitrogen, and stored at −80 °C.

### Immunoprecipitations coupled with mass spectrometry (IP-MS)

#### Sample collection and lysis

##### Sample collection and lysis for whole animals

Animals were expanded on OP50 plates for two generations, following which L1 larvae were synchronized by hypochlorite treatment and seeded onto high-density OP50 egg plates. In all, 76 h post-seeding, gravid adults were harvested by snap-freezing in liquid nitrogen (LN₂) and stored at −80 °C. For IPs, 200 µl of gravid adult samples were lysed in an equal volume of 2× lysis buffer (50 mM Tris-HCl pH 7.5, 300 mM NaCl, 3 mM MgCl₂, 2 mM DTT, 0.2% Triton X-100, and cOmplete Mini EDTA-free Protease Inhibitor Cocktail). Lysates were sonicated using a Bioruptor Plus at 4 °C for 10 cycles (30 s ON/30 s OFF), then centrifuged at 21,000×*g* for 10 min at 4 °C. Protein concentrations in the soluble lysate were measured using the Pierce BCA Protein Assay and normalized to 1–3 mg total protein with 1× lysis buffer.

##### Sample collection and lysis for embryos

Nematode embryos were collected by synchronizing L1 larvae, which were seeded onto 15 cm OP50 plates and subsequently harvested at 96 h by hypochlorite treatment. Embryos were washed extensively with cold M9, resuspended in 1× lysis buffer (25 mM Tris-HCl pH 7.0, 150 mM NaCl, 1 mM DTT, 1.5 mM MgCl₂, 0.1% Triton X-100, 1× cOmplete Mini Protease Inhibitor), and snap-frozen as droplets in liquid nitrogen for storage at −80 °C. Frozen droplets were ground to a fine powder in a liquid nitrogen-cooled mortar, then further homogenized with 40 strokes using a chilled Dounce homogenizer (pestle B). Lysates were clarified by centrifugation at 21,000×*g* for 10 min at 4 °C, and protein concentrations in the supernatant were normalized by BCA assay.

#### Immunoprecipitations

##### GTSF-1 IP-MS with anti-CbrGTSF-1, anti-PpaGTSF-1 and anti-IgG antibodies

Lysates were pre-cleared by incubation with 30 µl Dynabeads™ Protein G (equilibrated in lysis buffer) for 1–2 h at 4 °C. For each immunoprecipitation, 30 µl Dynabeads™ Protein G were washed three times with 1× wash buffer (25 mM Tris-HCl pH 7.5, 150 mM NaCl, 1.5 mM MgCl₂, 1 mM DTT, and cOmplete Mini EDTA-free Protease Inhibitor Cocktail), then conjugated to 1.5–2 µg anti-CbrGTSF-1, anti-PpaGTSF-1 (in-house), or Normal Rabbit IgG antibody (Sigma) according to the manufacturer’s instructions. Pre-cleared lysates were incubated with antibody-conjugated Dynabeads™ for 2 h at 4 °C with rotation. Beads were washed three times with wash buffer, and bound proteins were eluted in NuPAGE LDS sample buffer supplemented with 100 mM DTT by heating at 70 °C for 10 min. Eluted samples were analyzed by mass spectrometry.

##### RRF-3 IP-MS with anti-FLAG and anti-MYC antibodies

Immunoprecipitations: 30 µg *C. briggsae* embryo lysate was incubated with 10 µL anti-cMYC magnetic beads (Sigma) to capture 3×MYC::RRF-3, or 100 µg *C. elegans* embryo lysate was incubated with 15 µL anti-FLAG M2 magnetic beads (Sigma) to capture 3×FLAG::RRF-3; both reactions incubated overnight at 4 °C with rotation. Beads were magnetically separated and washed three times with wash buffer (25 mM Tris-HCl pH 7.0, 150 mM NaCl, 1 mM DTT, 1.5 mM MgCl₂, 1× cOmplete Mini Protease Inhibitor). Proteins were eluted by heating beads in 25 mM Tris-HCl pH 8.0 containing 1% SDS at 70 °C for 10 min, and eluates were stored at −20 °C for downstream processing.

#### Mass spectrometry and analysis

##### Mass spectrometry for CbrGTSF-1 IP-MS and PpaGTSF-1 IP-MS (with IgG control) experiments

Samples were separated on a 4–12% NOVEX NuPAGE gradient SDS gel (Thermo Fisher Scientific) for 10 min at 180 V in 1× MES buffer (Thermo Fisher Scientific). Proteins were fixed and stained with Coomassie G250 Brilliant Blue (Carl Roth). The gel lanes were cut, minced into pieces, and transferred to an Eppendorf tube. Gel pieces were destained with a 50% ethanol/50 mM ammonium bicarbonate (ABC) solution. Proteins were reduced in 10 mM DTT (Sigma-Aldrich) for 1 h at 56  °C and then alkylated with 5 mM iodoacetamide (Sigma-Aldrich) for 45 min at room temperature. Proteins were digested with trypsin (Sigma-Aldrich) overnight at 37  °C. Peptides were extracted from the gel by two incubations with 30% ABC/acetonitrile and three subsequent incubations with pure acetonitrile. The acetonitrile was subsequently evaporated in a concentrator (Eppendorf) and loaded onto StageTips (Rappsilber et al, 2007) for desalting and storage.

For MS analysis, peptides were separated on a 20 cm self-packed column with a 75 µm inner diameter filled with ReproSil-Pur 120 C18-AQ (Dr. Maisch) mounted to an EASY HPLC 1000 (Thermo Fisher Scientific) system and sprayed online into an Q Exactive Plus mass spectrometer (Thermo Fisher Scientific). We used a 94 min gradient from 2 to 40% acetonitrile in 0.1% formic acid at a flow rate of 225 nl min^−1^. The mass spectrometer was operated with a top 10 MS/MS data-dependent acquisition scheme per MS full scan. MS raw data were searched using the Andromeda search engine (Cox et al, 2011) integrated into the MaxQuant suite (v.1.6.5.0) (Cox and Mann, 2008) using the UniProt *C. elegans* database (August 2014; 27,814 entries). In both analyses, carbamidomethylation at cysteine was set as a fixed modification, whereas methionine oxidation and protein N-acetylation were considered variable modifications. The match-between-run option was activated. Before bioinformatic analysis, reverse hits, proteins only identified by site, protein groups based on one unique peptide, and known contaminants were removed.

For further bioinformatic analysis, the label-free quantification values were log2-transformed, and the median across the replicates was calculated. This enrichment was plotted against the −log10-transformed *P* value (Welch’s *t* test) using the ggplot2 package in the R environment.

##### Mass spectrometry for all remaining IP-MS experiments

Eluted proteins were processed using the SP3 approach (Hughes et al, 2019). The proteins were digested using trypsin overnight at 37 °C. The resultant peptide solution was purified in C_18_ StageTips (Rappsilber et al, 2003).

Peptides were separated in an in-house packed 30-cm analytical column (inner diameter: 75 μm; ReproSil-Pur 120 C18-AQ 1.9-μm beads, Dr. Maisch GmbH; heated at 50 °C) by online reverse phase chromatography through a 105-min non-linear gradient of 1.6–32% acetonitrile with 0.1% formic acid at a nanoflow rate of 225 nl/min. The eluted peptides were sprayed directly by electrospray ionization into a Q Exactive Plus Orbitrap mass spectrometer (Thermo Scientific). Mass spectrometry measurement was conducted in data-dependent acquisition mode using a top10 method with one full scan (mass range: 300–1650 *m/z*; resolution: 70,000, target value: 3 × 10^6^, maximum injection time: 20 ms) followed by 10 fragmentation scans via higher energy collision dissociation (HCD; normalized collision energy: 25%, resolution: 17,500, target value: 1 × 10^5^, maximum injection time: 120 ms, isolation window: 1.8 *m/z*). Precursor ions of unassigned or +1 charge state were rejected. Additionally, precursor ions already isolated for fragmentation were dynamically excluded for 20 s.

Mass spectrometry raw data were processed by MaxQuant software package (version 2.1.3.0) (Cox and Mann, 2008) using its built-in Andromeda search engine (Cox et al, 2011). Spectral data were searched against a target-decoy database consisting of the forward and reverse sequences of the UniProt *C. briggsae* (release 2023_02; 21,756 entries) or *P. pacificus* (release 2023_03; 26,122 entries) reference proteome, the UniProt *E. coli* (release 2023_01; 5064 entries) reference proteome, and a list of common contaminants. Trypsin/P specificity was assigned. Carbamidomethylation of cysteine was set as a fixed modification. Methionine oxidation and protein N-terminal acetylation were chosen as variable modifications. A maximum of 2 missed cleavages were tolerated. The “second peptides” options were switched on. The “match between runs” function was activated. The minimum peptide length was set to be seven amino acids. False discovery rate (FDR) was set to 1% at both peptide and protein levels.

The MaxLFQ algorithm (Cox et al, 2014) was employed for label-free protein quantification without using its default normalization option. The minimum LFQ ratio count was set to be one. Both the unique and razor peptides were used for quantification. Detected *E. coli* proteins, reverse hits, potential contaminants, and “only identified by site” protein groups were filtered out. Data normalization was performed on the log-transformed data using a median-centering approach. Proteins were further filtered to retain only those detected in at least two out of the four replicates in each group of each comparison. Following imputation of the missing LFQ intensity values, the statistical significance of the difference between the two groups was assessed using a modified t-statistic (t(SAM, statistical analysis of microarrays)) (Tusher et al, 2001) and visualized in a volcano plot. The combined significance threshold (hyperbolic curve) was defined as t0 = 1.0–1.4 and s0 = 1.5.

Results of all IP-MS experiments can be found in Datasets EV3, EV4 and within Source Data.

### GST pulldown assay

#### GST-on bead purification

Sequences of *C. elegans gtsf-1* (wild-type or mutant) and *B. mori gtsf-1* were amplified from cDNA and cloned into pGEX-6P-1 (Addgene) using Gibson assembly (Gibson et al, 2009, 2010). Plasmids were transformed into *E. coli* BL21(DE3) Arctic express cells (Agilent) and grown in 75 mL LB Broth containing 100 μg/mL ampicillin at 37 °C/140 rpm to OD₆₀₀ = 0.6. Expression was induced with 0.5 mM IPTG (Promega) and cultures incubated overnight at 18 °C. Cells were harvested by centrifugation (4000×*g*, 20 min, 4 °C) and resuspended in 5 mL ice-cold lysis buffer (50 mM Tris-HCl pH 7.5, 150 mM NaCl, 1 mM DTT, 0.1% Triton X-100, 1 μg/μL lysozyme, 1×cOmplete EDTA-free protease inhibitor). Lysates were sonicated at 4 °C (Branson Sonifier 450, 5 mm tip, output 5, 15% duty cycle, 2×4 min), then cleared by centrifugation (4000×*g*, 30 min, 4 °C). Cleared lysates were incubated with 10 μL Glutathione High-Capacity Magnetic Agarose Beads (Sigma) overnight at 18 °C with end-to-end mixing. GST-GTSF-1 beads were washed three times with 500 μL wash buffer (50 mM Tris-HCl pH 7.5, 150 mM NaCl, 1 mM DTT, 1 mM MgCl₂, 1×cOmplete EDTA-free protease inhibitor). For loading control, 10% of beads were eluted by heating at 70 °C in 1×NuPAGE™ LDS sample buffer (Invitrogen) containing 100 mM DTT. Samples were centrifuged (21,000×*g*, 5 min) and supernatants loaded onto NuPAGE™ Bis-Tris 4–12% gradient gels and run in MOPS buffer at 200 V for 50 min. GST-GTSF-1 proteins were visualized by InstantBlue™ staining.

#### GST pulldown

Remaining GST-GTSF-1 beads were incubated with *C. elegans* RFK421(*rrf-3* (*pk1426*) II; *xfIs63*[*gld-1*(*prm*)::*flag*::*rrf-3*::*unc-54* (*3’UTR*)] IV; *gtsf-1*(*xf43*) IV) embryo lysate in 1x lysis buffer (25 mM Tris-HCl pH 7.0, 150 mM NaCl, 1 mM DTT, 1.5 mM MgCl₂, 0.1% Triton X-100, 1× cOmplete Mini Protease Inhibitor) for 2 h at 4 °C with end-to-end mixing. As a positive control, 5 μL Anti-FLAG® M2 Magnetic beads (Sigma) were washed three times with embryo lysis buffer and incubated with 15% of the embryo lysate alongside GST-GTSF-1 beads. All beads were washed three times with 500 μL wash buffer (25 mM Tris-HCl pH 7.5, 150 mM NaCl, 1 mM DTT, 1.5 mM MgCl₂, 1×cOmplete Mini Protease Inhibitor). Proteins were eluted by heating at 70 °C in 1×NuPAGE™ LDS sample buffer containing 100 mM DTT, centrifuged (21,000×*g*, 5 min), and supernatants loaded onto NuPAGE™ 3–8% Tris-Acetate gels and run in Tris-Acetate buffer at 150 V for 1 h. 3×FLAG::RRF-3 was visualized by Western blot.

### Western blot

Western blotting was performed on lysates from either whole animal lysates, sonicated samples, or liquid nitrogen homogenized samples.

For whole animal, 100–200 gravid adult hermaphrodites were hand-picked and collected directly into 1×NuPAGE™ LDS sample buffer (Invitrogen) containing 100 mM DTT and heated at 95 °C for 30 min before clarification by centrifugation (21,000×*g*, 10 min) and storage at −20 °C. Sonicated or liquid nitrogen homogenized samples were prepared as described in relevant sections.

For GTSF-1 and GTSF-1::3×FLAG detection, samples were separated on NuPAGE™ 10% or 12% Bis-Tris gels in MES running buffer at 200 V for 35 min. For detection of 3×FLAG::RRF-3, samples were separated on NuPAGE™ 3–8% Tris-Acetate gels in Tris-Acetate running buffer at 150 V for 1 h. The Color Prestained Protein Standard (10–250 kDa, NEB P7719S) was used as a protein ladder.

Proteins were transferred to a 0.45-µm nitrocellulose membrane (Amersham Protan GE10600002) in NuPAGE™ Transfer Buffer with 10% methanol at 20 V overnight at 4 °C, and membranes were blocked in 5% skim milk in PBS-T (0.1% Tween-20) for 30 min. Blocked membranes were sectioned by molecular weight and incubated for 1 h at room temperature with primary antibodies in blocking buffer, followed by three 10-min washes in PBS-T. Antibody dilutions: 1:500 rabbit anti-CelGTSF-1 (Q5963 (Almeida et al, 2018)); 1:1000 rabbit anti-FLAG (Sigma-Aldrich F7425-2MG); 1:1000 mouse anti-FLAG (Sigma-Aldrich F3165); 1:1000 mouse anti-MYC (CST 2276S); 1:1000 mouse anti-α-tubulin (Abcam ab7291). For *C. elegans* GTSF-1 tagged with 3×FLAG, the rabbit anti-FLAG antibody was used, whereas all other FLAG-tagged proteins in this study were detected using the mouse anti-FLAG antibody.

Membranes were then incubated for 1 h with HRP-conjugated anti-rabbit or anti-mouse secondary antibodies (CST 7074S and 7076S), washed three times, and developed using SuperSignal™ West Atto Femto Maximum Sensitivity substrate or SuperSignal™ West Atto Ultimate Sensitivity substrate (specifically for GTSF-1) (Thermo Scientific) according to the manufacturer’s instructions.

### Yeast two-hybrid

Genes of interest were amplified from cDNA, and point mutations were introduced using the SLIM protocol (Chiu et al, 2004). Constructs were cloned into pGAD and pGBD plasmids as described previously (James et al, 1996). Double plasmid transformation was performed in *Saccharomyces cerevisiae* strain AH109 (James et al, 1996) using the high-efficiency LiAc method (Gietz and Schiestl, 2007). Briefly, cells were grown to mid-log phase (OD₆₀₀ 0.55–0.7), washed, and resuspended in 1× TE/LiOAc before being mixed with 0.15 µg each of AD and BD plasmids and 0.1 µg denatured UltraPure™ Salmon Sperm DNA (Invitrogen, 15632011). Following incubation with PEG/TE/LiOAc solution (30 °C, 30 min), cells were heat-shocked at 42 °C for 15 min with DMSO, plated on selective media supplemented with adenine and histidine, and incubated for 3 days at 30 °C. Transformed colonies were resuspended in sterile water, and OD₆₀₀ was measured using an Infinite® M200 Pro plate reader. Cells were diluted to 5 × 10⁶, 5 × 10⁵ cells/mL (assuming OD₆₀₀ 0.7 = 1 × 10⁷ cells/mL), and 5 µL of each dilution was spotted onto control plates or selection plates lacking adenine only or both adenine and histidine. Plates were imaged after 3 days of incubation at 30 °C.

All selective media were prepared using Yeast Synthetic Drop-out Medium Supplements without histidine, leucine, tryptophan, and adenine (Sigma-Aldrich, Y2021) and Yeast Nitrogen Base Without Amino Acids (Sigma-Aldrich, Y0626) at final concentrations of 1.399 g/L and 6.7 g/L, respectively. Adenine (Sigma-Aldrich, A2786) and L-histidine monohydrochloride monohydrate (Sigma-Aldrich, H5659) were added to supplemented plates at 21 mg/L and 85.6 mg/L, respectively. Bacto Agar (Sigma-Aldrich, A5306) was included at 20 g/L, and all media were supplemented with 2% glucose. Sequences tested in Yeast Two-Hybrid assays are specified in Dataset EV1E.

### RNA extraction, sequencing, and analysis

#### RNA extraction

Synchronized animals were cultured at 20 °C and harvested at gravid-adult or late-L4 stages. Embryo samples were collected from hypochlorite-treated synchronized gravid adults. All samples were collected in quadruplicates, snap-frozen in 50 µL pellets on dry ice, and stored at −80 °C until RNA extraction. For RNA extraction, pellets were resuspended in 500 µL TRIzol™ LS Reagent (Invitrogen, 10296028) and subjected to six freeze-thaw cycles (liquid nitrogen for 30 s, 37 °C water bath for 1 min). Samples were vortexed between cycles to ensure complete lysis and verified microscopically by the absence of intact worms. Debris was clarified by centrifugation at 21,000×*g* for 5 min. The supernatant was mixed with 100% ethanol (1:1 v/v) and processed with the Direct-zol™ RNA Miniprep kit (Zymo Research) according to the manufacturer’s instructions.

#### Library preparation and sequencing

##### RppH treatment

In total, 200–1000 ng RNA was subjected to RNA 5’ Pyrophosphohydrolase (RppH) treatment (NEB, M0356S) at 37 °C for 1 h to eliminate pyrophosphate from the 5´ end of triphosphorylated RNA, leaving a 5´ monophosphate RNA. Following purification, the samples were quantified using the Qubit RNA High Sensitivity Assay Kit.

##### NGS library preparation and sequencing

Next-generation sequencing (NGS) library preparation employed the NEXTflex Small RNA-Seq Kit V3, following Bioo Scientific’s standard protocol (V19.01) from Step A to Step G. The process utilized the NEXTFlex 3’ SR Adaptor and 5’ SR Adaptor (5’rApp/NNNNTGGAATTCTCGGGTGCCAAGG/3ddC/ and 5’GUUCAGAGUUCUACAGUCCGACGAUCNNNN, respectively). Step A (NEXTflex 3‘4 N Adenylated Adapter Ligation) was conducted overnight at 20 °C.

Libraries were prepared with an initial amount of 500–900 ng and amplified through 14 to 15 PCR cycles. Amplified libraries underwent purification via an 8% TBE gel, and fragments in the 15–40 nt range were size-selected. Profiling occurred using a High Sensitivity DNA Chip on a 2100 Bioanalyzer (Agilent Technologies), and quantification was performed with the Qubit dsDNA HS Assay Kit on a Qubit 2.0 Fluorometer (Life Technologies). Samples were equimolarly pooled and sequenced on a High-output NextSeq 500/550 Flowcell, single-read for 1×84 cycles, plus 7 cycles for the index read.

#### Bioinformatic analysis

Small-RNA sequencing data were analyzed using the TinyRNA pipeline (Tate et al, 2023). Raw FASTQ files were preprocessed with fastp (parameters: -a TGGAATTCTCGGGTGCCAAGG, -q 20, -e 25, –u 10) (Chen et al, 2018), during which random unique molecular identifiers (UMIs) at both the 5′ and 3′ ends were trimmed, and reads shorter than 15 nucleotides were discarded. Unique sequences were then counted and collapsed using tiny-collapse. The resulting collapsed reads were aligned to the *C. elegans* (WS279), *C. briggsae* (WS285), and *P. pacificus* (El Paco v.1) reference genomes using Bowtie (v.1.3.1) (Langmead et al, 2009).

##### Feature assignment

Aligned reads were assigned to classes (rRNA, miRNA, piRNA, 26G-RNA, and 22G-RNA) using tiny-count with custom GFF annotation files (Tate et al, 2023; Anders et al, 2014). For *C. briggsae* and *P. pacificus*, miRNA loci were sourced from miRBase v22 (Kozomara et al, 2019) and piRNA loci from previously published studies (Beltran et al, 2019). Feature assignment was based on four criteria: strand orientation, degree of genomic overlap, identity of the first 5′ nucleotide, and read length.

##### Assignment rules for *C. elegans* and *C. briggsae*

First, reads with any overlap to annotated rRNA loci were assigned as rRNAs and excluded from all downstream analyses. Of the remaining reads, those aligning in sense orientation with 5′ anchoring and a 3′-end shift value of 4 (tiny-count) to annotated mature miRNA loci were classified as miRNAs. Reads assigned as piRNAs were required to map to annotated piRNA loci in sense orientation with 5′ anchoring and be 18–21 nt in length. Finally, reads classified as 26G-RNAs or 22G-RNAs were required to map antisense to annotated genes with partial overlap, display a 5′-G bias, and be 26 nt or 21–23 nt long, respectively.

##### Assignment rules for *P. pacificus*

The same classification criteria were applied in *P. pacificus*, with the exception that rRNA-mapping reads were not identified and removed. For miRNA classification, precursor miRNA sequences were used as loci, hence a nested overlap rule was applied, such that all sense reads of any length falling within the precursor regions were counted as miRNAs.

##### Differential expression analysis

Differential expression was performed using tiny-deseq with DESeq2 (Love et al, 2014). Read counts were normalized using DESeq2’s built-in normalization and averaged across three biological replicates per genotype. Genes with fewer than 5 counts in wild-type samples were excluded. Relative abundance for each gene was calculated as the ratio of mutant counts to the sum of mutant and wild-type counts. A gene was classified as a 26G-RNA target if it was assigned to the 26G-RNA class, had DESeq2-adjusted *P* value (p_adj) < 0.05, and log₂ fold change < −2 in mutants relative to wild-type.

All tiny-RNA input and output files, including the feature assignment rules, length distribution results, feature count results, and normalized DESeq2 count tables, are provided in source data folders, alongside the computational codes used to analyze the datasets.

### AlphaFold predictions

All *C. elegans*, *C. briggsae*, and *P. pacificus* protein sequences were retrieved from WormBase using BLAST approaches. Monomer and multimer structure predictions were performed using both AlphaFold2 (Mirdita et al, 2022; Evans et al, 2021; Jumper et al, 2021) and AlphaFold3 (Abramson et al, 2024) (https://alphafoldserver.com; with 2 copies of Zn^2+^ where specified). To predict potential protein–protein interactions via high-throughput screening, amino acid sequences were retrieved from UniProt or reciprocal BLAST and formatted as FASTA files. Two sequence sets were generated: a bait and a prey file. All pairwise combinations between bait and prey sequences were systematically screened using the ht-colabfold pipeline (https://gitlab.com/BrenneckeLab/ht-colabfold) as previously described (Hohmann et al, 2025), which automates large-scale structure prediction with AlphaFold-Multimer (v2) (Mirdita et al, 2022; Evans et al, 2021; Jumper et al, 2021) via ColabFold (Mirdita et al, 2022). Predictions were conducted at the Life Science Compute Cluster (LiSC) of the University of Vienna, using default parameters, including multiple sequence alignments generated via MMseqs (Mirdita et al, 2022). Structural models were ranked based on ipTM and PEAK scores (Hohmann et al, 2025). The details of confidence scores resulting from all AlphaFold predictions are provided in Dataset EV2. Individual analysis files, including the bait-prey sequences used in HT-ColabFold are provided in Dataset EV1G and source data.

### ConSurf analysis

Conservation analysis of GTSF-1 was performed using orthologous sequences from 26 nematode species: *C. elegans*, *C. briggsae*, *C. nigoni*, *C. brenneri*, *C. tropicalis*, *C. doughertyi*, *C. inopinata*, *C. kamaaina*, *C. japonica*, *C. plicata*, *C. virilis*, *C. castelli*, *C. nouraguensis*, *C. sp21*, *C. sp26*, *C. sp28*, *C. sp29*, *C. sp31*, *C. sp38*, *C. sp39*, *C. sp40*, *P. pacificus*, *Toxocara canis*, *Wuchereria bancrofti*, *Brugia malayi*, and *Brugia pahangi*. Sequences were obtained from UniProt through BLAST using *C. elegans* GTSF-1 as a reference.

Orthologous RRF-3 protein sequences were retrieved from UniProt for 11 nematode species: *C. elegans*, *C. briggsae*, *C. nigoni*, *C. remanei*, *C. latens*, *C. brenneri*, *C. angaria*, *C. bovis*, *C. japonica*, *C. sp36*, and *P. pacificus*. Multiple sequence alignment was generated using Clustal Omega (https://www.ebi.ac.uk/jdispatcher/msa/clustalo) and submitted to the ConSurf server (https://consurf.tau.ac.il/) (Ashkenazy et al, 2016) along with the top-ranking AlphaFold2 prediction of GTSF-1 monomer for GTSF-1 scoring or GTSF-1–RRF-3 complex for RRF-3 scoring. ConSurf assigned conservation grades on a 1–9 scale (1 = variable, 9 = highly conserved) to each residue. The output structure file with mapped conservation scores was visualized and analyzed in ChimeraX. All sequences used for ConSurf, as well as the output files, are provided in the source data.

### Genome data

Protein coding gene sets from 69 publicly available nematode genomes were analyzed, representing all five major nematode clades (I-V). To provide an outgroup, four genomes from the sister phylum Nematomorpha, *Acutogordius australiensis*, *Nectonema munidae*, *Gordionus montsenyensis*, and *Gordius aquaticus*, were additionally included. Summary of species datasets provided in Dataset EV1F.

### Identification of RNAi pathway orthologues

To identify orthologues of the core *C. elegans* RNAi pathway proteins, we assembled a curated elainereference set consisting of PRG-1, GTSF-1, EGO-1, RRF-1, and RRF-3 from the *C. elegans* genome on Wormbase (WS276). Orthologues in each target species were identified using the reciprocal best BLASTP hit (RBH) approach, a widely established method for orthology inference. A candidate gene was retained as a putative one-to-one orthologue only if each sequence was the top BLASTP hit of the other under identical search parameters. This strict reciprocal criterion provided a conservative baseline for orthologue detection (Tatusov et al, 1997; Moreno-Hagelsieb and Latimer, 2008).

### Figures

Molecular graphics and analyses performed with UCSF ChimeraX (Goddard et al, 2018; Meng et al, 2023). Electrostatic surface calculations were performed with APBS (Jurrus et al, 2018) in PyMOL with a solvent ion concentration of 0.15 M using the AMBER force field. The ggplot2 package of RStudio was used to produce graphs (Hadley Wickham, 216AD).

### Statement on blinding

Experiments were not performed under blinded conditions.

## Supplementary information

Peer Review File

Dataset EV1

Dataset EV2

Dataset EV3

Dataset EV4

Source data Fig. 1

Source data Fig. 2

Source data Fig. 3

Source data Fig. 4

Source data Fig. 5

Source data Fig. 6

Figure EV1 Source Data

Figure EV2 Source Data

Figure EV4 Source Data

Expanded View Figures

## Data Availability

All sequencing data generated for this study have been uploaded to the European Nucleotide Archive (www.ebi.ac.uk) under accession number PRJEB105295. All mass spectrometry data generated in this study have been uploaded to the UCSD Mass Spectrometry Interactive Virtual Environment (MassIVE) and to the PRoteomics IDEntifications Database (PRIDE) server. Mass spectrometry data for GTSF-1 immunoprecipitation in *C. briggsae* and *P. pacificus* are available under PRIDE accession number PXD072041 and MassIVE accession number MSV000100223 (https://massive.ucsd.edu/ProteoSAFe/dataset.jsp?task=0d16e353aac54dee9013906ea4f76067). Data for RRF-3 immunoprecipitation in *C. briggsae* and RRF-3/GTSF-1 dual immunoprecipitation in *C. elegans* are under MassIVE accession numbers MSV000100221 (https://massive.ucsd.edu/ProteoSAFe/dataset.jsp?task=b6cf9263c74242f6868ff0f0c29860b0) and MSV000100222 (https://massive.ucsd.edu/ProteoSAFe/dataset.jsp?task=4d154433bbb544d694e59afc66e8fbc6), respectively. The source data of this paper are collected in the following database record: biostudies:S-SCDT-10_1038-S44319-026-00914-1.
