# Supplementary material for: An evolutionarily conserved N-terminal domain of RRF-3 governs GTSF-1 binding in nematodes
Source: EMBO Rep. 2026 Aug 29;27(19):5530–60. doi: 10.1038/s44319-026-00914-1 (PMC13650160; doi:10.1038/s44319-026-00914-1)
Supplement: Supplementary file 1 [file 44319_2026_914_MOESM15_ESM.pdf]

Expanded View Figures

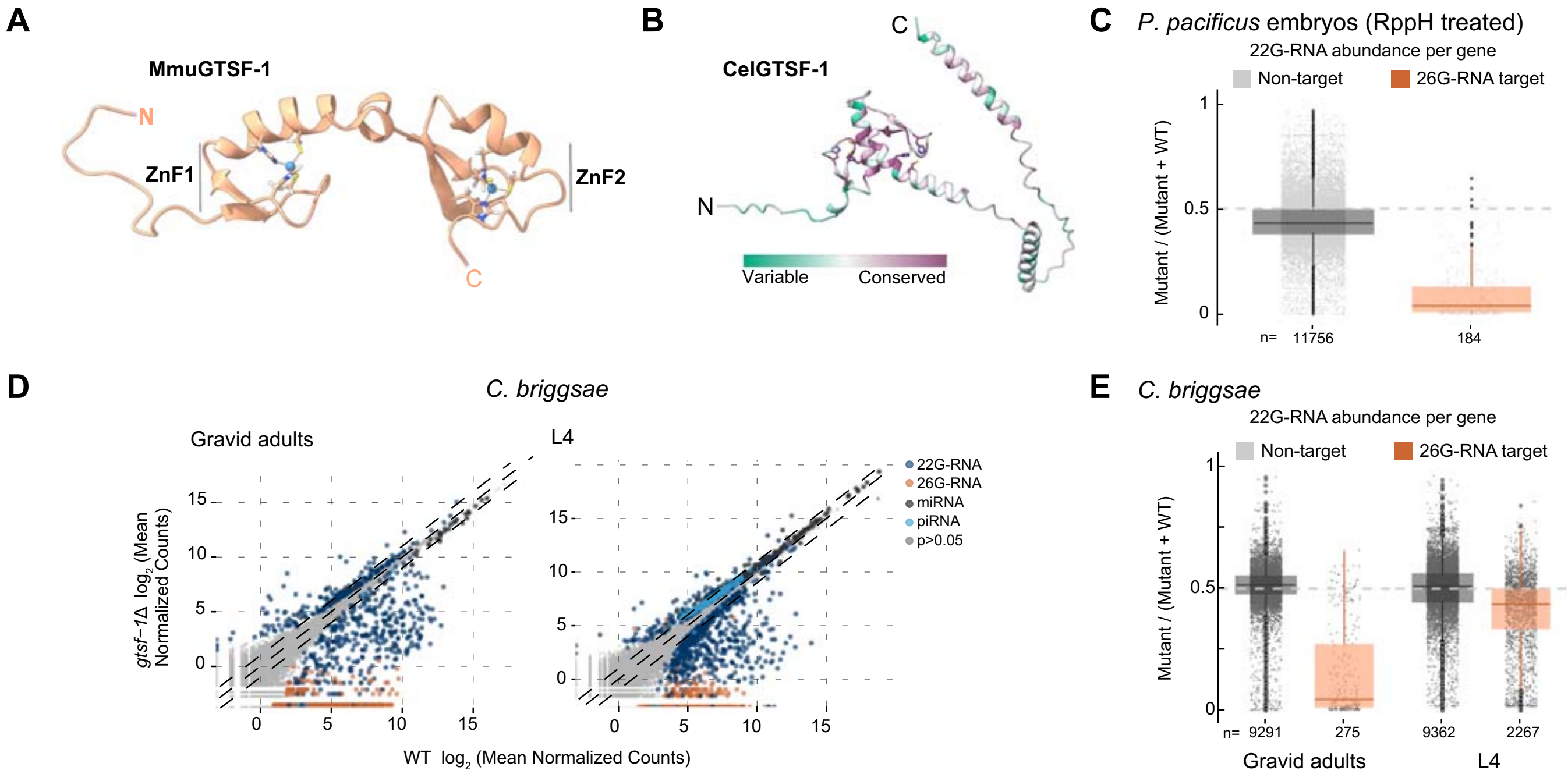

Figure EV1. GTSF-1 structure prediction and RppH-treated small RNA sequencing in *gtsf-1* mutants.

(**A**) *M. musculus* NMR structure (NMR_6X46L (Ipsaro et al, 2021)). CHHC residues comprising zinc fingers are displayed in stick format. (**B**) AlphaFold2 monomer prediction of GTSF-1 colored by ConSurf conservation score (1:Variable to 9:Conserved) calculated from an MSA of 26 GTSF-1 orthologs (*C. elegans, C. briggsae, C. inopinata, P. pacificus, C. brenneri, C. castelli, C. doughertyi, C. japonica, C. kamaaina, C. nouraguensis, C. plicata, C. sp21, C. sp26, C. sp28, C. sp29, C. sp31, C. sp38, C. sp39, C. sp40, C. tropicalis, C. virilis, C. nigoni, Toxocara canis, Brugia malayi, Wuchereria bancrofti, Brugia pahangi*). 'N' and 'C' denote N and C-terminal residue. (**C**) *P. pacificus* embryos RppH-treated libraries: Abundance of DESeq2-normalized 22G-RNA counts per gene between wild-type and *gtsf-1* deletion samples as box plots. Abundance calculated as counts in deletion samples divided by total counts. $n =$ number of genes per category. Box plots show the median, 25th–75th percentiles (box), and whiskers extending to the most extreme value within 1.5× the interquartile range, with data shown as jitter points and outliers as individual points. (**D**, **E**) RppH-treated libraries comparing wild-type and *gtsf-1* deletion animals in *C. briggsae* gravid adults and L4 larvae. (**D**) Normalized counts (DESeq2) averaged across three biological replicates depicted as a scatter plot. *P* value calculated using the Wald test. Colored points: $P < 0.05$. Solid line: equal expression; dotted lines: ±2-fold change. (**E**) DESeq2-Normalized 22G-RNA count abundance per gene for 26G-RNA target or non-target genes. Data includes three biological replicates of each sample. Box plots show the median, 25th–75th percentiles (box), and whiskers extending to the most extreme value within 1.5× the interquartile range, with data shown as jitter points and outliers as individual points. Source data are available online for this figure.

 © The Author(s)

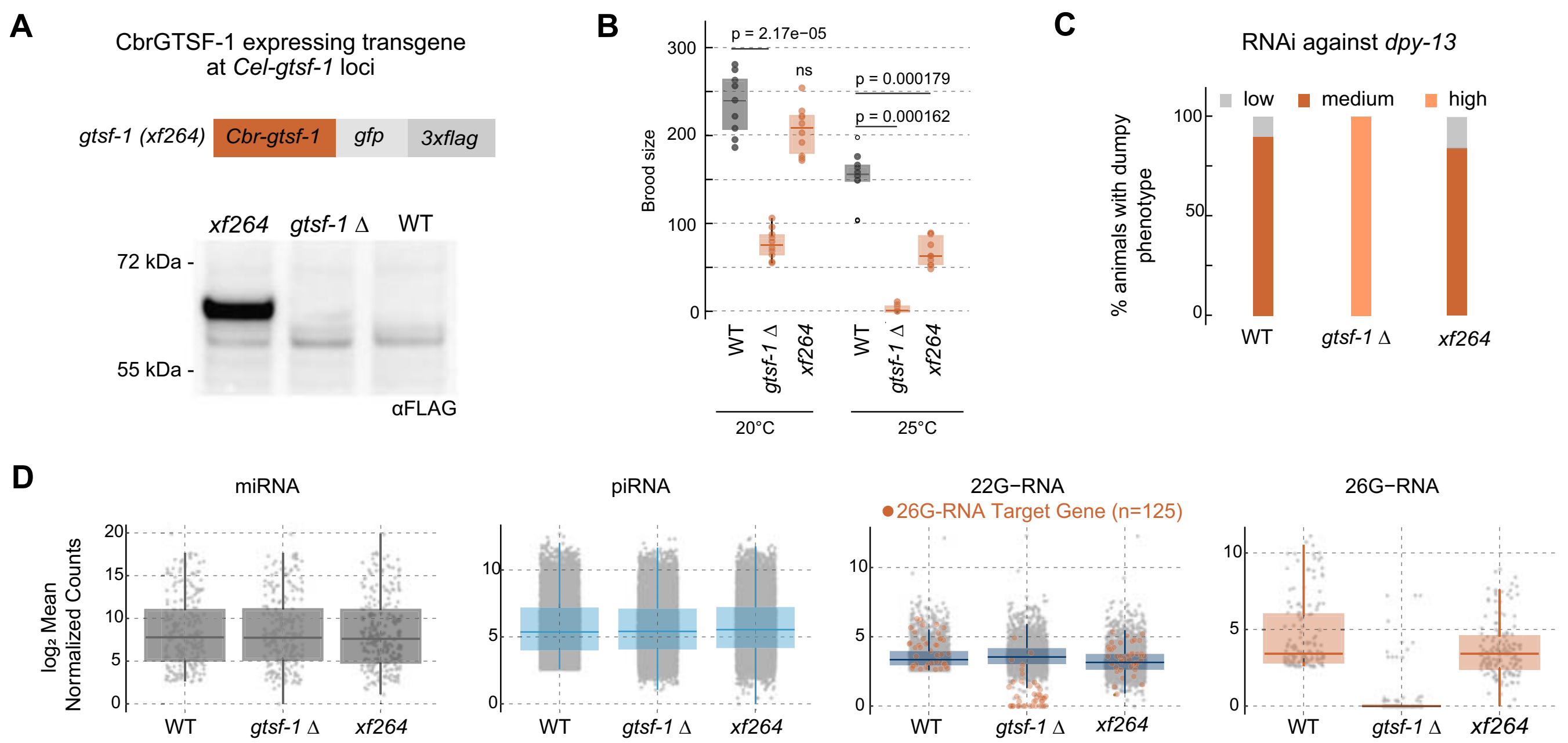

Figure EV2. Transgenic *C. briggsae gtsf-1* partially rescues *C. elegans gtsf-1* deletion.

(**A**) Top: Schematic of *Cel-gtsf-1(xf264)* allele with endogenous *gtsf-1* replaced by *C. briggsae gtsf-1* transgene upstream of *gfp* and *3×flag*. Bottom: Western blot from gravid adults validating transgene expression. (**B**) Brood size quantification ($n = 15$–25) as box plots. Box plots show the median, 25th–75th percentiles (box), and whiskers extending to the most extreme value within 1.5× the interquartile range, with data shown as jitter points and outliers as individual points. *P* values: Wilcoxon rank-sum test with BH correction. ns: Non-significant compared to wild-type. (**C**) RNAi responses to *dpy-13* scored by *dumpy* phenotype penetrance and severity (low, medium, high) based on the visual ratio of body length to thickness. High severity: L2-like length with extreme thickening causing immobility. (**D**) Small-RNA profiles for *C. elegans gtsf-1* mutant animals (Gravid adults; Untreated libraries). Plot displays normalized counts per gene for small-RNA types. DESeq2-normalized counts averaged across three replicates. Colored 22G-RNA counts: 26G-RNA target genes identified in this dataset. Data includes three biological replicates of each sample. Source data are available online for this figure.

© The Author(s)

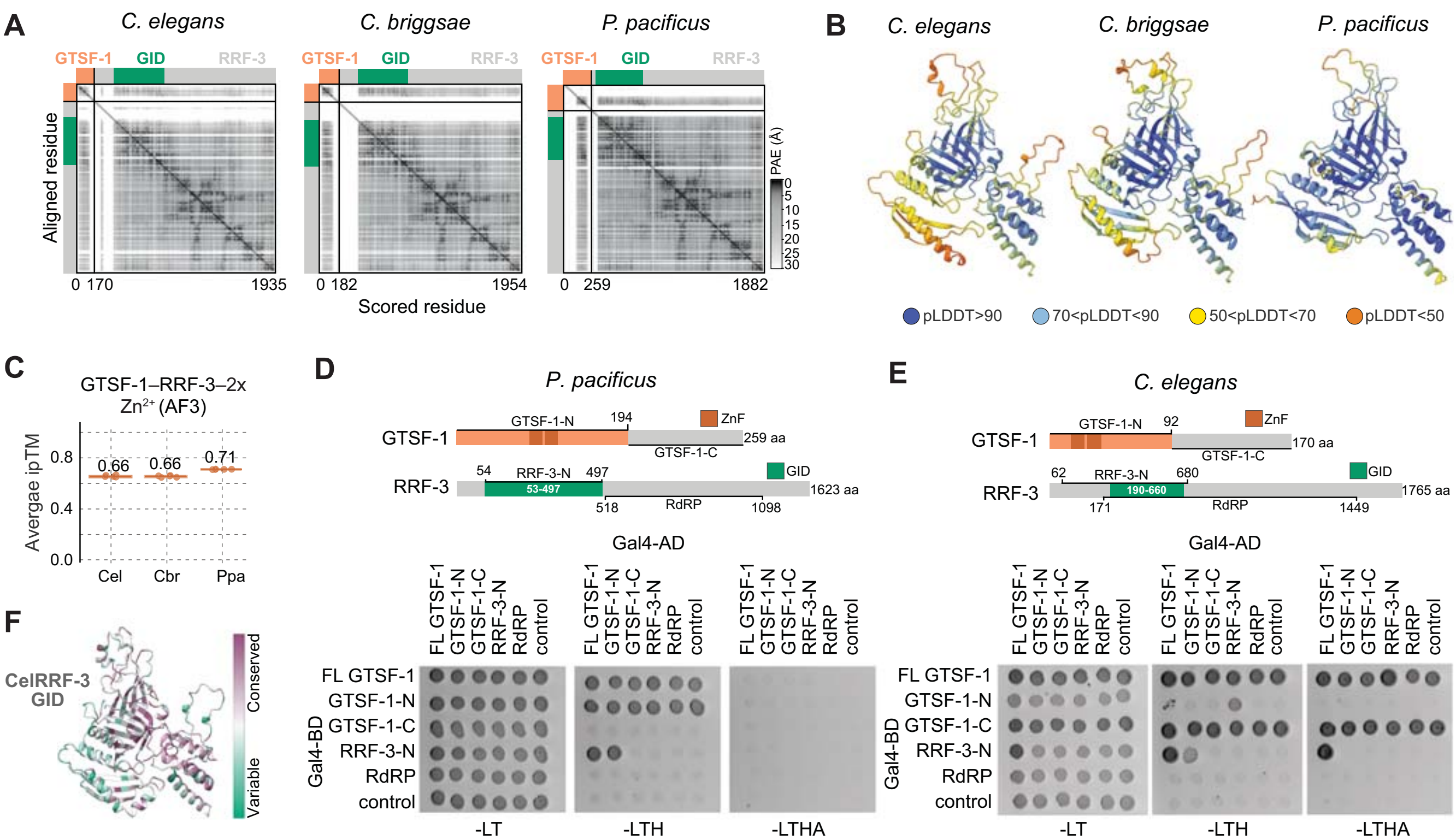

Figure EV3. The GTSF-1–RRF-3-GID interaction is conserved in *C. elegans*, *C. briggsae*, and *P. pacificus*.

(**A**) PAE plots for GTSF-1–RRF-3 AF2 complexes displayed in Fig. 4A. (**B**) RRF-3-GID regions colored by pLDDT (0-100). (**C**) Average ipTM from AlphaFold3 Multimer models of GTSF-1–RRF-3-2xZn2+ complexes ($n = 5$). (**D**, **E**) Yeast Two-Hybrid assays between GTSF-1 and RRF-3 regions. Plates show selection (-LT: -Leu, -Trp), low stringency (-LTH: -Leu, -Trp, -His), and high stringency (-LTHA: -Leu, -Trp, -His, -Ade) conditions. Control: Gal4 activation domain (AD) or DNA-binding domain (BD). Experiment tested through two biological replicates. (**F**) ConSurf conservation scores mapped onto *C. elegans* RRF-3-GID. Scores were calculated from an MSA of 11 RRF-3 orthologs (*C. elegans, C. briggsae, C. remanei, C. latens, C. nigoni, C. brenneri, C. japonica, C. sp.36, C. angaria, C. bovis*, and *P. pacificus*). Scores range from 1 to 9, colored from turquoise (score 1; highly variable) to maroon (score 9; highly conserved).

 © The Author(s)

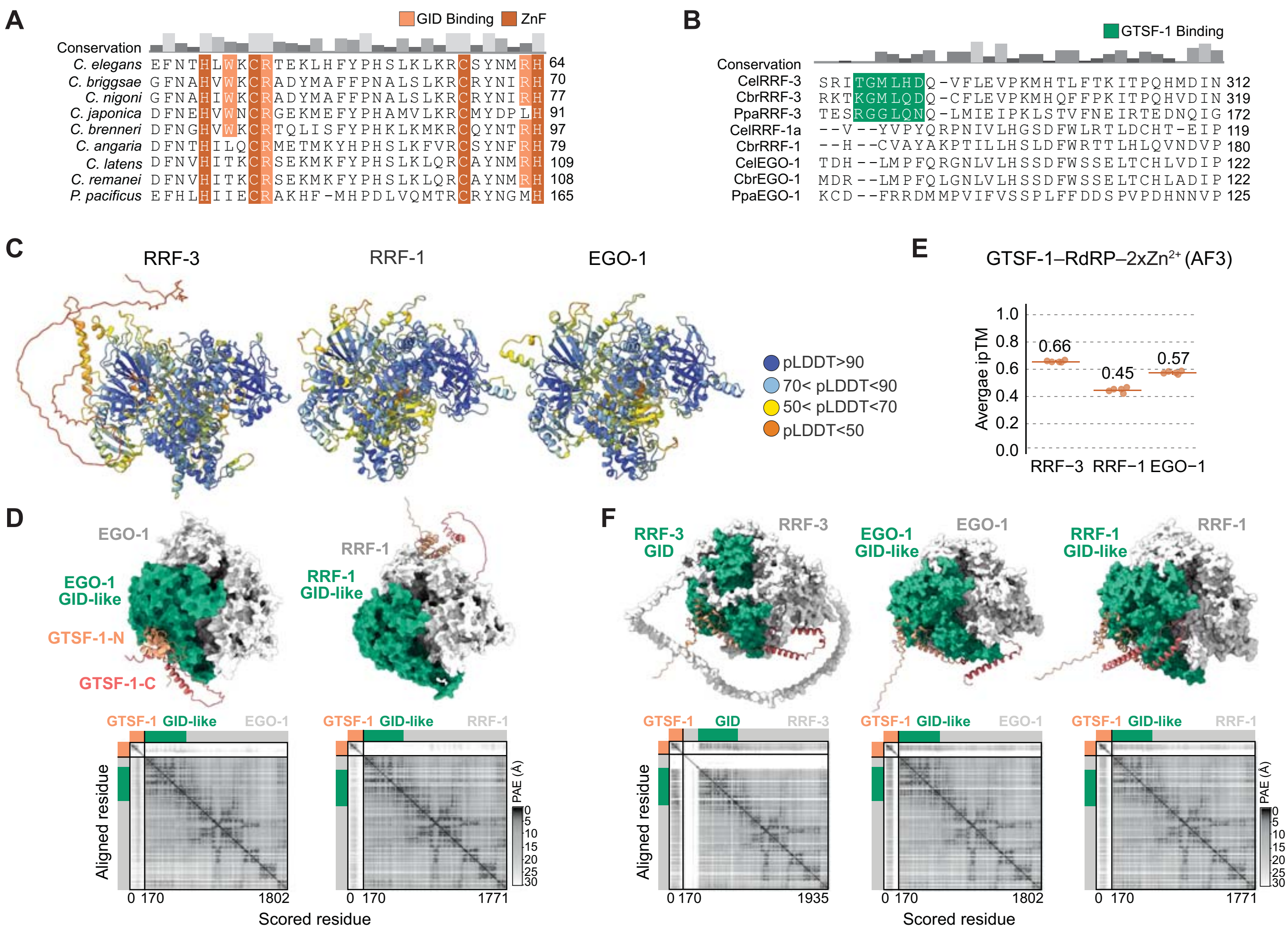

Figure EV4. AF3 predicts a conserved interaction mode of GTSF-1 with RRF-1 and EGO-1.

(**A**) Clustal Omega MSA of nematode GTSF-1 orthologs. Highlighted residues part of CHHC Zinc fingers (ZnF) and experimentally tested to bind RRF-3 GID. (**B**) Clustal Omega MSA of RdRP sequences. Species: Cel, *C. elegans*; Cbr, *C. briggsae*; Ppa, *P. pacificus*. Residues within the RRF-3 GID that form the GTSF-1 binding interface are highlighted. (**C**) AF2 monomer structures of *C. elegans* RdRPs colored by pLDDT confidence score. (**D**) Top-ranked AF2 multimer predictions of *C. elegans* GTSF-1 in complex with RRF-1 or EGO-1, displayed as surface models alongside their respective PAE plots. GID-like denotes the region in RRF-1 and EGO-1 homologous to the RRF-3 GID. GTSF-1-N and C are N-terminal and C-terminal regions of GTSF-1 respectively. (**E**, **F**) AF3 multimer predictions of *C. elegans* GTSF-1-RdRP–2×Zn2+complexes. (**E**) Average ipTM scores from $n = 5$ predictions. (**F**) Surface models of the top-ranked predictions alongside their respective PAE plots. Source data are available online for this figure.

© The Author(s)

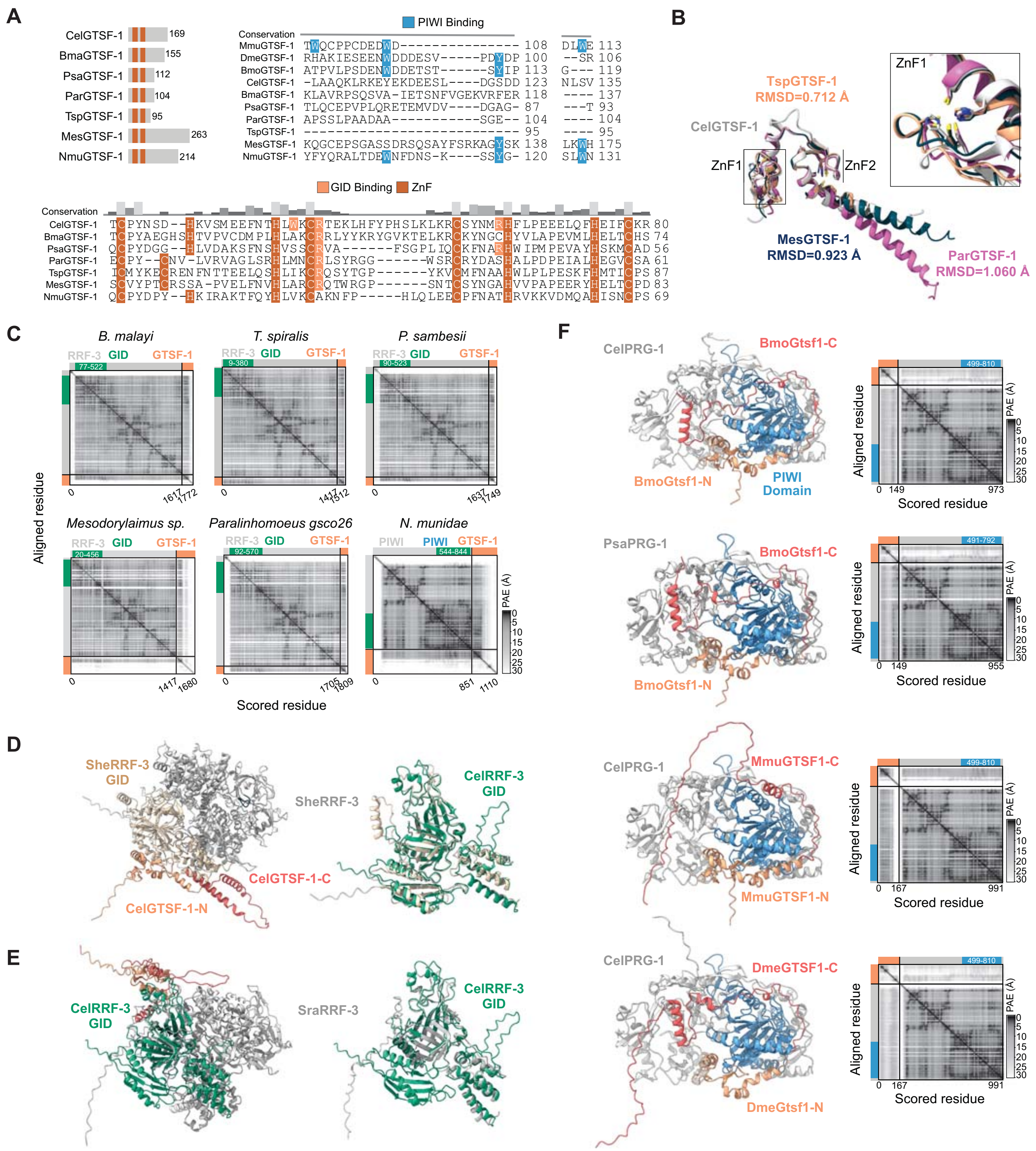
A
PIWI Binding
Conservation
CelGTSF-1
BmaGTSF-1
PsaGTSF-1
ParGTSF-1
TspGTSF-1
MesGTSF-1
NmuGTSF-1
MmuGTSF-1
DmeGTSF-1
BmoGTSF-1
GID Binding
ZnF
B
ZnF1
ZnF2
TspGTSF-1 RMSD=0.712 Å
CelGTSF-1
MesGTSF-1 RMSD=0.923 Å
ParGTSF-1 RMSD=1.060 Å
C
B. malayi
T. spiralis
P. sambesii
Mesodorylaimus sp.
Paralinhomoeus gsco26
N. munidae
RRF-3
GID
GTSF-1
PIWI
Aligned residue
Scored residue
PAE (Å)
D
SheRRF-3 GID
SheRRF-3
CelRRF-3 GID
CelGTSF-1-C
CelGTSF-1-N
E
CelRRF-3 GID
SraRRF-3
F
CelPRG-1
BmoGtsf1-C
BmoGtsf1-N
PIWI Domain
PsaPRG-1
MmuGTSF1-C
MmuGTSF1-N
DmeGTSF1-C
DmeGtsf1-N

 © The Author(s)

Figure EV5. GTSF-1 structure and interaction mode with RRF-3 are conserved across the nematode phylum.

(**A**) Left: Schematic of GTSF-1 lengths from selected nematodes. Right, Bottom: MSA of GTSF-1 regions. Orange boxes: CHHC zinc-finger residues and residues tested for GID binding. Blue boxes: Residues tested for PIWI binding in *D. melanogaster* (Dönertas et al, 2013) and *M. musculus* (Yoshimura et al, 2018), Species: Cel, *C. elegans*; Bma, *B. malayi*; Psa, *P. sambesi*; Par, *Paralinhomoeus gsco26*; Tsp, *T. spiralis*; Mes, *Mesodorylaimus sp*.; Nmu, *N. munidae*. (**B**) Superimposed structures of GTSF-1 from *C. elegans* (Cel), *T. spiralis* (Tsp), *Mesodorylaimus sp*. (Mes), and *Paralinhomoeus gsco26* (Par) panel zoom on CCHC type zinc finger 1 (ZnF1). *C. elegans* GTSF-1 (residues 17–92) was used as the reference, and Cα RMSD values were calculated. RMSD: 0.712 Å for *T. spiralis* (residues 1–95), 0.923 Å *for Mesodorylaimus sp*. (residues 23–97), and 1.060 Å *Paralinhomoeus gsco26* (residues 1–76). (**C**) PAE plots of AlphaFold2 structures from Fig. 6C. (**D**) AF2 multimer of *C. elegans* GTSF-1 with *S. hermaphroditum* (She) RRF-3, Superimposed with *C. elegans* RRF-3 GID. Right: Superimposed structures of *C. elegans* RRF-3 GID with *S. hermaphroditum* residues 1-461, Cα RMSD = 0.920. (**E**) AF2 multimer of *C. elegans* GTSF-1 with *S. ratti* (Sra) RRF-3, superimposed with *C. elegans* RRF-3 GID. Right: Superimposed structures of *C. elegans* RRF-3 GID with *S. ratti* residues 1-364, Cα RMSD = 0.845. (**F**) AF2 structures of top-ranked PRG-1-GTSF1/Gtsf1 complexes from Fig. 6D alongside respective PAE plots.

© The Author(s)
