# Supplementary material for: An evolutionarily conserved N-terminal domain of RRF-3 governs GTSF-1 binding in nematodes
Source: EMBO Rep. 2026 Aug 29;27(19):5530–60. doi: 10.1038/s44319-026-00914-1 (PMC13650160; doi:10.1038/s44319-026-00914-1)
Supplement: Supplementary file 2 [file 44319_2026_914_MOESM1_ESM.pdf]

EMBO reports

An Evolutionarily Conserved N-terminal Domain of RRF-3 Governs GTSF-1 Binding in Nematodes

Shamitha Govind, Sebastian Ruppert, Joseph Kirangwa, Virginia Busetto, Emily Nischwitz, Miguel Almeida, Svenja Hellmann, Hahn Witte, Ralf Sommer, Falk Butter, Sebastian Falk, Peter Sarkies, and Rene Ketting

Corresponding author(s): Rene Ketting (r.ketting@imb-mainz.de)

Review Timeline:

Submission Date:	20th Jan 26
Editorial Decision:	17th Feb 26
Revision Received:	24th Jun 26
Editorial Decision:	20th Jul 26
Revision Received:	10th Aug 26
Accepted:	17th Aug 26

Editor: Yehu Moran / Esther Schnapp

Transaction Report:

(Note: With the exception of the correction of typographical or spelling errors that could be a source of ambiguity, letters and reports are not edited. Depending on transfer agreements, referee reports obtained elsewhere may or may not be included in this compilation. Referee reports are anonymous unless the Referee chooses to sign their reports.)

Dear Prof. Ketting

Thank you for the submission of your manuscript to EMBO Reports. We have now received the full set of referee reports that you can find below.

As you will see, the referees acknowledge that the findings are of interest to the community. Yet, they do raise some substantial comments that require your attention.

I would thus like to invite you to revise your manuscript with the understanding that the referee concerns must be fully addressed and their suggestions taken on board whenever possible. Please address all referee concerns in a complete point-by-point response. Acceptance of the manuscript will depend on a positive outcome of a second round of review. It is EMBO Reports policy to allow a single round of major revision only and acceptance or rejection of the manuscript will therefore depend on the completeness of your responses included in the next version of the manuscript.

We realize that it is difficult to revise to a specific deadline. In the interest of protecting the conceptual advance provided by the work, we recommend a revision within 3 months (20th May 2026). Please discuss the revision progress ahead of this time with the editor if you require more time to complete the revisions.

You can either publish the study as a short report or as a full article. For short reports, the revised manuscript should not exceed 27,000 characters (including spaces but excluding materials & methods and references) and 5 main plus 5 expanded view figures. The results and discussion sections must further be combined, which will help to shorten the manuscript text by eliminating some redundancy that is inevitable when discussing the same experiments twice. For a normal article there are no length limitations, but it should have more than 5 main figures and the results and discussion sections must be separate. In both cases, the entire materials and methods must be included in the main manuscript file.

IMPORTANT NOTE: we perform an initial quality control of all revised manuscripts before re-review. Your manuscript will FAIL this control and the handling will be DELAYED if the following APPLIES:
1) A data availability section providing access to data deposited in public databases is missing. If you have not deposited any data, please add a sentence to the data availability section that explains that.
2) Your manuscript contains statistics and error bars based on n=2. Please use scatter blots in these cases. No statistics should be calculated if n=2.

When submitting your revised manuscript, please review the submission guidelines (https://link.springer.com/journal/44319/submission-guidelines#cms-Revised-submissions) and carefully review the instructions that follow below. Failure to include requested items will delay the evaluation of your revision.

1) a .docx formatted version of the manuscript text (including legends for main figures, EV figures and tables). Please make sure that the changes are highlighted to be clearly visible.

2) individual production quality figure files as .eps, .tif, .jpg (one file per figure). See hhttps://media.springernature.com/original/springer-cms/rest/v1/content/27825798/data/v1 for more info on how to prepare your figures.

3) We replaced Supplementary Information with Expanded View (EV) Figures and Tables that are collapsible/expandable online. A maximum of 5 EV Figures can be typeset. EV Figures should be cited as 'Figure EV1, Figure EV2" etc... in the text and their respective legends should be included in the main text after the legends of regular figures.

- For the figures that you do NOT wish to display as Expanded View figures, they should be bundled together with their legends in a single PDF file called *Appendix*, which should start with a short Table of Content. Appendix figures should be referred to in the main text as: "Appendix Figure S1, Appendix Figure S2" etc. See 'Expanded View' section of the submission guidelines.

- Additional Tables/Datasets should be labeled and referred to as Table EV1, Dataset EV1, etc. Legends have to be provided in a separate tab in case of .xls files. Alternatively, the legend can be supplied as a separate text file (README) and zipped together with the Table/Dataset file.

4) a .docx formatted letter INCLUDING the reviewers' reports and your detailed point-by-point responses to their comments. As part of the EMBO Press transparent editorial process, the point-by-point response is part of the Review Process File (RPF), which will be published alongside your paper.

5) a complete author checklist, which you can download from our author guidelines. Please insert information in the checklist that is also reflected in the manuscript. The completed author checklist will also be part of the RPF.

6) Please note that all corresponding authors are required to supply an ORCID ID for their name upon submission of a revised manuscript (<https://orcid.org/>), which can be linked to your profile in the manuscript submission system.

7) Before submitting your revision, primary datasets produced in this study need to be deposited in an appropriate public database. Please remember to provide a reviewer password if the datasets are not yet public. The accession numbers and database should be listed in a formal "Data Availability" section placed after Methods. Please note that the Data Availability Section is restricted to new primary data that are part of this study. * Note - All links should resolve to a page where the data can be accessed. *

If your study has not produced novel datasets, please mention this fact in the Data Availability Section.

8) At EMBO Press we ask authors to provide source data for the main manuscript figures. You will receive a separate email with instructions for providing source data with your revised manuscript, including how to upload and organize the files.

9) Our journal also encourages inclusion of *data citations in the reference list* to directly cite datasets that were re-used and obtained from public databases. Data citations in the article text are distinct from normal bibliographical citations and should directly link to the database records from which the data can be accessed. In the main text, data citations are formatted as follows: "Data ref: Smith et al, 2001" or "Data ref: NCBI Sequence Read Archive PRJNA342805, 2017". In the Reference list, data citations must be labeled with "[DATASET]". A data reference must provide the database name, accession number/identifiers and a resolvable link to the landing page from which the data can be accessed at the end of the reference.

10) Regarding data quantification, the following points must be specified in each figure legend:

- the name of the statistical test used to generate error bars and P values,

- the number (n) of independent experiments (please specify technical or biological replicates) underlying each data point,

- the nature of the bars and error bars (s.d., s.e.m.),

- If the data are obtained from n Program fragment delivered error ``Can't locate object method "less" via package "than" (perhaps you forgot to load "than"?) at //ejpvfs23/sites23b/embor_www/letters/embor_decision_revise_and_review.txt line 54.' 2, use scatter blots showing the individual data points.

Discussion of statistical methodology can be reported in the materials and methods section, but figure legends should contain a basic description of n, P and the test applied.

- Please also include scale bars in all microscopy images.

11) The journal requires a statement specifying whether or not authors have competing interests (defined as all potential or actual interests that could be perceived to influence the presentation or interpretation of an article). In case of competing interests, this must be specified in your disclosure statement.

12) All Materials and Methods need to be described in the main text using our 'Structured Methods' format, which is required for all research articles. According to this format, the Methods section includes a Reagents and Tools Table (listing key reagents, experimental models, software and relevant equipment and including their sources and relevant identifiers) followed by a Methods and Protocols section describing the methods using a step-by-step protocol format. The aim is to facilitate adoption of the methodologies across labs. More information on how to adhere to this format as well as a downloadable template (.docx) for the Reagents and Tools Table can be found in our author guidelines.

We would also welcome the submission of cover suggestions, or motifs to be used by our Graphics Illustrator in designing a cover.

As part of the EMBO publication's Transparent Editorial Process, EMBO reports publishes online a Review Process File (RPF) to accompany accepted manuscripts. This File will be published in conjunction with your paper and will include the referee reports, your point-by-point response and all pertinent correspondence relating to the manuscript.

You are able to opt out of this by letting the editorial office know (emboreports@embo.org). If you do opt out, the Review Process File link will point to the following statement: "No Review Process File is available with this article, as the authors have chosen not to make the review process public in this case."

I look forward to seeing a revised form of your manuscript when it is ready.

Yours sincerely,

Yehu Moran
Academic Editor
EMBO Reports

Referee #1:

This manuscript presents a comparative evolutionary analysis of the molecular biology of GTSF1 in nematodes, a protein that ancestrally acts as an activating factor for PIWI in the metazoa, but which (shown here) evolved to associate with an RdRp involved in endo-siRNA (26G-RNA) biogenesis at the base of the nematoda. The authors show that the new interaction depends on the N-terminal domain of the RdRP, and show experimentally that this is conserved in Clade V nematodes. Using comparative genomic analyses and computational folding predictions they also show that this association arose at the base of extant nematodes, and has been broadly conserved (with multiple losses) since. This will be of interest to anyone working on RNAi function in nematodes, and to those working more broadly on the evolution or comparative biology of RNAi-related pathways. The extreme brevity of my review primarily reflects the high quality of the work and manuscript; I think it's great.

The work seems extremely well done and well presented. Although dense, it was easy to read and interesting. I did not detect any obvious flaws in the experiments or analyses, and given the intentionally focused story, I felt that any requests to extend or expand the work would simply be taking it beyond its intended scope. My only concern is whether this is of broad enough interest for the journal; but on reflection I feel it fits perfectly the remit "...particularly welcomes shorter-format articles framed around a single key finding, backed up by multiple lines of experimental evidence".

Specific minor points:

1. L52 The term 'basal' should not be used like this: https://doi.org/10.1111/j.0307-6970.2004.00262.x

2. L55-56 says 'in nematodes', but since RRF-1 does not occur in anything other than very close relatives of elegans, I think 'in nematode' is too broad.

3. L60-62 "... selective pressures ... are not understood" seems to imply that is a question being addressed in the present manuscript, but it is not

4. L242 please spell out why the comparison is being made with plant RdRps, rather than other metazoan ones (like chelicerates)

5. L300-301 I love the use of nematomorpha for this, and if I were to request any expansion to this work I would ask for more (if there are any) and/or some other ecdysozans that are slightly further out.

6. L311-312 I am slightly concerned that the patchiness of GTSF-1 could be an artefact of high divergence, and that an improved search (e.g. HMM profiles rather than blastp, perhaps followed up by fold prediction) might be more comprehensive?

7. L334-336 Unclear whether the length comparison is being made to suggest a lack of confidence, or to emphasis the variation? Is the short one apparently complete (same length in closer relatives, domains, etc)

8. L443-445 "This framework suggests an evolutionary scenario whereby loss of GTSF-1 binding drives repeated PRG-1 loss in nematodes ..." Drives, or permits? I don't follow this argument. If mutation gave rise to an "... ancestral PRG-1 that could not be productively engaged by GTSF-1 [and was thus] functionally suboptimal ..." then that mutation would not spread and would be lost from the population rapidly, long before anything happened to GTSF 1. Far more likely that the function was no longer needed for some reason. As the next sentence seems to imply, much more likely that PRG-1 took on a new (or additional role) and then binding with GTSF was no longer beneficial.

9. 471-480 I did not find this argument very convincing.

Typo's/Language

1. L7 and line 243 'Comprises' of 'Composed of', not 'comprised of'

2. L10 "RNase H-like enzymes" feels like another element of the list, rather than an explanatory addition

3. L47 Don't start a sentence with 'C.'

4. L102 "...in all three" implies we know which three, but they have not yet been introduced

5. L123 "rescued ... hypersensitivity" seems an odd phrasing. Hyper relative to what, if it is rescuable? In what context?

6. L143 "$\log_{10}$ p-value = 2.83" must be missing a negative, as 10^2.83 is much larger than 1

7. L364-366 I was not sure what the implies take-home was here?

8. L503: what does "adaptive-enabling specialization" mean?

9. L709 - I cannot see what 'set A' and 'set B' are referring to?

10. L843 what is meant by 'biotype' here?

11. Figure 1 "Dotted lines mark deleted genomic regions" there don't seem to be any dotted lines?

Referee #2:

Summary

The manuscript by Govind et al. investigates the evolutionary history of the function of the Gtsf-1 protein in nematodes. Gtsf-1 is a protein known to function in genome defense in the germline of diverse animals by converting target-bound PIWI-clade Argonaute proteins into silencing competent complexes. However, in nematodes, Gtsf-1 does not bind PIWI proteins, but instead the RNA-dependent RNA polymerase RRF-3, which curiously also functions in a small RNA-mediated silencing pathway. This represents an interesting example of a change in protein-protein interaction of an essential protein, which is of broad interest to the field of molecular evolution and of particular interest to researchers in the small RNA biology field, where recent findings have uncovered Gtsf-1 as critical molecular switch able to sense structural rearrangements in PIWI proteins upon target engagement.

In the present paper, the authors combine molecular investigation of Gtsf-1 function in three nematode species with, phylogenomic analysis and structure modeling to investigate the evolutionary origin of this atypical Gtsf-1 protein function. They find that the interaction between Gtsf1 and RRF-3 is deeply conserved in nematodes and identify interaction region in both Gtsf-1 and RRF-3. Furthermore, the authors provide evidence from interaction modelling that the switch from PIWI to RdRP protein interaction by Gtsf-1 likely happened in a common ancestor of all nematodes.

Overall, the paper is well written, the data is properly analysed, and the conclusions are well-supported by the presented data. However, there are specific parts of the manuscript, where I do have (mainly minor) concerns about the data representation, description, or interpretation. Addressing these concerns would be important to improve the clarity and readability of the manuscript.

Major concerns

1. My only major reservation is the insufficient justification and explanation for the use of different structure prediction models (AF2 or AF3) in the paper. In places, it seems that the two models have given conflicting results, which warrant thorough information on the interpretations made and their implications.
Specifically, in Figure 6B / Lines 359-369: Here, the question of GTSF-1 specificity to RRF-3 is addressed by AF2 modeling. The data show that Gtsf-1 likely interacts specifically with RRF-3 and not with PRG-1 or RRF-1/EGO-1. However, these results seem to conflict with the results in Figure 5F-G, where AF3-based modeling predicts similar strong interaction of EGO-1, RRF-1, and RRF-3 with Gtsf-1. Have the authors tried to replicate the striking results in Figure 6B, D with AF3? The authors should revise the manuscript to provide thorough information on the choices made about structure prediction model. Furthermore, the implications of the model choice should be shown by direct comparison of the results and discussed in the text.

Minor concerns

1. Lines 87-88: the text is missing a reference (figure or paper) for the statement "In contrast, C. elegans (Cel), C. briggsae (Cbr), and P. pacificus (Ppa) each possess a single ortholog of GTSF1." Please provide the reference or analysis supporting this statement.
2. Line 118: The authors should add the missing context of what RppH-treated libraries are and how these differ from the other analyzed small RNA libraries.
3. Lines 119-121: It is unclear to me how the authors can conclude from Figure 1H that 22G-RNAs are strongly reduced and piRNAs moderately increased. To me, the box plot indicates that 22G-RNAs are unchanged in P. pac. Gtsf1 mutants.

This makes is unclear if the conclusion "We conclude that the phenotypes we previously described for gtsf-1 loss in C. elegans are conserved in C. briggsae and P. pacificus." (lines 125-126) is supported. The manuscript should be revised to provide a clearer explanation of these data.
4. Lines 207-208: Given that non-interacting flanking residues affect ipTM calculation, it seems plausible that the observed difference in average ipTM could be driven by RRF-3 GID being more structured on average than full-length RRF3. If so, I'm not sure what this analysis shows beyond the reflection of RRF-3 secondary structure.
The same comment applies to Figure 5G where differences in how structured the different RdRPs are could further bias the results. This concern could potentially be addressed by analysis focusing on local protein-protein interaction scoring in the models (e.g. the LIS score or similar).
5. Lines 187-188: The authors write that "The deletion of C-terminus did not affect RRF-3 protein levels (Figure 3F)." The difference in band intensity is, however, not dissimilar to the bands compared in Figure 2C, where the conclusion is the protein abundance did change. To clarify this, band quantification normalized to loading control bands, should be provided.
6. Figure 3G: the results seem to indicate that ERI-9 and ERI-3 interaction is lost in the C-terminal deletion of Gtsf-1. Given that ERI complex formation is a central topic in the paper, this possible interaction loss should be commented on in the text.
7. Lines 236-237: it is hard to see the claimed good superimposition of RRF-3 and AtRDR2 in Figure 4F. The display of the superimposition is very small and no quantification of the superimposition is presented. I recommend to revise the panel for improved visualization of the superimposition and to include a quantification of the similarity (e.g. RMSD or TM-score depending on the level of similarity as already provided for Figure 5F).
8. Lines 242-243: "subdomain 1 of the GID did not show clear structural homology to plant RdRP domains, suggesting it may be nematode-specific". However, this is not supported by Figure 4H, which does not show any model of plant RdRP domains. The manuscript should be revised to adjust the claim or to provide clear support for the it.
9. Line 292: the authors write: "Having established the molecular basis for GTSF-1's selectivity for RRF-3". However, the conclusion in the last sentence of the preceding paragraph was that such selectivity could not presently be resolved. This should be revised for consistency.
10. Lines 345-347: the statement is that T muris is closely related to T muris. Is this what the authors wanted to say?

Addition of non-essential suggestions

a) It would be helpful to define ERI complex in the abstract
b) Line 72: it would be helpful to define RRF-1 and EGO-1 as RdRPs for context
c) Figure 5D: the lower panel is not a loading control per se as it is a separate gel that does not reveal anything about what was loaded in the upper panel. Perhaps reframe this as an GST input control panel?
d) Figure 5F and the connected legend lack information about the species investigated. The information is in the main text, but it would be helpful to also have this in the legend.
e) Proposal: add At RdRPs to the AF2 interaction prediction in Figure 6D to test if GTSF-1 has evolved to bind a conserved part of RdRP
f) Line 370: given the reliance on structure modeling, consider whether 'demonstrate' should be exchanged with a more cautious 'suggest'

Referee #3:

RNA interference pathways are ancient and ubiquitously present in eukaryotes yet are known to diverge fast in evolution to adapt to rapid changes in their target pathogens. These include frequent gene gains and losses. In Govind et al., the authors investigate one extreme example of adaptation where GTSF1, initially identified as a co-factor of PIWI proteins in the piRNA pathway, lost its original function, and instead, facilitates the production of 26G small RNAs through binding the RdRP homolog RRF-3 in C. elegans. The study addresses how and when this functional shift occurred in evolution. They experimentally showed that GTSF1 is also required for the production of 26G small RNAs in another Caenorhabditis worm and a closely related Chromadoria worm, and used the Alphafold-aided protein structure prediction to identify domains and amino acid residues of GTSF1 and RRF-3 that are responsible and conserved for their interaction. Overall, the study offers an in depth insight of how the GTSF1 gene evolved to function with RdRP or PIWI in the RNAi pathways. However, I fear that the study does not reveal novel mechanisms of how RdRP proteins and their co-factors function, hence, its broader biological implications are limited. To improve the clarity and strengthen their claims, I suggest the following modifications before publication.

Major comments:
1. Authors claim that the transition from PIWI-binding to RdRP-binding occurred in the common ancestor of Nematoda. However, this is only supported by a structural prediction of the PRG-1/GTSF1 homologs heterodimer from a single Nematomorpha species. Since this is a key claim of the paper, please substantiate this finding either experimentally (such as in the GST pull down assay or Y2H) or by examining more species outside but related to nematodes.

2. Please explain the confidence window of ipTM and RMSD values in structural predictions. What are the chances of finding false positive interactions? As a sanity check, what happens if you mutate specific residues that mediate the interaction of GTSF1 and RRF3, e.g. the residues that are experimentally tested in the study? Does the Alphafold still predict similar co-structures? If yes, that would expose a limitation of multimer prediction that is important to mention. If not, that would give further confidence in the structure prediction, which is also important to mention.

3. It would be helpful to show the conservation of protein-protein interactions using schematic representation of structures (e.g. marking alpha helices and beta sheets with spirals and arrows) and indicating where the interactions are made, at least for selected key figure panels. Superimposed images do not always capture which parts are overlapping with each other and whether any parts of the structure are missing. Related to this, do CHHC-type and CCHC-type zinc finger motifs of GTSF1 homologs fold in the same way? If they form differently, is it not significant that they are both predicted to contribute to RRF3 interaction?

4. C.elegans EGO-1 and RRF-1a as well as other nematode EGO-1 and RRF-1 homologs lack the key residues for binding GTSF-1 (Extended Figure 3). Yet, the Alphafold predicted co-structures largely retained the configurations (Extended Figure 3). Could you provide more details of how these interactions are enabled?

5. Are the residues responsible for PIWI-binding and RdRP-binding conserved in other Nematoda and Nematomorpha GTSF-1 homologs examined in Figure 6?

Minor comments:
6. Why were different versions of Alphafold used for multimer predictions? Did they give different or very similar results? Please explain the rationale or use one version throughout the study.

7. Please either use "Supplementary" or "Extended" Figures.

8. Numbering is incorrect for CbrRRF-3 "KTKGMLQDQ-C" in Extended Figure 3.

9. Please make the code for the computational analyses available.

Govind et al, 2026

We thank all the reviewers for the valuable time that they took to evaluate our manuscript. Below, we will address all the identified issues, point-by-point. Our replies will be in *Italics*.

Referee #1

This manuscript presents a comparative evolutionary analysis of the molecular biology of GTSF1 in nematodes, a protein that ancestrally acts as an activating factor for PIWI in the metazoa, but which (shown here) evolved to associate with an RdRp involved in endo-siRNA (26G-RNA) biogenesis at the base of the nematoda. The authors show that the new interaction depends on the N-terminal domain of the RdRP, and show experimentally that this is conserved in Clade V nematodes. Using comparative genomic analyses and computational folding predictions they also show that this association arose at the base of extant nematodes, and has been broadly conserved (with multiple losses) since. This will be of interest to anyone working on RNAi function in nematodes, and to those working more broadly on the evolution or comparative biology of RNAi-related pathways. The extreme brevity of my review primarily reflects the high quality of the work and manuscript; I think it's great.
The work seems extremely well done and well presented. Although dense, it was easy to read and interesting. I did not detect any obvious flaws in the experiments or analyses, and given the intentionally focused story, I felt that any requests to extend or expand the work would simply be taking it beyond its intended scope. My only concern is whether this is of broad enough interest for the journal; but on reflection I feel it fits perfectly the remit "...particularly welcomes shorter-format articles framed around a single key finding, backed up by multiple lines of experimental evidence".

Clearly, we are extremely pleased that this reviewer is so positive about our work.

Specific minor points

- L52 The term 'basal' should not be used like this: https://doi.org/10.1111/j.0307-6970.2004.00262.x

  Changed to "In some species of nematodes" (Line 53)

- L55-56 says 'in nematodes', but since RRF-1 does not occur in anything other than very close relatives of elegans, I think 'in nematode' is too broad.

  Changed to "in C. elegans" (Line 57)

- L60-62 "... selective pressures ... are not understood" seems to imply that is a question being addressed in the present manuscript, but it is not.

  Removed. Statement changed to "More fundamentally, the evolutionary origins that redirected C. elegans GTSF-1 from PIWI binding to RRF-3 binding are not understood." (Lines 62-63)

- L242 please spell out why the comparison is being made with plant RdRps, rather than other metazoan ones (like chelicerates)

  We compare the RRF-3 GID to plant RdRPs because plant RdRP structures are currently the only available experimental templates that enable a domain-level structural annotation of the GID. Although other metazoan RdRPs exist, their structures are not experimentally resolved, making plant RdRPs the most informative reference for this specific comparison.

- L300-301 I love the use of nematomorpha for this, and if I were to request any expansion to this work I would ask for more (if there are any) and/or some other ecdysozans that are slightly further out.

  We very much share the reviewer's enthusiasm for this comparison. Unfortunately, extending this analysis is currently not feasible. The only Nematomorpha species with a sequenced genome that has both PIWI and GTSF-1 is Nectonema munidae. Genome sequencing efforts for additional species are underway, but the assemblies are not yet publicly available.

- L311-312 I am slightly concerned that the patchiness of GTSF-1 could be an artefact of high divergence, and that an improved search (e.g. HMM profiles rather than blastp, perhaps followed up by fold prediction) might be more comprehensive?

  This is a thoughtful suggestion. HMMER is a good way to identify proteins that are divergent homologues and can outperform BLAST but this is only likely if there is a clear domain structure within the protein that can be used to build a profile HMM that can be used for a sensitive search. In any case, any such hits would have to be searched for orthology using best reciprocal blast to validate them. If performing GTSF-1 direct searches (without a profile HMM) BLAST and HMMER will have very similar sensitivity because the HMMER tool that would take a protein (C. elegans GTSF1) and search genomes for equivalent proteins (phmmer) is actually working in the same way as blast. They give comparable results for reciprocal blast searches, as observed for PRG-1 in an earlier study (Sarkies et al., 2015).

- L334-336 Unclear whether the length comparison is being made to suggest a lack of confidence, or to emphasis the variation? Is the short one apparently complete (same length in closer relatives, domains, etc)

  We thank the reviewer for flagging this ambiguity. The intent was to highlight the notable variation in GTSF-1 length across species. Regarding completeness: the T. spiralis GTSF-1 (95 amino acids) appears to represent a short but complete protein, retaining the full tandem zinc finger region. We see similar shorter sequences in Paralinhomoeus gsco26 and *P. sambesi and for each of*

these, the predicted Zinc-binding fold is intact. We added this information to lines 366-368.

- L443-445 "This framework suggests an evolutionary scenario whereby loss of GTSF-1 binding drives repeated PRG-1 loss in nematodes ..." Drives, or permits? I don't follow this argument. If mutation gave rise to an "... ancestral PRG-1 that could not be productively engaged by GTSF-1 [and was thus] functionally suboptimal ..." then that mutation would not spread and would be lost from the population rapidly, long before anything happened to GTSF 1. Far more likely that the function was no longer needed for some reason. As the next sentence seems to imply, much more likely that PRG-1 took on a new (or additional role) and then binding with GTSF was no longer beneficial.

  We thank the reviewer for this important point and agree that "drives" is too strong.

  The RNAi pathways in C. elegans are highly plastic, and PRG-1 is embedded in a network of overlapping germline silencing pathways that includes multiple Argonautes and RdRP. Loss or remodeling of PRG-1 function could be buffered by compensatory changes elsewhere, and hence its function might not have been needed in the lineages leading to nematodes. Our AlphaFold models do not show a gross structural alteration in PRG-1 that would abolish GTSF-1 binding, nor do they indicate that nematode PRG-1 has lost the capacity to bind non-nematode GTSF1 proteins. Instead, the models are more consistent with nematode GTSF-1 proteins diverging structurally to act with the RdRP RRF-3 rather than PRG-1 (Figure 6D). However, it is difficult to establish directionality, whether PRG-1 became GTSF-1 independent before GTSF-1 adopted its role with RRF-3, without additional experiments and without knowledge of PRG-1 function outside clade V species. Hence, we agree that a softer statement would be more appropriate here.

  We have now replaced "drives" with "permits".

- 471-480 I did not find this argument very convincing.

  We appreciate the honest assessment. We have reviewed this passage and softened the language to present the argument as speculative. We believe the argument is worth presenting as a hypothesis. (Lines 499-500)

<u>Typo's/Language</u>

- L7 and line 243 'Comprises' of 'Composed of', not 'comprised of'

  Changed to "composed of".

- L10 "RNase H-like enzymes" feels like another element of the list, rather than an explanatory addition

  Changed to "Argonaute/Piwi proteins which are RNase H-like enzymes that utilize these small RNAs…"

- L47 Don't start a sentence with 'C.'

  Changed to "In C. elegans"

- L102 "...in all three" implies we know which three, but they have not yet been introduced

  Changed to "To study this further, we generated gtsf-1 mutant alleles in C. elegans, C. briggsae, and P. pacificus using CRISPR/Cas9."

- L123 "rescued ... hypersensitivity" seems an odd phrasing. Hyper relative to what, if it is rescuable? In what context?

  Added "In C. elegans, loss of the 26G-RNA pathway causes the "Eri" (Enhanced RNAi) phenotype, characterized by hypersensitive silencing of somatic genes via RNAi32,49. This hypersensitivity is observed in gtsf-1 deletion mutants exposed to dpy-13 RNAi, which display an extreme dumpy phenotype31. Expression of C. briggsae GTSF-1 partially rescues this phenotype in C. elegans, again demonstrating functional conservation." (Lines128-132)

- L143 "log_{10} p-value = 2.83" must be missing a negative, as 10^2.83 is much larger than 1

  Changed to -log_{10} p-value = 2.83 (Line 151)

- L364-366 I was not sure what the implies take-home was here?

  We thank the reviewer for pointing out that this passage was unclear. We decided to remove this statement in light of the discrepancy between AF2 and AF3 for GTSF-1 binding to RRF-1/EGO-1.

- L503: what does "adaptive-enabling specialization" mean? pathways

  We missed a comma. The corrected statement is "..may be adaptive, enabling specialization of small RNA.."

- L709 - I cannot see what 'set A' and 'set B' are referring to?

  These are classifiers for samples from two sets of experiments. These experiments were performed over several years and the sample were analyzed

by different people. Hence, we need to describe the methods used for each set separately.

Lines 682:" For GTSF-1 immunoprecipitation in C. briggsae and P. pacificus ***(Set A)"***
Lines 693: "For RRF-3 immunoprecipitation in C. briggsae and RRF-3/GTSF-1 dual immunoprecipitation in C. elegans ***(Set B)"***

- L843 what is meant by 'biotype' here?
  Lines 857: 'biotype' changed to "Aligned reads were assigned to classes (rRNA, miRNA, piRNA, 26G-RNA, and 22G-RNA) using tiny-count"

- Figure 1 "Dotted lines mark deleted genomic regions" there don't seem to be any dotted lines?
  We removed this line as it was an error. Changed to "Gray dotted boxes mark deleted genomic regions"

Referee #2

The manuscript by Govind et al. investigates the evolutionary history of the function of the Gtsf-1 protein in nematodes. Gtsf-1 is a protein known to function in genome defense in the germline of diverse animals by converting target-bound PIWI-clade Argonaute proteins into silencing competent complexes. However, in nematodes, Gtsf-1 does not bind PIWI proteins, but instead the RNA-dependent RNA polymerase RRF-3, which curiously also functions in a small RNA-mediated silencing pathway. This represents an interesting example of a change in protein-protein interaction of an essential protein, which is of broad interest to the field of molecular evolution and of particular interest to researchers in the small RNA biology field, where recent findings have uncovered Gtsf-1 as critical molecular switch able to sense structural rearrangements in PIWI proteins upon target engagement.
In the present paper, the authors combine molecular investigation of Gtsf-1 function in three nematode species with phylogenomic analysis and structure modeling to investigate the evolutionary origin of this atypical Gtsf-1 protein function. They find that the interaction between Gtsf1 and RRF-3 is deeply conserved in nematodes and identify interaction region in both Gtsf-1 and RRF-3. Furthermore, the authors provide evidence from interaction modelling that the switch from PIWI to RdRP protein interaction by Gtsf-1 likely happened in a common ancestor of all nematodes.
Overall, the paper is well written, the data is properly analysed, and the conclusions are well-supported by the presented data. However, there are specific parts of the manuscript, where I do have (mainly minor) concerns about the data representation, description, or interpretation. Addressing these concerns would be important to improve the clarity and readability of the manuscript.

We thank the reviewer for the constructive comments.

<u>Major concerns</u>

My only major reservation is the insufficient justification and explanation for the use of different structure prediction models (AF2 or AF3) in the paper. In places, it seems that the two models have given conflicting results, which warrant thorough information on the interpretations made and their implications.
Specifically, in Figure 6B / Lines 359-369: Here, the question of GTSF-1 specificity to RRF-3 is addressed by AF2 modeling. The data show that Gtsf-1 likely interacts specifically with RRF-3 and not with PRG-1 or RRF-1/EGO-1. However, these results seem to conflict with the results in Figure 5F-G, where AF3-based modeling predicts similar strong interaction of EGO-1, RRF-1, and RRF-3 with Gtsf-1. Have the authors tried to replicate the striking results in Figure 6B, D with AF3? The authors should revise the manuscript to provide thorough information on the choices made about structure prediction model. Furthermore, the implications of the model choice should be shown by direct comparison of the results and discussed in the text.

We thank the reviewer for raising this important methodological concern. We fully agree that this required better clarification, and we have revised the manuscript substantially in response.

*<u>Discrepancy between AF2 and AF3</u>**:** AF2-Multimer predicts a specific interaction between GTSF-1 and RRF-3, but not with EGO-1 or RRF-1, whereas AF3 predicts binding of GTSF-1 to all three RdRPs. This discrepancy likely reflects fundamental differences in how the two methods use coevolutionary information.*

AF2-Multimer relies on paired multiple sequence alignment (MSA) data that encodes inter-chain coevolution and performs best when a clear evolutionary signal supports a specific protein–protein interface (Evans et al., 2021). When that signal is weak or absent, as appears to be the case for the GTSF-1–RRF-1 and GTSF-1–EGO-1 pairs, interface prediction quality deteriorates, reflected in lower confidence scores.

In contrast, AF3 relies less on deep MSA processing and instead generates coordinates via a diffusion model (Abramson et al., 2024). Because RRF-1, RRF-3, and EGO-1 share a highly similar structural fold and partially conserved surface features, AF3 may rely on these similarities and thus predict GTSF-1 binding to all three RdRPs, effectively detecting GTSF-1's specificity for RRF-3, as found experimentally

<u>Changes made:</u>

(1) We have restructured the manuscript so that AlphaFold2-Multimer results are presented in all main figures. AF3 results for GTSF-1–RRF-3 and GTSF-1–RRF-1/EGO-1 predictions in C. elegans, C. briggsae, and P. pacificus are now shown in Extended Figures for comparison.

(2) We have added a new section to the text (Lines 297–306 of the revised manuscript) discussing the differences between AF2 and AF3 and explaining our rationale for using AF2-Multimer as our primary tool in contexts requiring interaction specificity discrimination.

(3) Regarding the AF3 high-throughput comparison: our HT-ColabFold pipeline (Hohmann et al., 2025) is established on AF2-Multimer, and we do not currently have a validated pipeline for AF3 at this scale. However, the pairwise comparisons described above are provided to illustrate the difference, and we believe the mechanistic explanation and the experimental validation data (IP-MS, Y2H, GST pulldown) sufficiently support our conclusions independently of any single modelling approach.

<u>Minor concerns</u>

- Lines 87-88: the text is missing a reference (figure or paper) for the statement "In contrast, C. elegans (Cel), C. briggsae (Cbr), and P. pacificus (Ppa) each possess a single ortholog of GTSF1." Please provide the reference or analysis supporting this statement.

This statement simply results from reciprocal BLAST searches supported by manual inspection of the identified genes. We could not think of a useful way to show this in a figure. We have added a textual statement here." based on reciprocal BLAST search and current annotation" (Lines 105).

- Line 118: The authors should add the missing context of what RppH-treated libraries are and how these differ from the other analyzed small RNA libraries.

  We have added an explanatory sentence in this section. Briefly, RNA 5' Pyrophosphohydrolase (RppH) treatment converts 5'-triphosphorylated RNA to 5'-monophosphate RNA, thereby enabling adapter ligation and sequencing of RNA species that are otherwise excluded from standard small RNA-seq libraries. This enriches for primary small RNA species such as 22G-RNAs which carry a 5'-triphosphate in contrast to 26G-RNAs or 21U-RNAs that bear a 5'-monophosphate and are captured in both library types. (Lines115-117)

- Lines 119-121: It is unclear to me how the authors can conclude from Figure 1H that 22G-RNAs are strongly reduced and piRNAs moderately increased. To me, the box plot indicates that 22G-RNAs are unchanged in P. pac. Gtsf1 mutants. This makes is unclear if the conclusion "We conclude that the phenotypes we previously described for gtsf-1 loss in C. elegans are conserved in C. briggsae and P. pacificus." (lines 125-126) is supported. The manuscript should be revised to provide a clearer explanation of these data.

  We thank the reviewer for this comment. The box plot in Figure 1F displays genome-wide levels of each small RNA class, which may appear unchanged due to the large contribution of reads mapping to non-target loci. The strong depletion of 26G-RNA pathway-dependent 22G-RNAs is only apparent when considering the subset of 22G-RNAs mapping antisense to 26G-RNA target genes (shown in Extended Figure 1C and 1E for C. briggsae). All 22G-RNAs are "secondary" small-RNAs, produced downstream of piRNA and 26G-RNA targeting. Thus, a significant depletion of the specific subset of secondary 22G-RNAs downstream of 26G-RNA target genes shows the strong and specific role of GTSF-1 in the 26G-RNA pathway. We clarify this better in the main text. (Lines117-120).

- Lines 207-208: Given that non-interacting flanking residues affect ipTM calculation, it seems plausible that the observed difference in average ipTM could be driven by RRF-3 GID being more structured on average than full-length RRF3. If so, I'm not sure what this analysis shows beyond the reflection of RRF-3 secondary structure.

  The same comment applies to Figure 5G where differences in how structured the different RdRPs are could further bias the results. This concern could potentially be

addressed by analysis focusing on local protein-protein interaction scoring in the models (e.g. the LIS score or similar).

This is an important methodological concern. The observation that the ipTM increases upon trimming or truncating the constructs has at least two reasons:

A) Trimming non-essential domains and disordered regions eliminates the negative context that suppresses AlphaFold confidence. In full sequences, these segments introduce structural noise via off-target contacts or competing orientations, hindering a high-confidence consensus. Removing these regions simplifies the prediction and typically results in a measurable increase in iPTM. Pre:print (Dunbrack, 2025), (Bret et al., 2024)

B) The ipTM is computed over whole chains, so changing construct boundaries can change the score even if the core interface is essentially the same. (Dunbrack, 2025)

In the revised manuscript, we have removed the sections that relied on the trimmed RRF-3 GID construct for ipTM comparisons, as we concluded these analyses are no longer necessary to support our central claims. Our primary conclusions, that GTSF-1–RRF-3 interaction is predicted with good confidence, is specific to RRF-3 over other RdRPs, and is conserved across nematodes, are now supported by AF2-Multimer predictions using full-length proteins throughout the paper.

Regarding differences in RdRP structural order biasing Figure 5G: to address this, we have included AF2-monomer predictions of all three RdRPs colored by pLDDT score (a per-residue confidence metric) (Extended Figure 3C). This demonstrates that the overall predicted structural confidence is comparable across RRF-1, RRF-3, and EGO-1, including at the residues comprising the GID region. Importantly, our conclusions are also strongly supported by experimental evidence in three species (IP-MS, Y2H, GST pulldown), which provides an independent validation, orthogonal to any modeling concern.

- Lines 187-188: The authors write that "The deletion of C-terminus did not affect RRF-3 protein levels (Figure 3F)." The difference in band intensity is, however, not dissimilar to the bands compared in Figure 2C, where the conclusion is the protein abundance did change. To clarify this, band quantification normalized to loading control bands, should be provided.

  We thank the reviewer for drawing attention to this inconsistency in our interpretation. We have carefully re-examined both blots. In Figure 2C, the RRF-3 band is noticeably reduced while the tubulin loading control remains

comparable between wild-type and gtsf-1 deletion, supporting the conclusion that RRF-3 is destabilized. In Figure 3F, both the RRF-3 band and the tubulin band show comparable reductions in the GTSF-1 C-terminal deletion relative to wild-type, suggesting the reduced intensity reflects a loading difference rather than protein instability. To avoid overinterpreting the band intensity without formal quantification, we have revised the text to state that 'RRF-3 protein remained detectable after the GTSF-1 C-terminus was deleted (Figure 3F)'. (Line 195 and 197)

- Figure 3G: the results seem to indicate that ERI-9 and ERI-3 interaction is lost in the C-terminal deletion of Gtsf-1. Given that ERI complex formation is a central topic in the paper, this possible interaction loss should be commented on in the text.
  Added "Notably, ERI-9 and ERI-3 peptides were not detected in GTSF-1 ΔC samples, suggesting these interactions within the ERIC to be particularly destabilized." (Lines 203-205)

- Lines 236-237: it is hard to see the claimed good superimposition of RRF-3 and AtRDR2 in Figure 4F. The display of the superimposition is very small and no quantification of the superimposition is presented. I recommend to revise the panel for improved visualization of the superimposition and to include a quantification of the similarity (e.g. RMSD or TM-score depending on the level of similarity as already provided for Figure 5F).

  We have substantially revised Figure 4 and Figure 5 in response to this comment. We recolored the structural alignments to improve visual clarity and removed redundant superimpositions that were obscuring the key comparisons. We have also provided RMSD scores for all structural superimpositions, included now in the figure panels and legends.

- Lines 242-243: "subdomain 1 of the GID did not show clear structural homology to plant RdRP domains, suggesting it may be nematode-specific". However, this is not supported by Figure 4H, which does not show any model of plant RdRP domains. The manuscript should be revised to adjust the claim or to provide clear support for the it.

  We have recreated Figure 4G to provide a clear structural superimposition between plant RdRP domains (AtRDR2) and the RRF-3 GID subdomains. Figure 4H now explicitly shows how we designated subdomains 1, 2, and 3 of the RRF-3 GID based on their structural similarity (or lack thereof) to AtRDR2.

- Line 292: the authors write: "Having established the molecular basis for GTSF-1's selectivity for RRF-3". However, the conclusion in the last sentence of the preceding paragraph was that such selectivity could not presently be resolved. This should be revised for consistency.

Thank you for noting the error. We removed this sentence.

- Lines 345-347: the statement is that T muris is closely related to T muris. Is this what the authors wanted to say?

  There was a typo. Now lines 364-366, the sentence intended to state that T. spiralis is closely related to T. muris.

<u>Addition of non-essential suggestions</u>

- It would be helpful to define ERI complex in the abstract.

  Changed to "RdRP complex" for simplicity.
- Line 72: it would be helpful to define RRF-1 and EGO-1 as RdRPs for context.

  We removed reference to RRF-1 and EGO-1 instead in order to simplify the text.
- Figure 5D: the lower panel is not a loading control per se as it is a separate gel that does not reveal anything about what was loaded in the upper panel. Perhaps reframe this as an GST input control panel?

  Changed as suggested. See Figure 5F.
- Figure 5F and the connected legend lack information about the species investigated. The information is in the main text, but it would be helpful to also have this in the legend.

  Changed
- Proposal: add At RdRPs to the AF2 interaction prediction in Figure 6D to test if GTSF-1 has evolved to bind a conserved part of RdRP

  This is a thoughtful suggestion. However, we are skeptical that this would yield high-confidence predictions, given that plant RdRPs lack subdomain 1, which appears important for nematode GTSF-1 binding. Notably, S. ratti RRF-3 also lacks subdomain 1 and, consistent with this, does not show high-confidence predictions in our screening (Extended Data Fig. 4E). Hence, we have not followed this suggestion.
- Line 370: given the reliance on structure modeling, consider whether 'demonstrate' should be exchanged with a more cautious 'suggest'

  Changed to "These findings suggest that nematode GTSF-1 maintains a conserved structural interface with RRF-3 GID, even over substantial evolutionary distances." (Lines 401-402)

Referee #3

RNA interference pathways are ancient and ubiquitously present in eukaryotes yet are known to diverge fast in evolution to adapt to rapid changes in their target pathogens. These include frequent gene gains and losses. In Govind et al., the authors investigate one extreme example of adaptation where GTSF1, initially identified as a co-factor of PIWI proteins in the piRNA pathway, lost its original function, and instead, facilitates the production of 26G small RNAs through binding the RdRP homolog RRF-3 in *C. elegans*. The study addresses how and when this functional shift occurred in evolution. They experimentally showed that GTSF1 is also required for the production of 26G small RNAs in another *Caenorhabditis* worm and a closely related Chromadoria worm, and used the Alphafold-aided protein structure prediction to identify domains and amino acid residues of GTSF1 and RRF-3 that are responsible and conserved for their interaction. Overall, the study offers an in depth insight of how the GTSF1 gene evolved to function with RdRP or PIWI in the RNAi pathways. However, I fear that the study does not reveal novel mechanisms of how RdRP proteins and their co-factors function, hence, its broader biological implications are limited. To improve the clarity and strengthen their claims, I suggest the following modifications before publication.

Major comments:

- Authors claim that the transition from PIWI-binding to RdRP-binding occurred in the common ancestor of Nematoda. However, this is only supported by a structural prediction of the PRG-1/GTSF1 homologs heterodimer from a single Nematomorpha species. Since this is a key claim of the paper, please substantiate this finding either experimentally (such as in the GST pull down assay or Y2H) or by examining more species outside but related to nematodes.

  We understand the reviewer's concern about supporting a key evolutionary claim with data from a single species. Unfortunately, extending this analysis is not currently feasible. Among the four Nematomorpha genomes we examined (Acutogordius australiensis, Nectonema munidae, Gordionus montsenyensis, and Gordius aquaticus), only Nectonema munidae encodes both a PIWI ortholog and a GTSF-1 ortholog. Genome sequencing projects for additional Nematomorpha species are underway, but the assemblies are not yet publicly available for our analysis. Experimental approaches (GST pulldown or Y2H) would require recombinant protein production for N. munidae proteins, which are highly divergent from characterized proteins and would require extensive codon optimization and validation, work that is beyond the scope of this study. We have therefore framed our conclusion more carefully and have explicitly noted this as a limitation in the revised manuscript. (Lines 544 "The limited availability of sequenced genomes prevented us from testing additional Nematomorpha species containing both PIWI and GTSF-1.")

- Please explain the confidence window of ipTM and RMSD values in structural predictions. What are the chances of finding false positive interactions? As a sanity check, what happens if you mutate specific residues that mediate the interaction of GTSF1 and RRF3, e.g. the residues that are experimentally tested in the study? Does the Alphafold still predict similar co-structures? If yes, that would expose a limitation of multimer prediction that is important to mention. If not, that would give further confidence in the structure prediction, which is also important to mention.

  Thank you for these questions about the reliability of our structural predictions. ipTM is an interface confidence metric: scores >0.8 generally indicate a high-confidence predicted interaction, scores <0.6 are usually unreliable, and intermediate values (0.6–0.8) should be interpreted cautiously (Evans et al., 2021) RMSD is a geometric similarity metric that describes how closely two structures superimpose. An RMSD below 1 Å indicates an exceptionally close superposition, while values below 2 Å are generally considered high-quality structural agreement (Kufareva & Abagyan, 2012). Our high-confidence predictions match or exceed these thresholds.

  Regarding the mutagenesis sanity check: we believe our experimental mutagenesis data already provides the most direct validation of the predicted interface. The W39A;R42A substitutions in C. elegans GTSF-1 and the W45S;R48A substitutions in C. briggsae GTSF-1 abolish RRF-3 binding in GST pulldown and Y2H assays, respectively, confirming that the predicted interface is functionally required. Similarly, G291A/M292A mutations in C. briggsae RRF-3 abolish interaction with GTSF-1 in Y2H. Performing the reverse test, asking whether AF2 still predicts the co-structure after in silico mutagenesis of interface residues, is an informative but secondary analysis. We note that AlphaFold is not designed to predict the effect of single amino acid mutations on binding (as noted in this EMBL-EBI training material for AlphaFold), and such in silico mutations frequently fail to disrupt predictions even when experimental binding is abolished. We have therefore not performed this analysis.

- It would be helpful to show the conservation of protein-protein interactions using schematic representation of structures (e.g. marking alpha helices and beta sheets with spirals and arrows) and indicating where the interactions are made, at least for selected key figure panels. Superimposed images do not always capture which parts are overlapping with each other and whether any parts of the structure are missing.

We thank the reviewer for this suggestion. We have improved the visualization of the GTSF-1–RRF-3 interface in Figures 4A, 4C, 5A–C, and in the structural superimpositions. Secondary structure elements are now more clearly indicated in key panels, and contact regions are explicitly highlighted.

- Related to this, do CHHC-type and CCHC-type zinc finger motifs of GTSF1 homologs fold in the same way? If they form differently, is it not significant that they are both predicted to contribute to RRF3 interaction?

  We have modified Extended Figure 4B, which includes the C. elegans N-terminal region in the structural alignment alongside CCHC-type zinc finger proteins. The structural comparison shows that the CCHC zinc finger indeed folds like the canonical CHHC-type zinc finger found in C. elegans GTSF-1.

- *C.elegans* EGO-1 and RRF-1a as well as other nematode EGO-1 and RRF-1 homologs lack the key residues for binding GTSF-1 (Extended Figure 3). Yet, the Alphafold predicted co-structures largely retained the configurations (Extended Figure 3). Could you provide more details of how these interactions are enabled?

  We believe this observation reflects a fundamental limitation of AlphaFold3 rather than a biologically meaningful prediction. As discussed in our response to Referee #2 (Major Comment 1), AF3 relies less on coevolutionary information than AF2-Multimer and can generate high-confidence predictions based on overall structural similarity even when specific interface residues are not conserved. This is precisely why AF3 predicts binding of GTSF-1 to all three RdRPs, while AF2-Multimer, which uses paired MSA coevolution signals, selectively predicts the GTSF-1–RRF-3 interaction. In the revised manuscript, we have shifted all main figure structural predictions to AF2-Multimer, which we consider the more appropriate tool for discriminating between closely related potential interaction partners.

- Are the residues responsible for PIWI-binding and RdRP-binding conserved in other Nematoda and Nematomorpha GTSF-1 homologs examined in Figure 6?

  This is addressed directly in the revised Extended Figure 4A, which presents a multiple sequence alignment of GTSF-1 homologs from across the species examined in Figure 6. The key aromatic residues that mediate PIWI binding in mouse and fly GTSF1 orthologs are conserved in Nectonema munidae (Nematomorpha) GTSF-1 but are largely absent from nematode GTSF-1 orthologs. Conversely, one of the RRF-3-binding residues (corresponding to R42 in C. elegans) is conserved across nematode GTSF-1 but are not found in N. munidae.

Minor comments:

- Why were different versions of Alphafold used for multimer predictions? Did they give different or very similar results? Please explain the rationale or use one version throughout the study.

  Please see our response to reviewer 2's major comment.

- Please either use "Supplementary" or "Extended" Figures.

  We apologize for the confusion. EMBO Reports presents non-main figures as 'Expanded View Figures'. We have changed the original non-main figures, both "Supplementary Figure 1" and "Extended Figures 1-4" as "Expanded View Figures 1-5", mentioned in the manuscript as "Fig EV1 to Fig EV5".

- Numbering is incorrect for CbrRRF-3 "KTKGMLQDQ-C" in Extended Figure 3.

  Thank you for noting this error. It has been corrected.

- Please make the code for the computational analyses available.

  Small RNA sequencing data were analyzed using the publicly available tinyRNA pipeline https://github.com/MontgomeryLab/tinyRNA (Tate et al., 2023). Downstream statistical analyses and visualization were performed in RStudio using standard packages (DESeq2, ggplot2). All custom analysis scripts used in this study have been deposited within Source Data. We have added this information to the methods section.

References

Bret, H., Gao, J., Zea, D. J., Andreani, J., & Guerois, R. (2024). From interaction networks to interfaces, scanning intrinsically disordered regions using AlphaFold2. Nature Communications, 15(1). https://doi.org/10.1038/S41467-023-44288-7

Dunbrack, R. L. (2025). Rēs [Formula: see text] loquunt: What's wrong with AlphaFold's [Formula: see text] score and how to fix it. BioRxiv : The Preprint Server for Biology. https://doi.org/10.1101/2025.02.10.637595

Evans, R., O'Neill, M., Pritzel, A., Antropova, N., Senior, A., Green, T., Žídek, A., Bates, R., Blackwell, S., Yim, J., Ronneberger, O., Bodenstein, S., Zielinski, M., Bridgland, A., Potapenko, A., Cowie, A., Tunyasuvunakool, K., Jain, R., Clancy, E., … Hassabis, D. (2021). Protein complex prediction with AlphaFold-Multimer. BioRxiv, 2021.10.04.463034. https://doi.org/10.1101/2021.10.04.463034

Hohmann, U., Graf, M., Tirián, L., Pacheco-Fiallos, B., Schellhaas, U., Fin, L., Handler, D., Phillips, A. W., Riabov-Bassat, D., Faraway, R., Pühringer, T., Szalay, M. F., Roitinger, E., Brennecke, J., & Plaschka, C. (2025). An ATP-gated molecular switch orchestrates human mRNA export. Nature 2025 649:8098, 649(8098), 1042–1050. https://doi.org/10.1038/s41586-025-09832-z

Kufareva, I., & Abagyan, R. (2012). Methods of protein structure comparison. Methods in Molecular Biology (Clifton, N.J.), 857, 231. https://doi.org/10.1007/978-1-61779-588-6_10

Sarkies, P., Selkirk, M. E., Jones, J. T., Blok, V., Boothby, T., Goldstein, B., Hanelt, B., Ardila-Garcia, A., Fast, N. M., Schiffer, P. M., Kraus, C., Taylor, M. J., Koutsovoulos, G., Blaxter, M. L., & Miska, E. A. (2015). Ancient and Novel Small RNA Pathways Compensate for the Loss of piRNAs in Multiple Independent Nematode Lineages. PLOS Biol, 13(2), e1002061. https://doi.org/10.1371/journal.pbio.1002061

Tate, A. J., Brown, K. C., & Montgomery, T. A. (2023). tiny-count: a counting tool for hierarchical classification and quantification of small RNA-seq reads with single-nucleotide precision. Bioinformatics Advances, 3(1). https://doi.org/10.1093/BIOADV/VBAD065

Dear Prof. Ketting

Thank you for the submission of your revised manuscript to our offices. We have now received the enclosed reports from the referees that were asked to assess it. As you will see, Referee #3 still has some minor suggestions that I would like you to incorporate before we can proceed with the official acceptance of your manuscript. Moreover, I also enclose a few technical requirements raised by our in house editorial assistance team that are necessary for the acceptance of your paper for publication in EMBO Reports.

I look forward to seeing a new revised version of your manuscript at your earliest convenience.

Yehu Moran
Academic Editor
EMBO Reports

Specific comments from editorial assistance team

Data availability statement: included, but needs to be renamed to Data Availability

Conflict of interest statement: missing, please provide.

Autor Contributions/CRediT: needs to be removed from the manuscript text and provided only via the submission system.

REFERENCES: Needs to follow our formatting - et al needs to be used after 10 author names; DOIs should only be used for preprints and datasets that have not been published yet.

Point-by-point response: please make sure to include and address new comments from the referees but not the comments of the editorial assistance team.

FUNDING INFO: OK, unless NIH Office of Research Infrastructure Programs (P40 OD010440) also needs to be entered in the system. Please check.

FIGURE CALLOUTS: "Extended Data", "Supplemental Tables" and "Supplementary" should not be used; missing a callout for Suppl. Table 5 and 7. Please correct.

DATASET EV LEGENDS: Excel tables with too many data, rows, columns and sheets are datasets supplemental/supplementary tables or extended data should not be used; Supplementary Tables (1-7) could be updated to Dataset EV1 and each sheet/table could be called Dataset EV1a, Dataset EV1b, Dataset EV1c, etc. Extended Data 1-3 can be Dataset EV2 and each tab/sheet can be Dataset EV2a, etc.; same goes for Extended Data 4-7, Extended Data 8-10, etc. so that we have 4 datasets in the end; alternatively, each sheet in each Excel file could be upldd as a separate file so that we have 17 datasets (each legend would then be provided as a separate sheet/tab in each Excel file)

SYNOPSIS IMAGE: missing, please provide.

SYNOPSIS TEXT: missing, please provide.

R&T TABLE: currently in the manuscript file. It needs to be removed and uploaded separately; callouts to the tables will need correction once the table nomenclature has been sorted out.

SOURCE DATA (SD): SD provided with completed checklist included but SD needs to be uploaded as one zip folder per figure, e.g. Figure 1 Source Data, Figure 2 Source Data, etc. instead of having 2-3 folders zipped in one (Figure Source Data_Fig_1-3); SD for EV figures should be provided in the same way (not in the folders of main figures).

EXTRA NOTES:

- The manuscript sections should be in the following order: Title page - Abstract - Introduction - Results - Discussion - Methods - Data Availability - Acknowledgments - Disclosure Statement & Competing Interests - References - Figure Legends - (Main Tables with legends if applicable) - Expanded View Figure Legends.
- EV figure legends have incorrect nomenclature: should be Figure EV1, etc. instead of Extended Version Figure 1, etc.
- AI Statement and Blinding statement should be in Methods.

*Please note that the specific URLs for PRJEB105295, PXD072041, MSV000100223, MSV000100222, MSV000100221

datasets are not provided in the data availability statement. Please correct.

Figure Legends - Comments
- Please indicate the statistical test used for data analysis in the legends of figures 1F-H; 2A, B, D; 3G, EV1 C-E
- Please note that the box plots need to be defined in terms of minima, maxima, centre, bounds of box and whiskers, and percentile in the legends of figures 1E-H; 3E, 5D, EV1 C, E; EV2 B, D
- Please note that information related to n is missing in the legends of figures 4B, EV2 B, EV3 C, EV4 E. Please provide.

Specific comments from Referees
Referee #1:

I am happy that all of my previous points have either been addressed adequately, or I am willing to accept the authors' arguments

Referee #2:

The authors have fully addressed all my comments as well as the comments from the other reviewers. I particularly appreciate the care the authors have taken to explain non-trivial analysis choices (HMMER vs BLAST, AF2 vs AF3, RMSD interpretation and evo inference limitations). I therefore fully support the publication of the revised manuscript.

Referee #3:

I am satisfied with the authors' responses and the improvements made in the revised manuscript. In my view, the manuscript is ready for publication without additional experiments. My remaining concern relates to the description and interpretation of the AlphaFold structural predictions.

In the Results section entitled "RRF-1 and EGO-1 Possess GID-like Domains but Are Not Predicted to Interact with GTSF-1 by AF2-Multimer," the authors suggest that the discrepancy between AF2-Multimer and AF3 may reflect differences in how the two models use coevolutionary information. I agree that AF2-Multimer better reproduces the experimentally established preference of GTSF-1 for RRF-3 in this system, and I do not question the authors' decision to use AF2-Multimer for the broader comparative analysis.

My concern is more specific: agreement with the experimentally shown biology does not by itself establish the mechanistic reason for the difference between the two prediction methods. The authors show that RRF-3 residues contributing to GTSF-1 binding are conserved among RRF-3 orthologues but are not conserved in RRF-1 and EGO-1 orthologues. However, to support the proposed explanation of the AF2-AF3 discrepancy, it would be necessary to examine the GTSF-1-RRF-1 and GTSF-1-EGO-1 interfaces actually generated by AF3. In particular, it would be informative to identify which RRF-1 and EGO-1 residues mediate the predicted contacts, whether AF3 uses positions equivalent to the validated RRF-3 interface, and whether these alternative contacts and interaction geometries are conserved across orthologues.

Without such analysis, the proposed explanation involving differential use of coevolutionary information remains untested. I therefore suggest either supporting this mechanistic interpretation with the relevant computational analysis or, more simply, describing the different model outputs without assigning a specific cause and acknowledging that the basis of the AF2-AF3 discrepancy remains uncertain. This point does not affect the overall conclusions of the manuscript.

In addition, the response letter states that ipTM scores above 0.8 generally indicate a high-confidence predicted interaction, whereas intermediate values of 0.6-0.8 should be interpreted cautiously (Evans et al., 2021). Many of the GTSF-1-RdRP predictions presented in Figure 6 fall within this intermediate range, and some appear to fall below it. I do not think this invalidates the authors' evolutionary argument, because the overall comparative pattern is highly consistent across distantly related nematode species and is supported by experimental results in the species examined directly. Nevertheless, it would be more accurate to state explicitly that the individual predicted interactions should be interpreted with caution and that the principal strength of Figure 6 lies in the recurring overall pattern rather than in uniformly high confidence for each individual complex.

Please find our replies to the remaining issues below, marked in grey.

*****Specific comments from Referees*****
Referee #1:

I am happy that all of my previous points have either been addressed adequately, or I am willing to accept the authors' arguments

Thank you for the efforts in re-review. We are happy this reviewer is now satisfied.

Referee #2:

The authors have fully addressed all my comments as well as the comments from the other reviewers. I particularly appreciate the care the authors have taken to explain non-trivial analysis choices (HMMER vs BLAST, AF2 vs AF3, RMSD interpretation and evo inference limitations). I therefore fully support the publication of the revised manuscript.

Thank you for the efforts in re-review and appreciate the compliments. We are happy this reviewer is now satisfied.

Referee #3:

I am satisfied with the authors' responses and the improvements made in the revised manuscript. In my view, the manuscript is ready for publication without additional experiments. My remaining concern relates to the description and interpretation of the AlphaFold structural predictions.

In the Results section entitled "RRF-1 and EGO-1 Possess GID-like Domains but Are Not Predicted to Interact with GTSF-1 by AF2-Multimer," the authors suggest that the discrepancy between AF2-Multimer and AF3 may reflect differences in how the two models use coevolutionary information. I agree that AF2-Multimer better reproduces the experimentally established preference of GTSF-1 for RRF-3 in this system, and I do not question the authors' decision to use AF2-Multimer for the broader comparative analysis.

My concern is more specific: agreement with the experimentally shown biology does not by itself establish the mechanistic reason for the difference between the two prediction methods. The authors show that RRF-3 residues contributing to GTSF-1 binding are conserved among RRF-3 orthologues but are not conserved in RRF-1 and EGO-1 orthologues. However, to support the proposed explanation of the AF2-AF3 discrepancy, it would be necessary to examine the GTSF-1-RRF-1 and GTSF-1-EGO-1 interfaces actually generated by AF3. In particular, it would be informative to identify which RRF-1 and EGO-1 residues mediate the predicted contacts, whether AF3 uses positions equivalent to the validated RRF-3 interface, and whether these alternative contacts and interaction geometries are conserved across orthologues.

Without such analysis, the proposed explanation involving differential use of coevolutionary information remains untested. I therefore suggest either supporting this mechanistic interpretation with the relevant computational analysis or, more simply, describing the different model outputs without assigning a specific cause and acknowledging that the basis of the AF2-AF3 discrepancy remains uncertain. This point does not affect the overall conclusions of the manuscript.

We agree that our proposed explanation for the observed differences between AF2 and AF3 are not tested. Since we wish to not delve into this analysis for this study, we have added on line 354: “However, these explanations remain untested and will require further validation.”

In addition, the response letter states that ipTM scores above 0.8 generally indicate a high-confidence predicted interaction, whereas intermediate values of 0.6-0.8 should be interpreted cautiously (Evans et al., 2021). Many of the GTSF-1-RdRP predictions presented in Figure 6 fall within this intermediate range, and some appear to fall below it. I do not think this invalidates the authors' evolutionary argument, because the overall comparative pattern is highly consistent across distantly related nematode species and is supported by experimental results in the species examined directly. Nevertheless, it would be more accurate to state explicitly that the individual predicted interactions should be interpreted with caution and that the principal strength of Figure 6 lies in the recurring overall pattern rather than in uniformly high confidence for each individual complex.

Thank you for noting this. We have added this information within in Lines 438 to 441:" Individual predicted interactions often carried intermediate confidence scores (0.6–0.8), so complex-by-complex comparisons should be interpreted with caution. The strength of this analysis, however, lies not in the confidence of any single prediction but in the consistency of the interaction pattern across species:"

Prof. Rene Ketting
Institute of Molecular Biology
Biology of non-coding RNA
Ackermannweg 4
Mainz 55128
Germany

Dear Rene,

I am very pleased to accept your manuscript for publication in the next available issue of EMBO reports. Thank you for your contribution to our journal.

Your manuscript will be processed for publication by EMBO Press. It will be copy edited and you will receive page proofs prior to publication. Please note that you will be contacted by Springer Nature Author Services to complete licensing and payment information.

You may qualify for financial assistance for your publication charges - either via a Springer Nature fully open access agreement or an EMBO initiative. Check your eligibility: https://link.springer.com/journal/44319/how-to-publish-with-us

Should you be planning a Press Release on your article, please notify the production team via this form in order to coordinate publication and release dates: https://forms.cloud.microsoft/e/eH4A8JVP6g

If you have any questions, please do not hesitate to contact the Editorial Office. Thank you for your contribution to EMBO Reports.

Best regards,
Esther

Esther Schnapp, PhD
Senior Editor
EMBO reports

>>> Please note that it is EMBO Reports policy for the transcript of the editorial process (containing referee reports and your response letter) to be published as an online supplement to each paper. If you do NOT want this, you will need to inform the Editorial Office via email immediately. More information is available here: https://link.springer.com/partners/embo-press/editorial-policies#Peer%20review

EMBO Press Author Checklist

Corresponding Author Name: Rene F. Ketting	
Journal Submitted to: EMBO Reports	
Manuscript Number: EMBOR-2026-63679	

USEFUL LINKS FOR COMPLETING THIS FORM
The EMBO Journal - Author Guidelines
EMBO Reports - Author Guidelines
Molecular Systems Biology - Author Guidelines
EMBO Molecular Medicine - Author Guidelines

Reporting Checklist for Life Science Articles (updated January
This checklist is adapted from Materials Design Analysis Reporting (MDAR) Checklist for Authors. MDAR establishes a minimum set of requirements in transparent reporting in the life sciences (see Statement of Task: 10.31222/osf.io/9sm4x). Please follow the journal's guidelines in preparing your
Please note that a copy of this checklist will be published alongside your article.

Abridged guidelines for figures
1. Data
The data shown in figures should satisfy the following conditions:
- the data were obtained and processed according to the field's best practice and are presented to reflect the results of the experiments in an accurate and unbiased manner.
- ideally, figure panels should include only measurements that are directly comparable to each other and obtained with the same assay.
- plots include clearly labeled error bars for independent experiments and sample sizes. Unless justified, error bars should not be shown for technical
- if n<5, the individual data points from each experiment should be plotted. Any statistical test employed should be justified.
- Source Data should be included to report the data underlying figures according to the guidelines set out in the authorship guidelines on Data

2. Captions
Each figure caption should contain the following information, for each panel where they are relevant:
- a specification of the experimental system investigated (eg cell line, species name).
- the assay(s) and method(s) used to carry out the reported observations and measurements.
- an explicit mention of the biological and chemical entity(ies) that are being measured.
- an explicit mention of the biological and chemical entity(ies) that are altered/varied/perturbed in a controlled manner.
- the exact sample size (n) for each experimental group/condition, given as a number, not a range;
- a description of the sample collection allowing the reader to understand whether the samples represent technical or biological replicates (including how many animals, litters, cultures, etc.).
- a statement of how many times the experiment shown was independently replicated in the laboratory.
- definitions of statistical methods and measures:
  - common tests, such as t-test (please specify whether paired vs. unpaired), simple χ2 tests, Wilcoxon and Mann-Whitney tests, can be unambiguously identified by name only, but more complex techniques should be described in the methods section;
  - are tests one-sided or two-sided?
  - are there adjustments for multiple comparisons?
  - exact statistical test results, e.g., P values = x but not P values < x;
  - definition of 'center values' as median or average;
  - definition of error bars as s.d. or s.e.m.

Please complete ALL of the questions below.
Select "Not Applicable" only when the requested information is not relevant for your study.

Materials

Newly Created Materials	Information included in the manuscript?	In which section is the information available? (Reagents and Tools Table, Materials and Methods, Figures, Data Availability Section)
New materials and reagents need to be available; do any restrictions apply?	Yes	Materials and Methods

Antibodies	Information included in the manuscript?	In which section is the information available? (Reagents and Tools Table, Materials and Methods, Figures, Data Availability Section)
For **antibodies** provide the following information:<br>- Commercial antibodies: RRID (if possible) or supplier name, catalogue number and or/clone number<br>- Non-commercial: RRID or citation	Yes	Materials and Methods

DNA and RNA sequences	Information included in the manuscript?	In which section is the information available? (Reagents and Tools Table, Materials and Methods, Figures, Data Availability Section)
**Short novel DNA or RNA including primers, probes**: provide the sequences.	Yes	Materials and Methods

Cell materials	Information included in the manuscript?	In which section is the information available? (Reagents and Tools Table, Materials and Methods, Figures, Data Availability Section)
**Cell lines:** Provide species information, strain. Provide accession number in repository **OR** supplier name, catalog number, clone number, and/**OR** RRID.	Not Applicable	
**Primary cultures:** Provide species, strain, sex of origin, genetic modification status.	Not Applicable	
Report if the cell lines were recently **authenticated** (e.g., by STR profiling) and tested for mycoplasma contamination.	Not Applicable	

Experimental animals	Information included in the manuscript?	In which section is the information available? (Reagents and Tools Table, Materials and Methods, Figures, Data Availability Section)
**Laboratory animals or Model organisms:** Provide species, strain, sex, age, genetic modification status. Provide accession number in repository **OR** supplier name, catalog number, clone number, **OR** RRID.	Yes	Materials and Methods
**Animal observed in or captured from the field:** Provide species, sex, and age where possible.	Not Applicable	
Please detail **housing and husbandry conditions**.	Yes	Materials and Methods

Plants and microbes	Information included in the manuscript?	In which section is the information available? (Reagents and Tools Table, Materials and Methods, Figures, Data Availability Section)
**Plants:** provide species and strain, ecotype and cultivar where relevant, unique accession number if available, and source (including location for collected wild specimens).	Not Applicable	
**Microbes:** provide species and strain, unique accession number if available, and source.	Not Applicable	

Human research participants	Information included in the manuscript?	In which section is the information available? (Reagents and Tools Table, Materials and Methods, Figures, Data Availability Section)
If collected and within the bounds of privacy constraints report on age, sex and gender or ethnicity for all study participants.	Not Applicable	

Core facilities	Information included in the manuscript?	In which section is the information available? (Reagents and Tools Table, Materials and Methods, Figures, Data Availability Section)
If your work benefited from core facilities, was their service mentioned in the acknowledgments section?	Yes	Acknowledgements

Design

Study protocol	Information included in the manuscript?	In which section is the information available? (Reagents and Tools Table, Materials and Methods, Figures, Data Availability Section)
If study protocol has been **pre-registered, provide DOI in the manuscript**. For clinical trials, provide the trial registration number **OR** cite DOI.	Yes	Materials and Methods
Report the **clinical trial registration number** (at ClinicalTrials.gov or equivalent), where applicable.	Not Applicable	

Laboratory protocol	Information included in the manuscript?	In which section is the information available? (Reagents and Tools Table, Materials and Methods, Figures, Data Availability Section)
Provide DOI OR other citation details if **external detailed step-by-step protocols** are available.	Not Applicable	

Experimental study design and statistics	Information included in the manuscript?	In which section is the information available? (Reagents and Tools Table, Materials and Methods, Figures, Data Availability Section)
Include a statement about **sample size** estimate even if no statistical methods were used.	Yes	Figures
Were any steps taken to minimize the effects of subjective bias when allocating animals/samples to treatment (e.g. **randomization procedure**)? If yes, have they been described?	Not Applicable	
Include a statement about **blinding** even if no blinding was done.	Yes	Statement on Blinding
Describe **inclusion/exclusion criteria** if samples or animals were excluded from the analysis. Were the criteria pre-established?<br><br>If sample or data points were omitted from analysis, report if this was due to attrition or intentional exclusion and provide justification.	Not Applicable	
For every figure, are **statistical tests** justified as appropriate? Do the data meet the assumptions of the tests (e.g., normal distribution)? Describe any methods used to assess it. Is there an estimate of variation within each group of data? Is the variance similar between the groups that are being statistically compared?	Yes	Figure legends and Materials and Methods

Sample definition and in-laboratory replication	Information included in the manuscript?	In which section is the information available? (Reagents and Tools Table, Materials and Methods, Figures, Data Availability Section)
In the figure legends: state number of times the experiment was **replicated** in laboratory.	Yes	Figure legends
In the figure legends: define whether data describe **technical or biological replicates**.	Yes	Figure legends

Ethics

Ethics	Information included in the manuscript?	In which section is the information available? (Reagents and Tools Table, Materials and Methods, Figures, Data Availability Section)
Studies involving **human participants**: State details of **authority granting ethics approval** (IRB or equivalent committee(s), provide reference number for approval.	Not Applicable	
Studies involving **human participants**: Include a statement confirming that **informed consent** was obtained from all subjects and that the experiments conformed to the principles set out in the WMA Declaration of Helsinki and the Department of Health and Human Services Belmont Report.	Not Applicable	
Studies involving **human participants:** For publication of **patient photos**, include a statement confirming that consent to publish was obtained.	Not Applicable	
Studies involving experimental **animals**: State details of **authority granting ethics approval** (IRB or equivalent committee(s), provide reference number for approval. Include a statement of compliance with ethical regulations.	Not Applicable	
Studies involving **specimen and field samples:** State if relevant **permits** obtained, provide details of authority approving study; if none were required, explain why.	Not Applicable	

Dual Use Research of Concern (DURC)	Information included in the manuscript?	In which section is the information available? (Reagents and Tools Table, Materials and Methods, Figures, Data Availability Section)
Could your study fall under dual use research restrictions? Please check biosecurity documents and list of **select agents and toxins** (CDC): https://www.selectagents.gov/sat/list.htm	Not Applicable	
If you used a select agent, is the security level of the lab appropriate and reported in the manuscript?	Not Applicable	
If a study is subject to dual use research of concern regulations, is the name of the **authority granting approval and reference number** for the regulatory approval provided in the manuscript?	Not Applicable	

Reporting

The MDAR framework recommends adoption of discipline-specific guidelines, established and endorsed through community initiatives. Journals have their own policy about requiring specific guidelines and recommendations to complement MDAR.

Adherence to community standards	Information included in the manuscript?	In which section is the information available? (Reagents and Tools Table, Materials and Methods, Figures, Data Availability Section)
State if relevant guidelines or checklists (e.g., **ICMJE, MIBBI, ARRIVE, PRISMA**) have been followed or provided.	Not Applicable	
For **tumor marker prognostic studies,** we recommend that you follow the **REMARK** reporting guidelines (see link list at top right). See author guidelines, under 'Reporting Guidelines'. Please confirm you have followed these guidelines.	Not Applicable	
For **phase II and III randomized controlled trials**, please refer to the **CONSORT** flow diagram (see link list at top right) and submit the CONSORT checklist (see link list at top right) with your submission. See author guidelines, under 'Reporting Guidelines'. Please confirm you have submitted this list.	Not Applicable	

Data Availability

Data availability	Information included in the manuscript?	In which section is the information available? (Reagents and Tools Table, Materials and Methods, Figures, Data Availability Section)
Have **primary datasets** been deposited according to the journal's guidelines (see 'Data Deposition' section) and the respective accession numbers provided in the Data Availability Section?	Yes	Data Availability Section
Were **human clinical and genomic datasets** deposited in a public access-controlled repository in accordance to ethical obligations to the patients and to the applicable consent agreement?	Not Applicable	
Are **computational models** that are central and integral to a study available without restrictions in a machine-readable form? Were the relevant accession numbers or links provided?	Yes	Materials and Methods and Source Data
If publicly available data were reused, provide the respective **data citations in the reference list**.	Not Applicable	
