# Supplementary material for: An evolutionarily conserved N-terminal domain of RRF-3 governs GTSF-1 binding in nematodes
Source: EMBO Rep. 2026 Aug 29;27(19):5530–60. doi: 10.1038/s44319-026-00914-1 (PMC13650160; doi:10.1038/s44319-026-00914-1)
Supplement: Supplementary file 3 [file 44319_2026_914_MOESM10_ESM.zip › Figure 5 Source Data/5H/fold_2025_08_22_celgtsf1vrrf3w2xzn_model_0_ATOMS_section_With_ConSurf/consurf_colored_seq.pdf]

ConSurf Results for job:CelGTSF1_struc date:07/10/2025

```
1           11          21          31          41
MASGESEAPG  TSKNDRFSIT  CPYNSDHKVS  MEEFNTHLWK  CRTEKLHFYP
eeeeeeeeee  eeeeeeebbe  beeeeeeebe  beebeeebbe  beeeebeebe
                        sff   f         f f     sf

51          61          71          81          91
HSLKLKRCSY  NMRHFLPEEE  LQFHEIFCKR  QSADLRRQLS  LEPLELNVAE
eebebeebee  ebeebbeeee  bebeebbbee  eeeeeeeeee  eeebebebee
      fs    f  f  f  f     ff  s

101         111         121         131         141
HLAAQKLRKE  YEKDEESLDG  SDDSDEDEEE  KNLSVTSEIE  KSDVEEVEMM
beeeeeeeee  eeeeeeeeee  eeeeeeeeee  eeeeeebebe  eeeeeeeeee
                                       f  s       f  f

151         161
LETINRLAYL  EMKNDNLIL
eebbeebebb  ebeeeeeee
     f
```

The conservation scale:

1	2	3	4	5	6	7	8	9

Variable **Average** **Conserved**

e - **An exposed residue according to the neural network algorithm.**
b - **A buried residue according to the neural network algorithm.**
f - **A predicted functional residue (highly conserved and exposed).**
s - **A predicted structural residue (highly conserved and buried).**
