# Supplementary material for: An evolutionarily conserved N-terminal domain of RRF-3 governs GTSF-1 binding in nematodes
Source: EMBO Rep. 2026 Aug 29;27(19):5530–60. doi: 10.1038/s44319-026-00914-1 (PMC13650160; doi:10.1038/s44319-026-00914-1)
Supplement: Supplementary file 3 [file 44319_2026_914_MOESM10_ESM.zip › Figure 5 Source Data/5H/fold_2025_08_22_celgtsf1vrrf3w2xzn_model_0_ATOMS_section_With_ConSurf/consurf_colored_seq_CBS.pdf]

ConSurf Results for job:CelGTSF1_struc date:07/10/2025

```
1          11         21         31         41
MASGESEAPG TSKNDRFSIT CPYNSDHKVS MEEFNTHLWK CRTEKLHFYP
eeeeeeeeee eeeeeeebbe beeeeeeebe beebeeebbe beeeebeebe
                      sff   f        f f    sf

51         61         71         81         91
HSLKLKRCSY NMRHFLPEEE LQFHEIFCKR QSADLRRQLS LEPLELNVAE
eebebeebee ebeebbeeee bebeebbbee eeeeeeeeee eeebebebee
      fs   f  f  f  f    ff  s

101        111        121        131        141
HLAAQKLRKE YEKDEESLDG SDDSDEDEEE KNLSVTSEIE KSDVEEVEMM
beeeeeeeee eeeeeeeeee eeeeeeeeee eeeeeebebe eeeeeeeeee
                                    f  s       f  f

151        161
LETINRLAYL EMKNDNLIL
eebbeebebb ebeeeeeee
     f
```

The conservation scale:

1 2 3 4 5 6 7 8 9

Variable — Average — Conserved

e - An exposed residue according to the neural network algorithm.
b - A buried residue according to the neural network algorithm.
f - A predicted functional residue (highly conserved and exposed).
s - A predicted structural residue (highly conserved and buried).
