# Supplementary material for: An evolutionarily conserved N-terminal domain of RRF-3 governs GTSF-1 binding in nematodes
Source: EMBO Rep. 2026 Aug 29;27(19):5530–60. doi: 10.1038/s44319-026-00914-1 (PMC13650160; doi:10.1038/s44319-026-00914-1)
Supplement: Supplementary file 6 [file 44319_2026_914_MOESM13_ESM.zip › Figure EV2 Source Data/EV2F/Cel_rrf3_fold_2025_08_22_celgtsf1vrrf3w2xzn_model_0_ATOMS_section_With_ConSurf/consurf_colored_seq_CBS.pdf]

ConSurf Results for job:CelRRF3 date:08/10/2025

```
1           11          21          31          41
MLPFDNDDSS  DDATTSVRPK  HPRGVPQSQS  TFPRGRSNFS  SGTLPNRKTE
eeeeeeeeee  eebbebbeee  eeeeeeeeee  ebeebeeebe  eeebeeeeee
         f                ff     f       fff f  fff f f  f

51          61          71          81          91
CTPVNTLTIG  HSNKMLLTTF  RMDRNSKSKS  EVDVQEQPVH  SSSSAFPGNH
eeebbbbbee  ebeeebbebb  ebeeeeeeee  eeeeeeeeee  eeeeeeeeee
  fsss      fs f           f

101         111         121         131         141
LNNFSYPVNR  GYLRDYLLQS  QRPSTSKPVD  CSVLKRHSLP  STHILYEKTK
beebebebee  bebeebebee  eeeeeeeeee  eeebeeeebe  bbbbbbeeee
      f  f   f  f                       ff f f  ssss

151         161         171         181         191
HRGGVNIEEQ  EKLVRMLWAA  AEESETVAKT  RQFSKKQAIE  LNFDAKLIGS
eeeeeeeeee  eebbebbebe  beeeeeeeee  eeebeeeebe  bebebebbbb
        ff

201         211         221         231         241
MNNDCFGYCR  AHMENIKDVL  KTHLKLSKVD  EVNWIKVGMV  PRAAYEDKSY
beeebbbbbe  bebeebeebb  eeebeeeebe  ebebeebeeb  eebeeeeebb
                  f           f                 f s ff

251         261         271         281         291
VIDAHLVLTP  NGEVEDENEL  FSEFASSFTS  RITGMLHDQV  FLEVPKMHTL
bbebbbbbee  eeeeeeeeeb  bbebbeebee  ebeebbeeeb  beebeebeeb
 s                                         ff     f  f

301         311         321         331         341
FTKITPQHMD  INISAIAIGN  CPNSGLFLVR  GDFISQENTV  CSVKLQSHHN
beebbeeebe  bbbbbbbbbe  beeebbbbbe  bbbbeeeeee  bebeeeeeee
s      f            sf  s f s s  f  s      f     f       f

351         361         371         381         391
ADASRENSSF  KVAGSNKYLS  YARFEHDKRL  AVVYFGVRLA  EFADDGLDHA
eebeeeeebb  ebeeeeebbb  bbebeeeeeb  bbbbbbbebe  ebeeebbebb
 f          f  f     s     sfff f       s           fss

401         411         421         431         441
GFRLNLYYNL  FVRIVVDMSH  ETTNSIYIQM  KNPPHLWEGI  PKNTIFHPSK
bbbbbbbbbb  bbebbbbbbe  eebeebbbeb  eeeeebbeeb  eebebeeeee
s      s          s        f    f   ffff s f    f      ff

451         461         471         481         491
SKVLNMETCT  EWTRVLSWPG  DAEGRGVGCT  SEAFSQSSWI  RLTMRKDDDN
eebbebebbe  ebbebbbbee  eeeeebeebe  eebbeebbbb  bbbbeeeeee
 f  f          fss  f       f  fs    f    s

501         511         521         531         541
DSVSSTQLMD  IVTRLSARSK  AKVMFGSIFS  IRRKLAPSPA  FHSLGSFRAN
eeeebeebbe  bbbbbbeebe  bebbbbbbbb  beeeeeeeeb  beebbbbebe
               s   f     f  s    s   ff  f        fss  fs

551         561         571         581         591
YALQALITRG  SVFTDQLFDA  TDENIPSSDN  DNDEDDDDDV  DDTKKPMELV
bbbebbbeeb  bbbbeebbeb  beeeeeeeee  eeeeeeeeee  eeeeeeeeee
s  fs  ffs      ffs f                 f              f
```

```
601          611          621          631          641
HEPLFLKLVR   RGMKECSQAT   EETLEQLLNA   FDERRQIDVV   TAFTTMYQSR
eeebbbebbe   ebbeebeeeb   eebbeebbeb   beeeebbebb   bbbbebbebe
  f s f      f s fs  f    ff  ff sf     ff f         ss      f

651          661          671          681          691
KIQYERLLKG   ESLQDVGLAK   PLPKNCVSVA   KVIVTPSRIL   LMAPEVMMVN
eeebeebeee   eebeebebbe   eeeeebbebb   ebbbbbbbbb   bbbbebbbbb
  fs f       f  f           f fss s    f  s s s s    s sfsss s

701          711          721          731          741
RVVRRFGPDY   ALRCVFRDDN   LGRLAIRDFS   INNIDHMSNI   VTEGIYLTLK
bbbeebbeeb   bbebbbeeee   eeebbbeebb   bebbebbbbb   bbebbeebbe
s sf ss f    ssf s ffff     fs   fs      ssf               s

751          761          771          781          791
NGIQVADRVY   SFLGWSNSQM   RDQGCYLYAP   RVNALTGEVT   GTVEDIRVWM
ebbebbeebb   bbbbbeeeeb   eeebbbbbbe   eeeebebeee   bebeebeebb
       f s    ss  ffffs   fffsss s           s       f f sf  s

801          811          821          831          841
GDFRDAISVP   KMMSRMGQCF   TQAQPTVRLE   RHHWIVEPDI   EGGVENKYCF
bbbeebeebb   ebbbebbebb   eebeeebebe   eeebbbebeb   eeeeeeeebb
s s    fss   f ssfssfss   ffsfffs s          f f     ff      s

851          861          871          881          891
SDGCGRISIK   LATHISKILQ   LKEVPACFQV   RFKGFKGILV   IDPTIDDIIN
ebeeeebbbe   bbeebbebbe   beebeebbeb   ebeebebbbb   bbbbbeebbe
fsf ff s     ss      s    sf  ffs fs   fsffsfs      ss   f

901          911          921          931          941
MPKVIFRKSQ   QKFGEGGGEL   QDEYLEVVKY   AMPSPVCLNR   PFITILDQVS
bbebbbeeee   eeeeeeeeee   eeebbebbee   bbeeebbbee   ebbbbbeebe
 s  ssffff    ff              sfssff   ssfff s ff   f  sssf sf

951          961          971          981          991
EKQSASSHRR   ITNRVHYYLE   RELCSLSNML   INENQAAEEL   VNRTNLAIDW
eeeeeebeee   bbeebbbbbe   eebbebbebb   beeeebbeeb   beebebebeb
 fff   f        f ssssf   ffs   sfs     ff  s   s    ff  s  f

1001         1011         1021         1031         1041
NAASKRAGFE   LSVDPLIRDM   LFSIYRYNII   HHISKAKIFL   PPSLGRSMYG
bebbeebebe   beeeebbebb   bbbbbbbbbb   bbbbebebbb   beebbebbbb
       fs       f   f s       sss       s  fsf      s   sfss s

1051         1061         1071         1081         1091
VVDETGLLQY   GQVFIQYSPS   IRQTSNRPIL   KTGKVLITKN   PCHVPGDVRV
bbeeebbbeb   bebbbebbee   beeeeeeebb   eebebbbeee   eeebeeebeb
s fffsssf    sfsssfs       f  f  f       s  s fff   fff fff fs

1101         1111         1121         1131         1141
FDAVWQPALA   HLVDVVVFPQ   HGPRPHPDEM   AGSDLDGDEY   SIIWDQEMLL
beebbbebbe   ebbebbbbbe   ebeeeeeeeb   bbeebeeeeb   bbbbeeebbb
s fs sf s    fs fs sss    fs fff ffs   ssffsffffs     s ff sss

1151         1161         1171         1181         1191
DYNEEAMVFP   SSSAAEEDKE   PTTDDMVEFF   LRYLQQDSIG   RMSHAHLAYA
ebeeebbbbb   eeeeeeeeee   eeeeebbebb   bebbeeebbe   ebbbbbbbbb
 sff ss  s   ff           ff  f  fss   sfs f fs f     s sss  s
```

```
1201        1211        1221        1231        1241
DLHGLFHENC  HAIALKCAVA  VDFPKSGVPA  EPLSSFEQCE  MTPDYMMSGG
ebeebbeeeb  ebbbbebbbb  bebeeeeeeb  eebeeeeebe  eeeeebbeee
f  fss   s     s fs  s  sfsffff fs  f s   ff      ff      

1251        1261        1271        1281        1291
KPMYYSTRLN  GQLHRKARKV  EEVLEEFETR  GSVFEREYDK  LICPEDVDVF
eeeeebbebe  eebeeebeeb  eebbeebeee  eebbeeebee  bbbeeebebb
f  f s fsf  ffs f  ff   ff  f        f s   sf          fs 

1301        1311        1321        1331        1341
FGNEIKLVQT  LTLRDEYVDR  MQQLLDEYGI  EDEASVVSGH  AASIKRLAGM
beeeeeebeb  bebeeebbee  beebbeeebb  eeebbbbbee  bbbbeebeeb
               ff s      ffs fffss    fs  ssf       ffsf s

1351        1361        1371        1381        1391
ERDDYSFYHT  DKVVELRYEK  LYAVFRAKFF  EEFGGEEINI  ENDGKNTRLK
eeeebbbeeb  eebbebebee  beebbebebb  eebeeeeeee  eeeeeeeebe
ffffsssffs  ff sfsfsff       f  ss  ff f f              

1401        1411        1421        1431        1441
CTKAMHEKIR  QWYFVAYVQP  KINKAGRCIG  QSLPWVAWDA  LCDLRRQLML
beeebeeebe  ebbbbbbbee  eeeeeeeeeb  ebbebbbbeb  bbebeeebbe
   fs ff f  f s   s       f         fs          s  sff    

1451        1461        1471        1481        1491
DKNDAVLRGK  YPIAARLEEE  IENSIERQFD  KFLKLKDLIE  SHKDALFLRR
eeeeebeebe  bebbeebeee  beebbeeeee  ebeebeeebe  eeeebebbee
             f       f                                   f

1501        1511        1521        1531        1541
YVYFYGDQII  KMLFILKVWL  ERENVLPSSV  LSIWQLGRLL  IRLGLGDLLG
bbebebeebb  ebbbbbeebb  eeeebbeeeb  bbbbbbbbbb  bbbbbbebee
s   f          s     s                     s        ss    

1551        1561        1571        1581        1591
NPTIDYEKSL  LMPTTMFQQW  ISKKEDADEA  PILRNFDMGT  MMLEFLRYLA
eebbebeeeb  beeeebbeeb  beeeeeeeee  ebbbebbbbb  bbbbbbebbb
f  s                                        s       ss s s

1601        1611        1621        1631        1641
SQSFASAESI  SLRVFYEKDI  VEPILTKSAQ  WMPLHLIAYR  TFHSIAVSGR
eebbebbeeb  bbebbeeeeb  eeebbbebbe  bbebbbbbbe  bbbbbbbbbe
f  s         s            f s                   s s    ss 

1651        1661        1671        1681        1691
FDALHLDDED  AVDQITESKD  PILVNESLFS  SRNYNDDYPI  SRSRILQSLK
bebbebeeee  beeebeeeee  ebbbbeebbe  eeeeeeeeee  bebebbebbe
s                 ff    f  s    s                       sf

1701        1711        1721        1731        1741
DWSGVKEIIP  REITGTRKSD  MIYVTSVGTV  LARQRLARLL  LLSGETIRDA
ebeebeebbe  eebeeeeeee  ebbbbbbbbb  bbeebbbebb  bbeeeebeeb
  ffs       f  f     f     ss  ss       ss fs            s

1751        1761
IANNVVPNEV  RDEFL
bbeeebeeeb  eeeee
      f      f
```

The conservation scale:

1 2 3 4 5 6 7 8 9

Variable **Average** **Conserved**

e - An exposed residue according to the neural network algorithm.
b - A buried residue according to the neural network algorithm.
f - A predicted functional residue (highly conserved and exposed).
s - A predicted structural residue (highly conserved and buried).
x - Insufficient data - the calculation for this site was performed on less than 10% of the sequences.
