# Supplementary material for: An evolutionarily conserved N-terminal domain of RRF-3 governs GTSF-1 binding in nematodes
Source: EMBO Rep. 2026 Aug 29;27(19):5530–60. doi: 10.1038/s44319-026-00914-1 (PMC13650160; doi:10.1038/s44319-026-00914-1)
Supplement: Supplementary file 8 [file 44319_2026_914_MOESM6_ESM.zip › Figure 1 Source Data/1F_EV1C/Fig1F_abundance_boxplot.pdf]

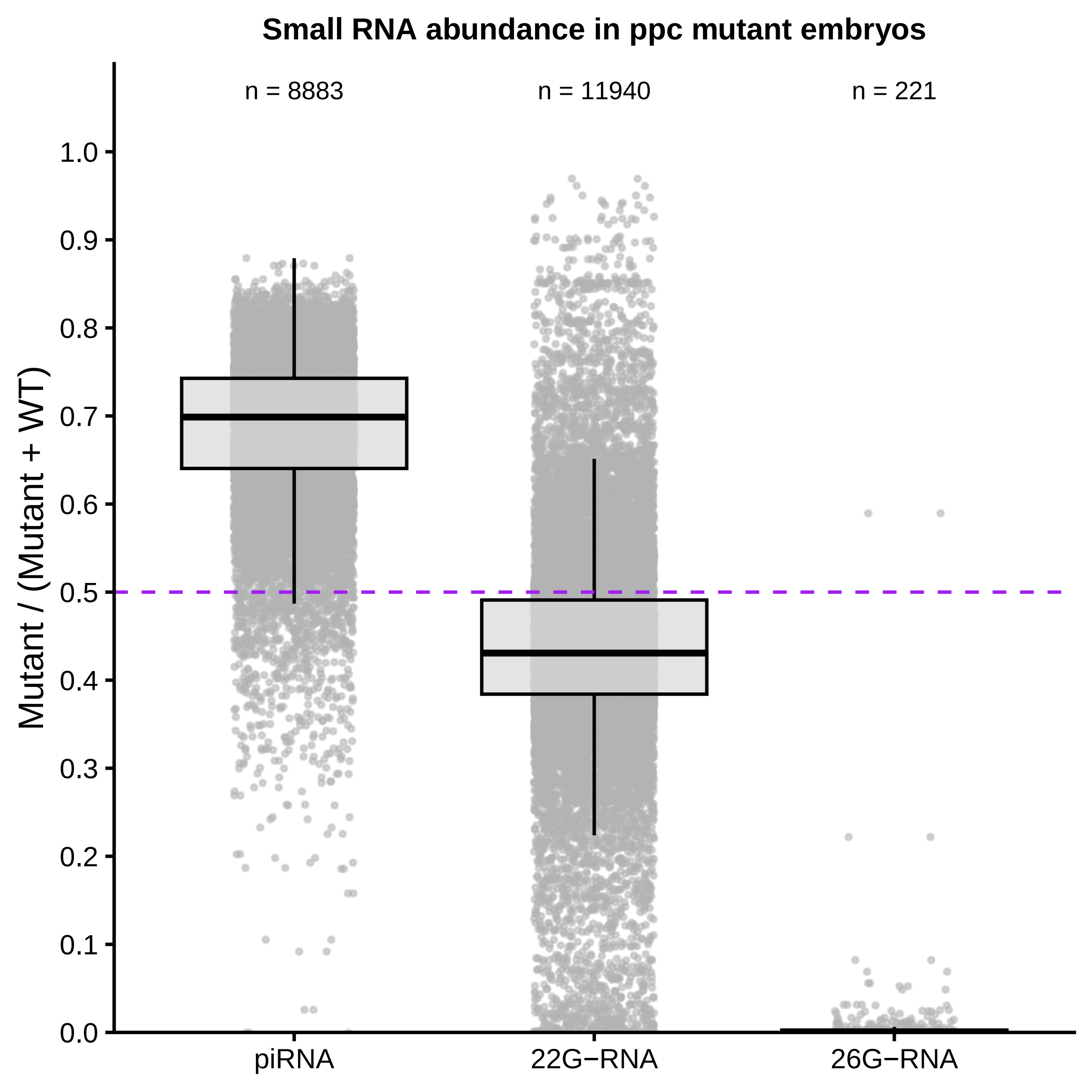

Small RNA abundance in ppc mutant embryos
n = 8883
n = 11940
n = 221
Mutant / (Mutant + WT)
1.0
0.9
0.8
0.7
0.6
0.5
0.4
0.3
0.2
0.1
0.0
piRNA
22G−RNA
26G−RNA
