# Supplementary material for: An evolutionarily conserved N-terminal domain of RRF-3 governs GTSF-1 binding in nematodes
Source: EMBO Rep. 2026 Aug 29;27(19):5530–60. doi: 10.1038/s44319-026-00914-1 (PMC13650160; doi:10.1038/s44319-026-00914-1)

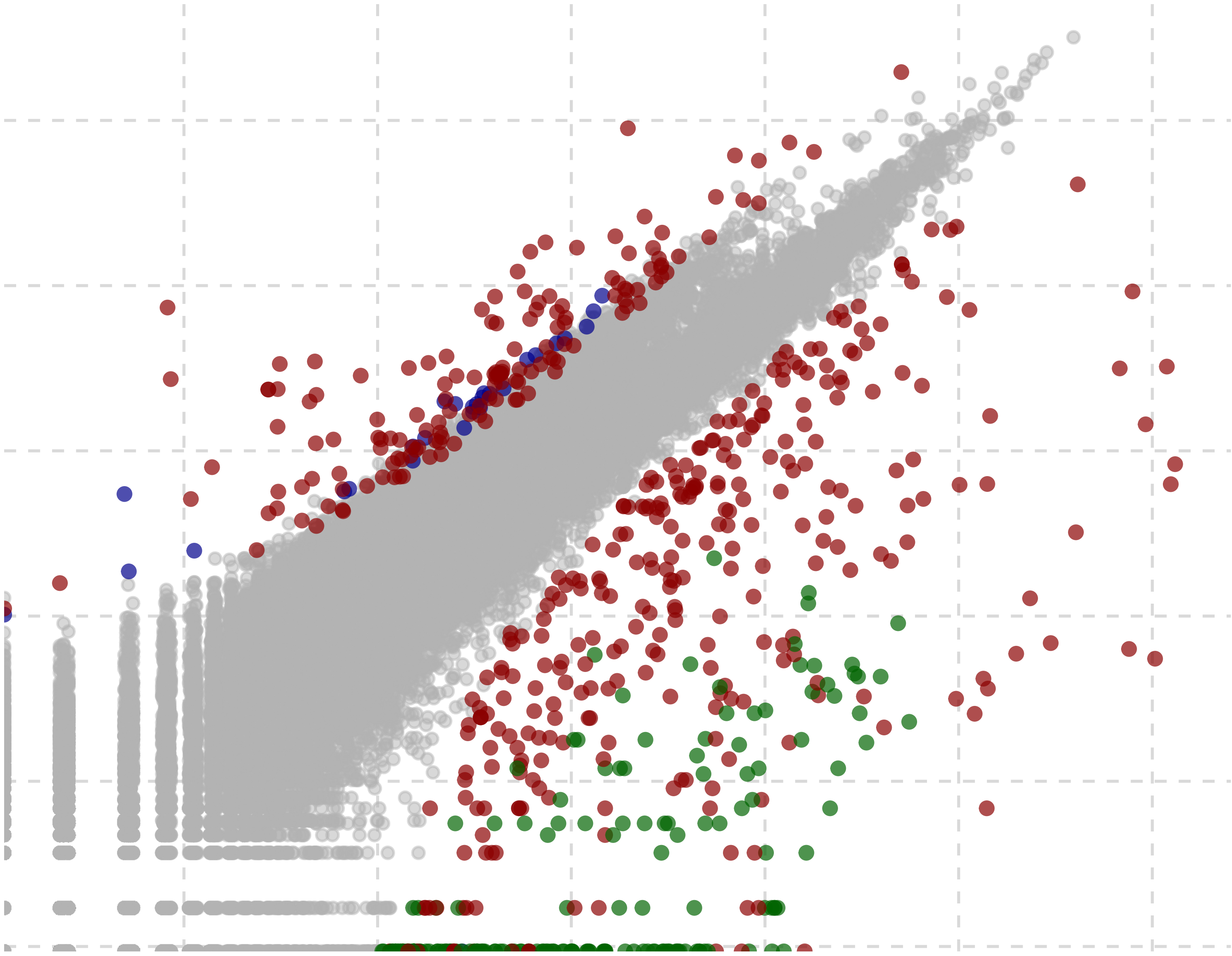

Supplement: Supplementary file 8 [file 44319_2026_914_MOESM6_ESM.zip › Figure 1 Source Data/1F_EV1C/Fig1_scatter_DGEbyClass_emb.pdf]
