# Supplementary material for: An evolutionarily conserved N-terminal domain of RRF-3 governs GTSF-1 binding in nematodes
Source: EMBO Rep. 2026 Aug 29;27(19):5530–60. doi: 10.1038/s44319-026-00914-1 (PMC13650160; doi:10.1038/s44319-026-00914-1)
Supplement: Supplementary file 9 [file 44319_2026_914_MOESM7_ESM.zip › Figure 2 Source Data/2B/2B_Left_P13_20230712_Ppacificus_gtsf1/user_report.html]

GTSF-1 IP in WT vs. RFK950


# GTSF-1 IP in WT vs. RFK950

- 1 Analysis details
- 2 Results
- 3 analysis figures
- 4 Table output

# 1 Analysis details

The databases used as search space for this MaxQuant analysis where
the following:

```
## [1] "D:/Fastafiles/Escherichia.coli_uniprot_Ref2023-01_5064seq.fasta"       
## [2] "D:/Fastafiles/Pristionchus.pacificus_uniprot_Ref2023-03_26122seq.fasta"
```

The MaxQuant version 2.1.3.0 was used. Beside the standard settings
in this version, the following changes were applied:

- Match between runs was activated
- LFQ with minimum 1 ratio count
- NO fast LFQ
- **NO** LFQ normalization

# 2 Results

## 2.1 Proteins detected

The data was first filtered by removing 144 E. coli protein groups,
all reverse database binders, potential contaminants and proteins only
identified by site modification. Additionally we filtered for minimum 1
`Razor + unique peptide` (minimum 1
`Unique peptide`). This dataset with 1963 protein groups is
saved in proteinGroups\_filtered.xlsx excel file in the
`full filtered MaxQuant output` tap.

We highlighted `GTSF-1` (A0A8R1V7T7).

To show the difference between the individual samples, we visualize
the quantified protein group count per replicate in an extra plot.

## 2.2 Reproducibility

After carefully checking the data, we decided to *median
center* the data for normalization.

## 2.3 missing values imputation

After normalization we used a normal distribution to impute missing
values. This was performed per replicate by shifting the median of the
measured values by -3.5 and shrinking the standard deviation by a factor
of 0.2.

# 3 analysis figures

For the volcano plots, we added an additional filtering step to have
at least 2 LFQ.intensities measured in ether `DRSH1` or
`WT`. The complete list of proteins and the data to reproduce
the plots is listed in individual excel files named
`volcano_data_*.xlsx`. We used a as a cut-off t0 of 1 and s0
of 1.5.

## 3.1 WT/noGTSF1

For easier exploration of the data, we added an interactive plot. By
hoovering over a specific protein, you will get information like
`Protein.IDs`, `Gene.name` and counting the
detected replicates for respective site.

# 4 Table output

We created the proteinGroups\_filtered.xlsx file as Excel file with
all MaxQuant columns for you to explore the plain data. We added the
columns with the normalised values to the end of the file.

Additionally, we created a reduced file for each indiviual volcano
plot (`volcano_data_*.xlsx`). These files have all
statistical information needed to reproduce these figures. The LFQ
columns shown in this table use the already normalised data and not the
original values from the proteinGroups file.
