# Supplementary material for: An evolutionarily conserved N-terminal domain of RRF-3 governs GTSF-1 binding in nematodes
Source: EMBO Rep. 2026 Aug 29;27(19):5530–60. doi: 10.1038/s44319-026-00914-1 (PMC13650160; doi:10.1038/s44319-026-00914-1)
Supplement: Supplementary file 9 [file 44319_2026_914_MOESM7_ESM.zip › Figure 2 Source Data/2D/user_report_DIA_2.0.19.html]

RRF3 IP in GTSF1 deletion


# RRF3 IP in GTSF1 deletion

- 1 Analysis details
- 2 Results
  - 2.1 Proteins detected
  - 2.2 Protein abundance
  - 2.3 Reproducibility
- 3 analysis figures
  - 3.1 GTSF1del/WT
  - 3.2 GTSF1del/WThigh
  - 3.3 WThigh/WT
- 4 Table output

# 1 Analysis details

The analysis was done using DIA-NN (version: 2.2.0) and the following
databases where used as search space:

- Cbr\_3xMYC\_RRF3.fasta
- Caenorhabditis.briggsae\_uniprot\_Ref2024-03\_21756seq.fasta
- Escherichia.coli\_uniprot\_Ref2024-03\_4416seq.fasta
- MQ\_contaminants\_processed.fasta

# 2 Results

The results for this report are located in the following folder (for
internal reference only):  
*20250624\_Shamitha\_RRF3\_IP*

## 2.1 Proteins detected

The data was filtered by removing **13 E. coli
proteins** and potential contaminants and has
**3139** protein groups.

## 2.2 Protein abundance

The next plot show the top detected proteins per sample and also the
highlighted proteins with their abundance.

To show the difference between the individual samples, we visualize
the quantified protein group count per replicate.

## 2.3 Reproducibility

The samples where normalized on `Cbr_3xMYC_RRF3` and show
a high reproducibility throughout the conditions.

**NOTE:** For the following statistical analysis, we
need to add a little noise to the bait protein, otherwise the analysis
would fail.

A Principal Componant Analysis was performed to show the separation
of the samples based on variations.

# 3 analysis figures

We added an additional filtering step to have at least 3
LFQ.intensities measured in either sample. Missing values where imputed
close to the detection limit for each replicate individually.  
The complete list of proteins and the data to reproduce the plots is
listed in individual excel files named
`limma_results_*.xlsx`. We used as cut-off: 0.05 minimum
p-value and 1 minimum log2 foldchange. The p-values are
**Benjamini-Hochberg** corrected for multiple testing. The
fitting method within `limma` was set to “least square”.

## 3.1 GTSF1del/WT

For easier exploration of the data, we provide interactive plots in
extra files for each volcano plot
(`interactive_limma_volcano_.*.html`). By hoovering over a
specific protein, you will get information like `Gene.name`
or `Protein.name`.

## 3.2 GTSF1del/WThigh

## 3.3 WThigh/WT

# 4 Table output

We created files for each indiviual volcano plot
(`limma_result_*.xlsx`). These
files have all statistical information needed to reproduce these
figures. The LFQ columns shown in this table use the already normalised
data and not the original values from the proteinGroups file.
